# Supplementary material for: Enzymatic Macrocyclization of 1,2,3‐Triazole Peptide Mimetics
Source: Angew Chem Weinheim Bergstr Ger. 2016 Apr 5;128(19):5936–9. doi: 10.1002/ange.201601564 (PMC4924595; doi:10.1002/ange.201601564)
Supplement: Supplementary file 1 — Supplementary [file ANGE-128-5936-s001.pdf]

Angewandte Chemie
Eine Zeitschrift der Gesellschaft Deutscher Chemiker

Supporting Information

# Enzymatic Macrocyclization of 1,2,3-Triazole Peptide Mimetics

*Emilia Oueis, Marcel Jaspars, Nicholas J. Westwood, and James H. Naismith**

ange_201601564_sm_miscellaneous_information.pdf

I. STRUCTURES OF PRECURSOR PEPTIDES P AND CYCLIC PEPTIDES CP................ S2

II. GENERAL INFORMATION AND MATERIALS .................................................. S4

III. GENERAL PROCEDURES ...................................................................... S6

IV. MS AND HPLC DATA OF STARTING AND FINAL CYCLIC PEPTIDES...................S14

V. NMR DATA OF FINAL CYCLIC PEPTIDES ........................................................S16

VI. NMR SPECTRA OF FINAL CYCLIC PEPTIDES ...................................................S33

VII. EXSY NMR OF FINAL CYCLIC PEPTIDES .........................................................S42

VIII. MS-MS DATA OF FINAL CYCLIC PEPTIDES.....................................................S47

IX. LC-MS TRACES OF CRUDE MIXTURES OF CP3, CP13, AND CP135 ...................S52

X. LC-MS TRACES OF FINAL CYCLIC PEPTIDES ...................................................S55

XI. LC-MS TRACES OF STARTING PEPTIDES........................................................S59

XII. REFERENCES ..........................................................................................S69

# I. Structures of precursor peptides P and cyclic peptides CP

## 1. Precursor peptides P

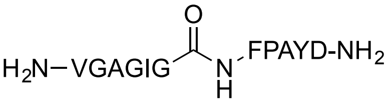

**P0**

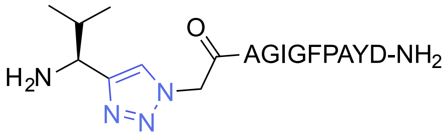

**P1**

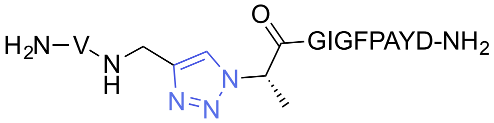

**P2**

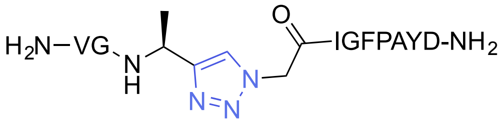

**P3**

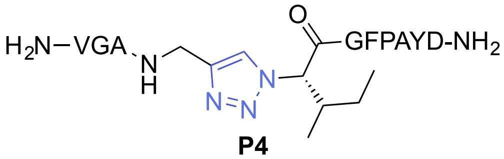

**P4**

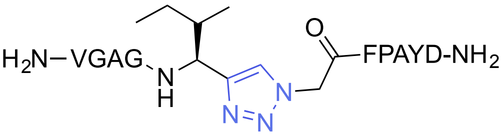

**P5**

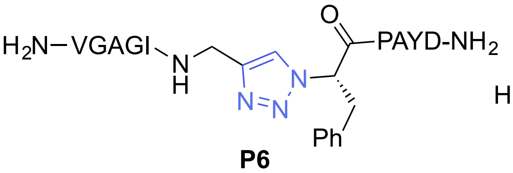

**P6**

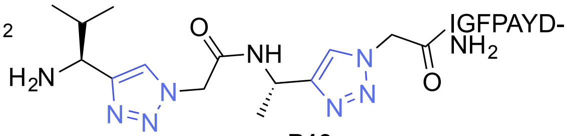

**P13**

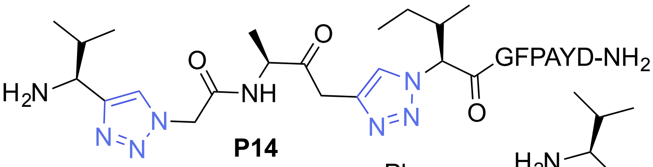

**P14**

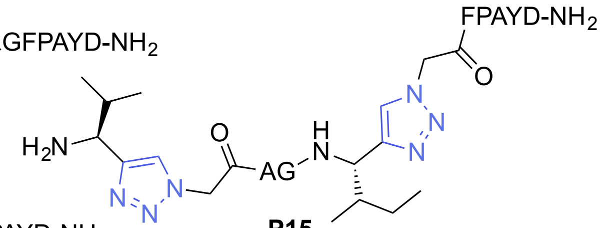

**P15**

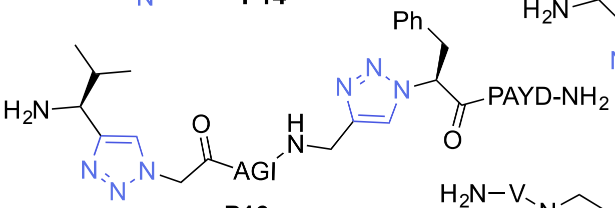

**P16**

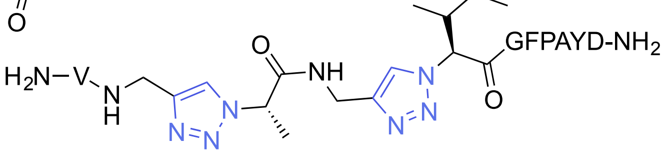

**P24**

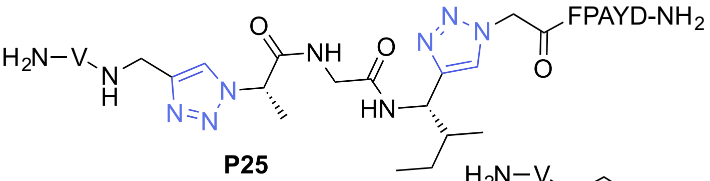

**P25**

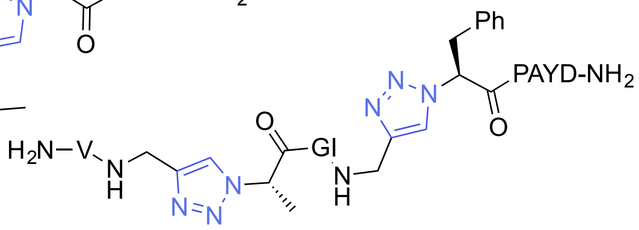

**P26**

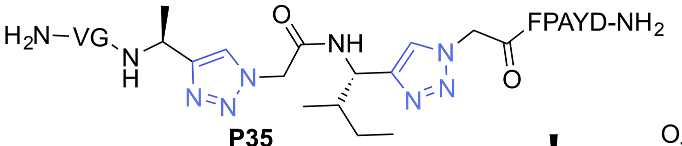

**P35**

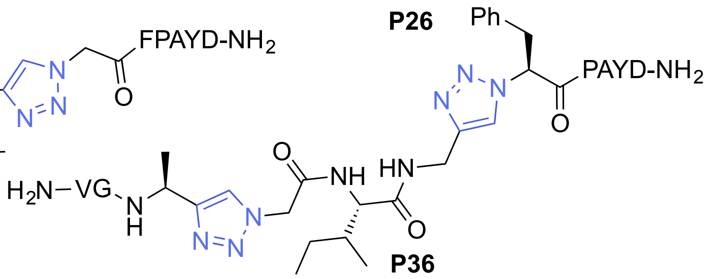

**P36**

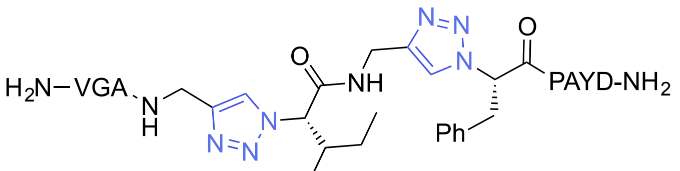

**P46**

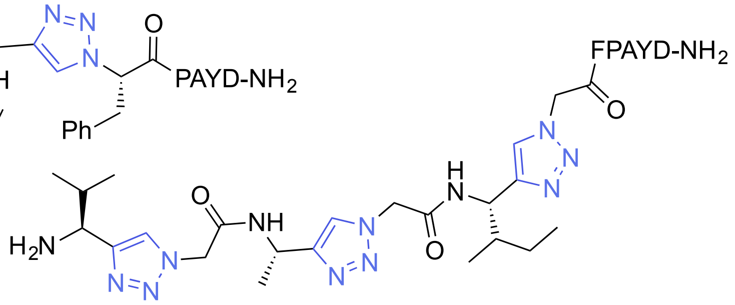

**P135**

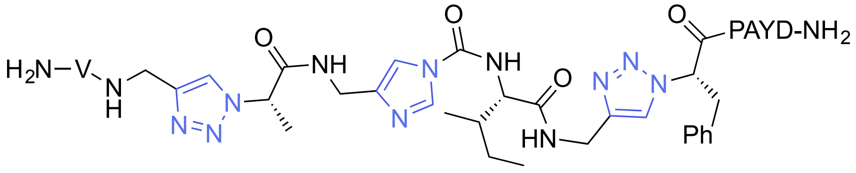

**P246**

## 2. Cyclic peptides CP

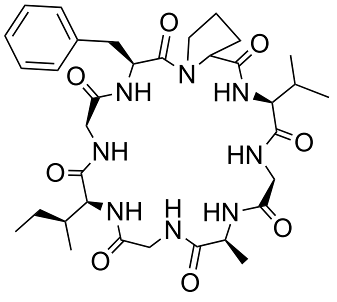

**CP0**

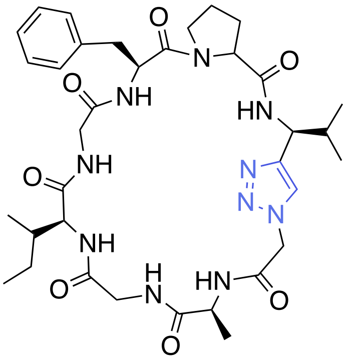

**CP1**

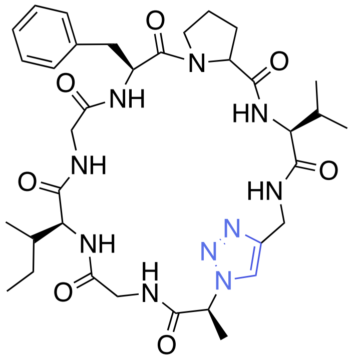

**CP2**

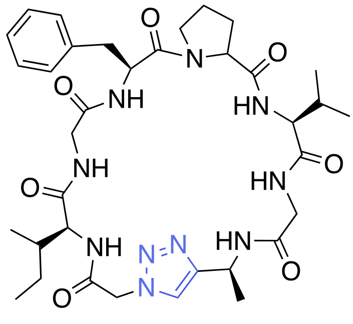

**CP3**

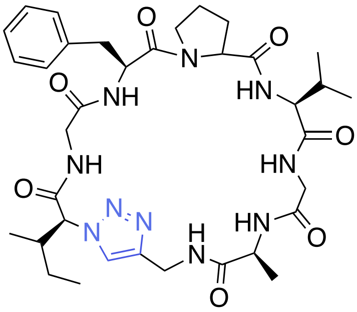

**CP4**

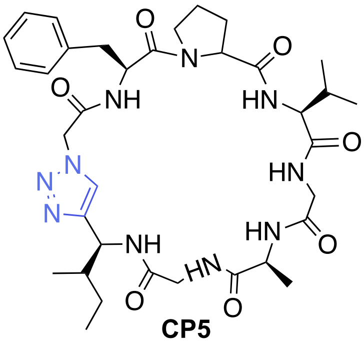

**CP5**

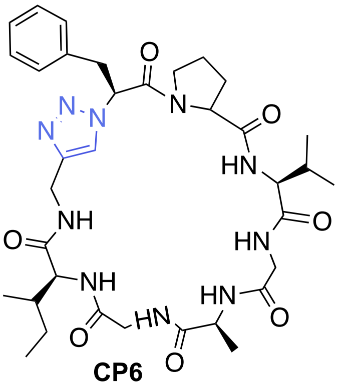

**CP6**

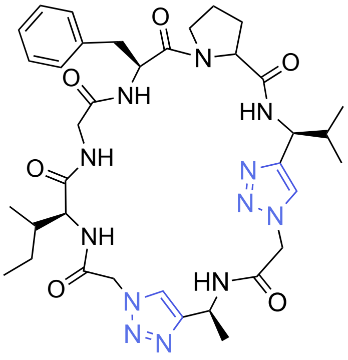

**CP13**

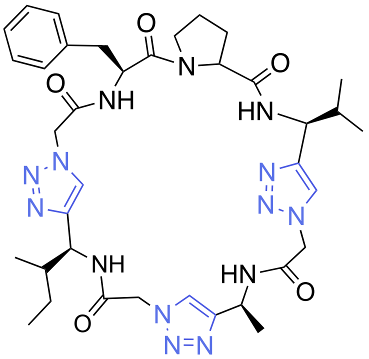

**CP135**

# II. General information and materials

The HPLC grade acetonitrile (MeCN) was purchased from Fisher. Aqueous buffers and aqueous mobile-phases for HPLC were prepared using water purified with an Elga® Purelab® Milli-Q water purification system (purified to 18.2 MΩ.cm). DMSO, THF and dichloromethane ($CH_2Cl_2$) were purchased from Fisher. Amino acids and coupling reagents were purchased commercially from different sources. Other chemicals and reagents were purchased from Sigma and used without any further purification.

NMR spectra ($^1$H, 2D) were recorded on a Bruker Ascend 700 spectrometer {δH (700 MHz), δC (175 MHz)} at ambient temperature in the deuterated solvent stated. Chemical shifts are expressed in parts per million (ppm) from DMSO-d6 (δH = 2.50).[1] Multiplicities are described as s (singlet), d (doublet), q (quadruplet), qt (quintuplet), dd (doublet of doublets), t (triplet), m (multiplet), br (broad). Coupling constants *J* are quoted in Hertz (Hz) to the nearest 0.1 Hz. Signals of protons and carbons were assigned, as far as possible, by using the following two dimensional NMR spectroscopy techniques: [$^1$H, $^1$H] COSY (Correlation Spectroscopy), [$^1$H, $^1$H] TOCSY (Total Correlation Spectroscopy), [$^1$H, $^1$H] ROESY (Rotating-frame NOE Spectroscopy), [$^1$H, $^{13}$C] HSQC (Heteronuclear Single Quantum Coherence) and long range [$^1$H, $^{13}$C] HMBC (Heteronuclear Multiple Bond Connectivity). EXSY (Exchange Spectroscopy) experiment was used to identify equilibrium chemical exchange either at rt or with heating.

Low-resolution mass spectra were obtained with an Agilent 6130 single quad apparatus equipped with an electrospray ionization source. High-resolution mass spectra (HRMS) were obtained with a Thermo Exactive Orbitrap mass spectrometer.

Reactions performed in the enzymatic media were monitored using MALDI-MS acquired using a 4800 MALDI TOF/TOF Analyser (ABSciex, Foster City, CA) equipped with a Nd:YAG 355 nm laser and calibrated using a mixture of peptides. The spot was analysed in positive MS mode between 500 and 4000 m/z, by averaging 1000 laser spots. The samples, diluted in water to reduce the buffer concentration, (0.5 µL) were applied to the MALDI target along with alpha-cyano-4-hydroxycinnamic acid matrix (0.5 µL, 10 mg/mL in 50:50 acetonitrile:0.1% TFA) and allowed to dry. MSMS data were acquired using a TripleTOF 5600+. The sample was

subjected to chromatography on an Acclaim PepMap 100 C18 trap and an Acclaim PepMap RSLC C18 column (ThermoFisher Scientific), using a nano-LC Ultra 2D plus loading pump and nano-LC as-2 autosampler (Eksigent). The sample was injected at neutral pH to avoid acid catalyzed ring opening. The trap was washed with 2% acetonitrile, 0.05% trifluoroacetic acid, and the patellamide was then eluted with a gradient of increasing acetonitrile, containing 0.1 % formic acid (15-40% acetonitrile in 5 min, 40-95% in a further 1 min, followed by 95% acetonitrile to clean the column, before re-equilibration to 15% acetonitrile). The eluent was sprayed into a TripleTOF 5600+ electrospray tandem mass spectrometer (Sciex) operating with standard nanospray conditions, and analyzed in Product Ion Scan mode isolating the m/z of interest. The collision energy was adjusted to give optimal fragmentation. The MSMS fragmentation pattern was interrogated for diagnostic peaks.

Automated solid-phase peptide synthesis (SPPS) was accomplished on a Biotage® Syro Wave™ system in polypropylene (PP) syringe with a PTFE frit. Manual SPPS was performed using a vacuum manifold and a shaker. Final cleavage and deprotection were completed manually.

Analytical RP-HPLC was performed on an Agilent infinity 1260 series equipped with either a VWD or an MWD detector and a single quadrupole MS using a Macherey-Nagel Nucleodur C18 column (10 µm x 4.6 x 250 mm) using the following chromatographic system: 1 mL/min flow rate with MeCN and 0.1 % aqueous TFA [95% TFA (5 min), linear gradient from 5 to 95% of MeCN (35 min), 95% MeCN (40 min)] and UV detection at 220 nm.

Semi-preparative RP-HPLC was performed on an Agilent infinity 1260 series equipped with a VWD or an MWD detector using a Macherey-Nagel Nucleodur C18 column (10 µm x 10 x 250 mm at 4 mL/min, a 10 µm x 16 x 250 mm at 10 mL/min, or 10 µm x 21 x 250 mm at 16 mL/min). Several chromatographic systems were used; System Pa: MeCN and 0.1 % aqueous TFA [95% TFA (5 min), linear gradient from 5 to 95% of MeCN (35 min), 95% MeCN (40 min)] and UV detection at 220 nm. System Pb: MeCN and 0.1 % aqueous TFA [95% TFA (5 min), linear gradient from 5 to x-3% of MeCN (1% /min), x+12% MeCN (0.1% /min)] and UV detection at 220 nm; where x is the % of MeCN at which the corresponding peak elutes on the analytical run of a similar column type. The runs were stopped as soon as the peptide eluted off the column.

# III. General procedures

***Pat*$G_{mac}$ cloning, expression and purification:**

The *Pat*$G_{mac}$ enzyme was cloned from genomic DNA *(Prochlon sp.*) into the pHISTEV vector, expressed in *Escherichia coli* BL21 (DE3) cells grown on autoinduction medium, and purified as previously described by Koehnke *et al.*.[2] However, subsequent to the Nickel column eluting with 250 mM imidazole, the remaining purification steps were replaced with dialysis in a bicine buffered solution [20 mM Bicine, 150 mM NaCl, pH = 8.1] to remove the imidazole and the reducing agent.

**Solid-phase peptide synthesis**

The different precursor peptides were synthesized on solid-phase (SPPS) on a Rink amide resin (RAR) using the Fmoc strategy and Fmoc-protected amino acids (aa). Both automatic (A) and manual (M) procedures were employed. The introduction of the triazole moiety was accomplished by first reacting azido acids to the growing peptide (A or M) and then by manually introducing the triazole through a copper-catalyzed azide alkyne cycloaddition (CuAAC) between the azide-terminal peptide and the Fmoc-protected alkyne amino acid analogue (Scheme S1).

**1- Resin preparation**

The rink amide resin (RAR) is weighed into the fritted PP-syringe then swelled in DMF for half an hour.

**2- Automated SPPS**

A double coupling strategy was used for all amino acids using the following conditions for each step:

5 eq. HBTU in DMF; 8 eq. DIEA in NMP; 5 eq. aa in DMF Reaction time of 30 min with mixing/shaking for 15 s every two minutes at rt followed by 4x DMF washing cycles.

Fmoc deprotection is done in 40% piperidine/DMF in two steps:

3 min reaction with mixing/shaking for 10 s every minute at rt then 12 min reaction with mixing/shaking for 10 s every two minutes at rt followed by 6x DMF washing cycles.

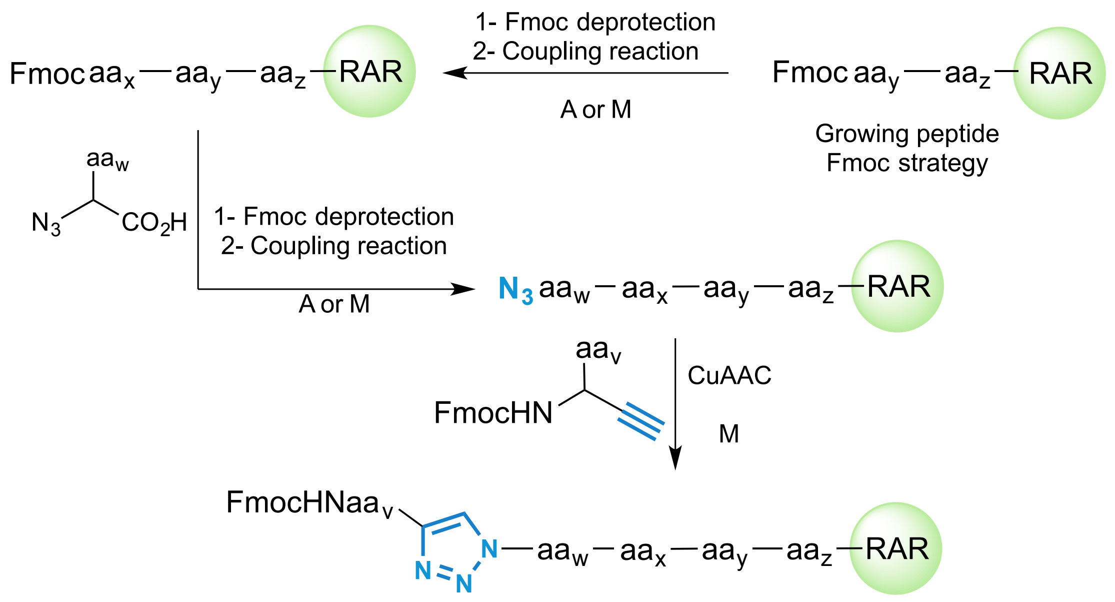


Scheme S1: General SPPS strategy for the synthesis of triazole-containing peptides.

**3- Manual SPPS**

A double coupling strategy was used for all amino acids using the following conditions for each step:

5 eq. HBTU in DMF; 10 eq. DIEA in NMP; 5 eq. aa in DMF Reaction time of 1h 30 min with shaking at rt followed by 4x DMF, 4x $CH_2Cl_2$, and 4x DMF washing cycles. Fmoc deprotection is done in 20% piperidine/DMF for 10 min followed by 4x DMF, 4x $CH_2Cl_2$, and 4x DMF washing cycles.

**4- Manual CuAAC reaction; solid phase (SP)**

After the coupling of an azido acid to the growing peptidic chain (A or M), the resin is washed with 5x THF. The corresponding amino alkyne (5 eq.) is diluted in THF and added to the resin. DIEA (10 eq.) is then added followed by CuI (5 eq.). The mixture is bubbled with dry nitrogen for 1 min then sealed, protected from light, and left overnight with shaking. After the reaction is complete (mini work up of a few

beads; repeat the procedure if reaction not complete), the resin is washed with 4x THF, 4x $H_2O$, 4x THF, and 4x DMSO. The resin is swelled in DMSO for 10 min before the Fmoc deprotection step (if needed) with 20% piperidine/DMF for 10 min followed by 4x DMF, 4x $CH_2Cl_2$, and 4x DMF washing cycles.

#### 5- Final cleavage

The resin is washed with $CH_2Cl_2$ and dried for a minute on the vacuum manifold. The beads are then transferred into a flacon tube and the cleavage cocktail is added: 95% TFA, 3.5% $H_2O$, 1.5% TIS. The mixture is vortexed to mix everything and the flacon tube is left on the shaker for 2h. The resin is then filtered, washed with 3x $CH_2Cl_2$ and the filtrate is evaporated under vacuum until a few milliliters of TFA are left (< 2mL).

#### 6- Peptide precipitation and HPLC purification

To a falcon tube containing cold diethyl ether, the remaining TFA solution is added dropwise. The peptide precipitates upon contact with the cold ether. The falcon tube is placed for 5 min in the cold and centrifuged at 4 °C and 4000 rpm for 10 min. The supernatant is decanted and the remaining solid left a few minutes under nitrogen flow to dry. The crude is then solubilized in a minimum volume of 20% acetic acid and MeCN (ultrasonic bath helps) and immediately purified by HPLC.
Peptides **P0**, **P13**, **P14**, **P15**, **P24**, **P25**, **P26**, **P35**, **P36**, **P46**, **P135**, and **P246** were purified using system Pa and peptides **P1**, **P2**, **P3**, **P4**, **P5**, **P6**, and **P16** were purified using system Pb.

### Pat Gmac macrocylisation reaction

The reactions are conducted in 20 mM bicine buffer, 500 mM NaCl, and 5% DMSO solution, pH = 8.1 and incubated at 37 °C (without shaking) until full consumption of the starting peptide (MALDI monitoring). The reaction set-ups are prepared in the following order; final concentrations:

1- A solution of the linear peptide in DMSO (between 10 and 50 mM); 100 µM
2- DMSO; 5%
3- 20 mM Bicine, 150 mM NaCl, pH=8.1 buffer

4- 5 mM NaCl; 500 mM

5- *Pat*$G_{mac}$ enzyme; 60 μM

The large scale reaction mixture is then extracted 3 times with *n*-butanol (BuOH): $H_2O$ (1/1, v/v). BuOH is added to the aqueous reaction, vigorously mixed, and then centrifuged for 10 min at high speed to help separate the two phases. The combined BuOH fractions are evaporated under reduced pressure to dryness. The crude is solubilized in a minimum volume of $H_2O$/MeCN and immediately purified by HPLC using system Pa.

## Synthesis of amino acids analogues

### 1- Synthesis of imidazole-1-sulfonyl azide hydrochloride

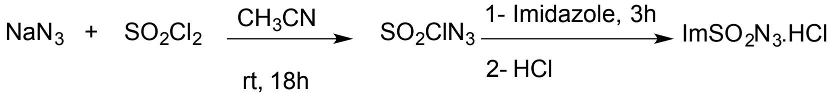


Scheme S2: Synthesis of the diazotransfer reagent.

A suspension of $NaN_3$ (2g, 0.03 mol) in anhydrous MeCN (30 mL) is cooled to 0 °C. Sulfuryl chloride (2.5 mL, 0.03 mol) is then added drop-wise and the mixture stirred overnight at room temperature. Imidazole (3.98 g, 0.06 mmol) is added slowly to the reaction at 0 °C and the mixture is stirred for 3 h at room temperature. EtOAc (60 mL) is added and the reaction is washed with $H_2O$ (2x 40 mL) and saturated aqueous $NaHCO_3$ (2x 40 mL). The organic fraction is then dried over $MgSO_4$ and filtered. In a separate flask, a solution of anhydrous HCl in EtOH is prepared at 0 °C by the dropwise addition of acetyl chloride (3.28 mL, 0.046 mol) to dry ethanol (7.5 mL).

The EtOAc solution is cooled to 0 °C and while stirring, the HCl solution in EtOH is added dropwise to precipitate a white solid. The precipitate is then filtered, washed with EtOAc (3x 15 mL), and dried under pressure to give imidazole-1-sulfonyl azide hydrochloride as white solid (5g, 76%). The NMR spectroscopic data were in agreement with those described in the literature.[3] **Caution!** Imidazole-1-sulfonyl azide and its salts (including chloride salt) are known to be energetic materials that are sensitive to chock and friction. Care should be taken while handling this material

and proper safety precautions should be used. Plastic container and materials are recommended for use.[4]

**2- General procedure for the synthesis of azido acids 1a, 1b, and 1d**

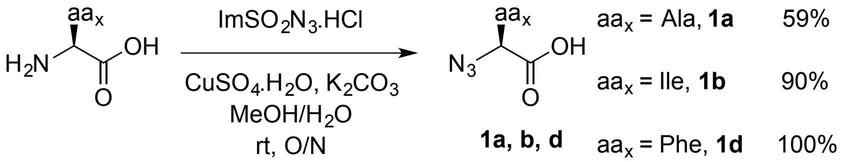


Scheme S3: Synthesis of azido acids **1**.

To a solution of the amino acid (1 eq.), $K_2CO_3$ (2.7 eq.), and $CuSO_4.5H_2O$ (0.01 eq.) in a MeOH/H2O mixture (5/1, v/v), $ImSO_2N_3$.HCl is added and the mixture stirred vigorously for 18h at room temperature. The reaction is then concentrated under reduced pressure, diluted with $H_2O$, acidified with 1M HCl to pH = 1, and extracted with 3x $CH_2Cl_2$. The organic layer is dried over $MgSO_4$, filtered, and concentrated. The crude obtained is then diluted in saturated aqueous $NaHCO_3$ and washed with $Et_2O$. The aqueous phase is acidified to pH with conc. HCl solution and extracted with 3x $CH_2Cl_2$. The combined organic layers are dried over $MgSO_4$, filtered, and concentrated. The corresponding azido acids are then used without any further purification.[3]

**1a**: *L*-Ala (1g, 11.2 mmol) in MeOH/$H_2O$ (50 mL/10 mL) was treated according to the abovementioned procedure. Compound **1a** was obtained as pale yellow oil (755 mg, 59%). The NMR spectroscopic data were in agreement with those described in the literature.[5]

**1b**: *L*-Ile (1g, 7.6 mmol) in MeOH/$H_2O$ (34 mL/7 mL) was treated according to the abovementioned procedure. Compound **1b** was obtained as colourless oil (1.03 g, 86%). The NMR spectroscopic data were in agreement with those described in the literature.[5]

**1d**: *L*-Phe (1g, 6.05 mmol) in MeOH/$H_2O$ (27 mL/5.5 mL) was treated according to the abovementioned procedure. Compound **1d** was obtained as colourless oil (1.1 g, 100%). The NMR spectroscopic data were in agreement with those described in the literature.[5]

**3- General procedure for the synthesis of amino alkynes 2a, 2b, and 2c[6]**

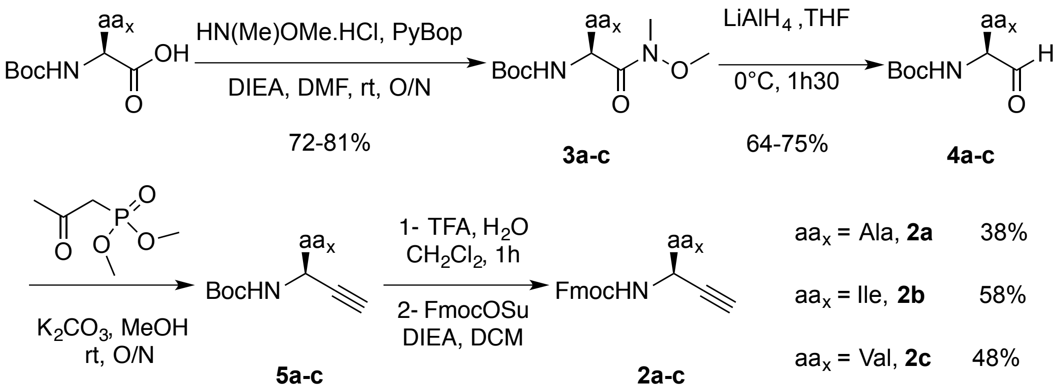


Scheme S4: Synthesis of Fmoc protected amino alkynes **2**.

**3a-c**: To a solution of BOC-protected amino acids (1 eq.) and DIEA (1.5 eq.) in DMF is added PyBop (1.1 eq.) and the reaction is stirred at rt for 0.5 h. Weinreb amine (1.1 eq.), DIEA (1.1 eq.) and DMF are then added to the reaction mixture and left at room temperature overnight. The reaction mixture is diluted with $H_2O$ and extracted with 3x EtOAc. The combined organic fractions are washed successively with saturated aqueous $NaHCO_3$, $KHSO_4$ 1M, and brine. The organic fraction is dried over $MgSO_4$, filtered, and evaporated under reduced pressure. Purification over silica gel afforded the corresponding weinreb amide **3a-c**.

**3a**: Boc-*L*-Ala (1.5g, 7.9 mmol) in DMF (16 mL; 10 mL) was treated according to the abovementioned procedure. Purification over silica gel (30% EtOAc/70% $CH_2Cl_2$) afforded compound **3a** as white solid (1.3 g, 72%). The NMR spectroscopic data were in agreement with those described in the literature.[7]

**3b**: Boc-*L*-Ile (1g, 4.3 mmol) in DMF (8.5 mL; 5.5 mL) was treated according to the above mentioned procedure. Purification over silica gel (15% EtOAc/85% $CH_2Cl_2$) afforded compound **3b** as colourless oil (964 mg, 81%). The NMR spectroscopic data were in agreement with those described in the literature.[7]

**3c**: Boc-*L*-Val (1g, 4.6 mmol) in DMF (9 mL; 6 mL) was treated according to the abovementioned procedure. Purification over silica gel (20% EtOAc/80% $CH_2Cl_2$) afforded compound **3c** as colourless oil (919 mg, 77%). The NMR spectroscopic data

were in agreement with those described in the literature.[7]

**4a-c**: To a solution of the Weinreb amides **3a-c** (1 eq.) in anhydrous THF at 0 °C, $LiAlH_4$ (1.2 eq.) is added slowly and the reaction is stirred at 0 °C for 1.5 h. The reaction mixture is quenched by the addition of a solution of HCl 0.1M and extracted with 3x EtOAc. The combined organic fractions are washed with brine, dried over $MgSO_4$, filtered, and evaporated under reduced pressure. Purification over silica gel afforded the corresponding aldehydes **4a-c**.

**4a**: Weinreb amide **3a** (116 mg, 0.5 mmol) in THF (1.5 mL) was treated according to the abovementioned procedure. Purification over silica gel (10% EtOAc/90% $CH_2Cl_2$) afforded compound **4a** as white solid (69 mg, 75%). The NMR spectroscopic data were in agreement with those described in the literature.[7]

**4b**: Weinreb amide **3b** (116 mg, 0.5 mmol) in THF (1.5 mL) was treated according to the abovementioned procedure. Purification over silica gel (5% EtOAc/95% $CH_2Cl_2$) afforded compound **4b** as colourless oil (66 mg, 69%). The NMR spectroscopic data were in agreement with those described in the literature.[7]

**4c**: Weinreb amide **3c** (121 mg, 0.5 mmol) in THF (1.5 mL) was treated according to the abovementioned procedure. Purification over silica gel (5% EtOAc/95% $CH_2Cl_2$) afforded compound **4c** as colourless oil (60 mg, 64%). The NMR spectroscopic data were in agreement with those described in the literature.[7]

**5a-c**: To a solution of the aldehyde **4a-c** (1 eq.) and $K_2CO_3$ (2 eq.) in anhydrous MeOH, the Bestmann-Ohira reagent (dimethyl (1-diazo-2-oxopropyl)phosphonate; 1.2 eq.) is added and the reaction is stirred at rt overnight. The reaction mixture is diluted with $Et_2O$ and washed successively with 5% aqueous $NaHCO_3$ and $H_2O$, dried over $MgSO_4$, filtered, and evaporated under reduced pressure. Purification over silica gel afforded the corresponding alkynes **5a-c**.

**5a**: Aldehyde **4a** (40 mg, 0.23 mmol) in MeOH (3.5 mL) was treated according to the abovementioned procedure. Purification over silica gel ($CH_2Cl_2$) afforded compound **5a** as white solid (24 mg, 61%). The NMR spectroscopic data were in agreement with those described in the literature.[8]

**5b**: Aldehyde **4b** (66 mg, 0.31 mmol) in MeOH (4.8 mL) was treated according to the abovementioned procedure. Purification over silica gel ($CH_2Cl_2$) afforded compound

**5b** as colourless oil. The NMR spectroscopic data were in agreement with those described in the literature.[8]

**5c**: Aldehyde **4c** (57 mg, 0.28 mmol) in MeOH (4.3 mL) was treated according to the abovementioned procedure. Purification over silica gel (10% pentane/90% $CH_2Cl_2$) afforded compound **5c** as white solid. The NMR spectroscopic data were in agreement with those described in the literature.[8]

**6a-c**: Alkyne **5a-c** (1 eq.) is diluted in $CH_2Cl_2$ (V) and TFA/$H_2O$ (95:5; 2V) is added and stirred at rt until full deprotection of the compound. The solvent is evaporated and resuspended in toluene and evaporated 3 times until dryness. The crude is diluted in $CH_2Cl_2$ (V) and DIEA (1.1 eq.) and FmocOSu (2 eq.) are added and the reaction stirred at room temperature overnight. The mixture is washed successively with 3x HCl 1M, 3x $NaHCO_3$, and brine. The organic layer is dried over $MgSO_4$, filtered, and evaporated under reduced pressure. Purification over silica gel afforded the Fmoc protected alkyne **2a-c**.

**2a**: Boc-protected alkyne **5a** (345 mg, 2.04 mmol) in $CH_2Cl_2$ (11.9 mL) was treated according to the abovementioned procedure. Purification over silica gel (10% pentane/90% $CH_2Cl_2$) afforded compound **2a** as white solid (419 mg, 71%). The NMR spectroscopic data were in agreement with those described in the literature.[9]

**2b**: Boc-protected alkyne **5b** (1.27 g, 6 mmol) in $CH_2Cl_2$ (35 mL) was treated according to the abovementioned procedure. Purification over silica gel (10% pentane/90% $CH_2Cl_2$) afforded compound **2b** as white solid (1.64 g, 82%). The NMR spectroscopic data were in agreement with those described in the literature.[10]

**2c**: Boc-protected alkyne **5c** (527 mg, 2.74 mmol) in $CH_2Cl_2$ (16.1 mL) was treated according to the abovementioned procedure. Purification over silica gel (10% pentane/90% $CH_2Cl_2$) afforded compound **2c** as white solid (599 mg, 69%). The NMR spectroscopic data were in agreement with those described in the literature.[11]

# IV. MS and HPLC data of starting and final cyclic peptides

Table S1: MS data, HRMS, retention time, and HPLC purities of starting peptides **P** (used for large scale synthesis of CP) and the MS data for the corresponding macrocyclic peptides **CP**

| Ref | Peptide | MS $[M+H]^+$ | HRMS* | rt | Purity** | MS of CP $[M+H]^+$ |
|---|---|---|---|---|---|---|
| **P0** | VGAGIGFPAYD | 1065.4 | 1065.5372 | 16.97 | 99% | 699.4 |
| **P1** | $V^{Tz}$GAGIGFPAYD | 1089.4 | 1089.5473 | 17.32 | 99% | 723.4 |
| **P2** | V$G^{Tz}$AGIGFPAYD | 1089.4 | 1089.5487 | 17.26 | 99% | 723.4 |
| **P3** | VG$A^{Tz}$GIGFPAYD | 1089.4 | 1089.5473 | 17.38 | 99% | 723.4 |
| **P4** | VGA$G^{Tz}$IGFPAYD | 1089.4 | 1089.5477 | 17.61 | 93% | 723.4 |
| **P5** | VGAG$I^{Tz}$GFPAYD | 1089.4 | 1089.5468 | 17.7 | 97% | 723.4 |
| **P6** | VGAGI$G^{Tz}$FPAYD | 1089.4 | 1111.5291 | 17.51 | 98% | 723.4 |
| **P13** | $V^{Tz}$G$A^{Tz}$GIGFPAYD | 1113.4 | 1113.5600 | 17.64 | 96% | 747.4 |
| **P135** | $V^{Tz}$G$A^{Tz}$G$I^{Tz}$GFPAYD | 1137.4 | 1137.5712 | 18.07 | 97% | 771.4 |

* Calc. for **P0** $C_{50}H_{72}N_{12}O_{14}$ $[M+H]^+$: 1065.5364; Calc. for **P1-6** $C_{51}H_{72}N_{14}O_{13}$ $[M+H]^+$: 1089.5476; $[M+Na]^+$: 1111.5295; Calc. for **P13** $C_{52}H_{72}N_{16}O_{12}$ $[M+H]^+$: 1113.5588; Calc. for **P135** $C_{53}H_{72}N_{18}O_{11}$ $[M+H]^+$: 1137.5701.** Purity assessed by analytical HPLC at 220 nm UV absorption. LCMS traces of all precursor peptides **P** can be found in section IX.

Table S2: MS data, retention time, and HPLC purities of the remaining starting peptides **P** (used for small scale macrocyclisation reactions) and the MS data for the corresponding macrocyclic peptides.

| Ref | Peptide | MS $[M+H]^+$ | rt | Purity** | MS of CP $[M+H]^+$ |
|---|---|---|---|---|---|
| **P14** | $V^{Tz}$GA$G^{Tz}$IGFPAYD | 1113.4 | 17.86 | 98% | 747.4 |
| **P15** | $V^{Tz}$GAG$I^{Tz}$GFPAYD | 1113.4 | 17.61 | 99% | 747.4 |
| **P16** | $V^{Tz}$GAGI$G^{Tz}$FPAYD | 1113.4 | 17.59 | 99% | 747.4 |
| **P24** | V$G^{Tz}$A$G^{Tz}$IGFPAYD | 1113.4 | 17.91 | 98% | 747.4 |
| **P25** | V$G^{Tz}$AG$I^{Tz}$GFPAYD | 1113.4 | 17.69 | 98% | 747.4 |
| **P26** | V$G^{Tz}$AGI$G^{Tz}$FPAYD | 1113.4 | 17.58 | 99% | 747.4 |
| **P35** | VG$A^{Tz}$G$I^{Tz}$GFPAYD | 1113.4 | 17.96 | 98% | 747.4 |
| **P36** | VG$A^{Tz}$GI$G^{Tz}$FPAYD | 1113.4 | 17.90 | 94% | 747.4 |
| **P46** | VGA$G^{Tz}$I$G^{Tz}$FPAYD | 1113.4 | 18.00 | 97% | 747.4 |
| **P246** | V$G^{Tz}$A$G^{Tz}$I$G^{Tz}$FPAYD | 1137.4 | 18.28 | 99% | 771.4 |

.** Purity assessed by analytical HPLC at 220 nm UV absorption. LCMS traces of all precursor peptides **P** can be found in section IX.

Table S3: HRMS, HPLC data, and yields of the final cyclic peptides **CP**

| | Cyclic Peptide | Ratio HPLC | Molecular formula | Expected $[M+H]^+$ | Found $[M+H]^+$ | Expected $[M+Na]^+$ | Found $[M+Na]^+$ | rt | Purity | Yield |
|---|---|---|---|---|---|---|---|---|---|---|
| **CP0** | VGAGIGFP | 100% | $C_{34}H_{50}N_8O_8$ | 699.3824 | **699.3816** | 721.3644 | **721.3629** | 20.27 | 99% | 32% |
| **CP1** | $V^{Tz}$GAGIGFP | 100% | $C_{35}H_{50}N_{10}O_7$ | 723.3937 | **-** | 745.3756 | **745.3754** | 20.45 | 99% | 43% |
| **CP2** | $VG^{Tz}$AGIGFP | 100% | $C_{35}H_{50}N_{10}O_7$ | 723.3937 | **723.3929** | 745.3756 | **745.3747** | 21.08 | 91% | 48% |
| **CP3** | $VGA^{Tz}$GIGFP | 76% | $C_{35}H_{50}N_{10}O_7$ | 723.3937 | **-** | 745.3756 | **745.3756** | 18.37 | 99% | 55% |
| | | 24% | | | **723.3942** | | **745.3757** | 20.63 | 97% | |
| **CP4** | $VGAG^{Tz}$IGFP | 100% | $C_{35}H_{50}N_{10}O_7$ | 723.3937 | **723.3927** | 745.3756 | **745.3744** | 20.45 | 98% | 34% |
| **CP5** | $VGAGI^{Tz}$GFP | 76% | $C_{35}H_{50}N_{10}O_7$ | 723.3937 | **723.3941** | 745.3756 | **745.3756** | 20.54 | 93% | 58% |
| **CP6** | $VGAGIG^{Tz}$FP | 100% | $C_{35}H_{50}N_{10}O_7$ | 723.3937 | **723.3927** | 745.3756 | **745.3746** | 20.63 | 98% | 40% |
| **CP13** | $V^{Tz}GA^{Tz}$GIGFP | 20% | $C_{36}H_{50}N_{12}O_6$ | 747.4049 | **747.4054** | 769.3868 | **769.3873** | 20.11 | 98% | 54% |
| | | 80% | | | **747.4046** | | **769.3863** | 20.19 | 98% | |
| **CP135** | $V^{Tz}GA^{Tz}GI^{Tz}$GFP | 15% | $C_{37}H_{50}N_{14}O_5$ | 771.4161 | **771.4162** | 793.3981 | **793.3980** | 21.32 | 91% | 39% |
| | | 85% | | | **771.4159** | | **793.3979** | 21.54 | 97% | |

# V. NMR data of final cyclic peptides

Copies of the proton NMR spectra for each compound depicting the different species by color code as well as copies of the HSQC spectra can be found in section V. Copies of the exchange spectroscopy (EXSY) spectra can be found in section VI.

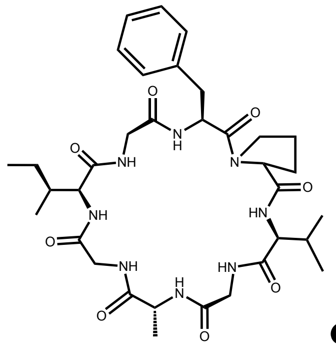

**CP0**; Cyclo(-VG$_a$AG$_b$IG$_c$FP-) **3:1** (conformers)

$^1$H NMR: (700 MHz, DMSO); HSQC and HMBC analysis (700 MHz, DMSO)*

| Amino acid | Atom | $^1$H chemical shift | $^{13}$C chemical shift |
|---|---|---|---|
| Val | NH | 8.10, d, *J* = 8.5 | - |
| | α CH | 3.76, t, *J* = 8.7 | 61.4 |
| | β CH | 2.02-1.96 m | 29.3 |
| | γ $CH_3$ | 0.82-0.78, m | 15.5 |
| | | | 19.6 |
| | CO | | 171.1 |
| Gly (a) | NH | 7.25-7.21, m | - |
| | α $CH_2$ | 3.92-3.86, m;<br>3.68-3.61, m | 41.7 |
| | CO | - | 169.9 |
| Ala | NH | 8.56, d, *J* = 6.1 | - |
| | α CH | 4.12-4.07, m | 49.3 |
| | β $CH_3$ | 1.27, d, *J* = 7.2 | 17.5 |
| | CO | - | 173.3 |
| Gly (b) | NH | 8.47, t, *J* = 5.88 | - |
| | α $CH_2$ | 3.97-3.92, m;<br>3.51-3.47, m | 43.2 |
| | CO | - | 170.4 |
| Ile | NH | 7.85, d, *J* = 7.7 | - |
| | α CH | 4.02, d, *J* = 7.2 | 58.8 |
| | β CH | 1.81-1.75, m | 36.2 |
| | γ $CH_2$ | 1.42-1.35, m; 1.20-1.15, m | 25.0 |
| | γ $CH_3$ | 0.85-0.83, m | 15.7 |
| | δ $CH_3$ | 0.82-0.79, m | 11.2 |
| | CO | - | 170.8 |
| Gly (c) | NH | 4.32, dd, *J* = 4.9, *J* = 6.8 | - |
| | α $CH_2$ | 3.92-3.86, m;<br>3.68-3.61, m | 41.7 |
| | CO | | 170.7 |

| | | | |
|---|---|---|---|
| Phe | NH | 8.41, d, *J* = 3.5 | - |
| | α CH | 4.50, qt, *J* = 5.1 | 53.8 |
| | β $CH_2$ | 3.07, dd, *J* = 5.7, *J* = 12.9;<br>2.86, dd, *J* = 10.4, *J* = 12.8 | 37.5 |
| | Ar CH | 7.33, t, *J* = 7.4 | 128.9 |
| | | 7.28, t, *J* = 7.3 | 127.5 |
| | | 7.25-7.21, m | 129.9 |
| | Ar C | | 136.4 |
| | CO | - | 170.4 |
| Pro | α CH | 3.61-3.58, m | 60.7 |
| | β $CH_2$ | 1.89-1.82, m; 0.87-0.84, m | 30.2 |
| | γ $CH_2$ | 1.63-1.56, m; 1.34-1.28, m | 21.7 |
| | δ $CH_2$ | 3.33-3.25, m; 3.23-3.18, m | 46.2 |
| | CO | - | 170.4 |

*NMR data of the major conformer

$^{1}$H NMR: (700 MHz, DMSO); HSQC and HMBC analysis (700 MHz, DMSO)*

| **Amino acid*** | **Atom** | **$^{1}$H chemical shift** | **$^{13}$C chemical shift** |
|---|---|---|---|
| Val | NH | 7.68, d, *J* = 7.5 | - |
| | α CH | 4.12-4.07, m | 58.3 |
| | β CH | 2.09-2.03 m | 30.7 |
| | γ $CH_3$ | 0.85-0.83, m; | 19.6 |
| | | | 18.2 |
| | CO | | 171.5 |
| Gly (a) | NH | 8.24, t, *J* = 5.5 | - |
| | α $CH_2$ | 3.82, t, *J* = 5.3; 3.58-3.53, m | 43.0 |
| | CO | - | 169.2 |
| Ala | NH | 8.03, d, *J* = 7.5 | - |
| | α CH | 4.18, qt, *J* = 7.2 | 48.9 |
| | β $CH_3$ | 1.22, d, *J* = 7.1 | 17.5 |
| | CO | - | 172.1 |
| Gly (b) | NH | 7.79, t, *J* = 5.6 | - |
| | α $CH_2$ | 3.80, t, *J* = 5.7; 3.71-3.68, m | 42.2 |
| | CO | - | 169.8 |
| Ile | NH | 8.18, d, *J* = 7.0 | - |
| | α CH | 3.97-3.95, m | 58.1 |
| | β CH | 1.76-1.72, m | 35.3 |
| | γ $CH_2$ | 1.53-1.48, m; 1.12-1.05, m | 24.9 |
| | γ $CH_3$ | 0.83-0.80, m; | 15.5 |
| | δ $CH_3$ | 0.83-0.81, m | 11.2 |
| | CO | - | ND |
| Gly (c) | NH | 8.63, t, *J* = 5.6 | - |
| | α $CH_2$ | 3.97-3.92, m; 3.29-3.25, m | 42.8 |
| | CO | | 172.3 |
| Phe | NH | 7.61, d, *J* = 8.4 | - |
| | α CH | 4.71, dt, *J* = 4.4, *J* = 9.0 | 52.5 |
| | β $CH_2$ | 2.95, dd, *J* = 4.2, *J* = 14.2<br>2.78, dd, *J* = 9.6, *J* = 14.2 | 37.6 |

| | | | |
|---|---|---|---|
| | Ar CH | 7.25-7.21, 7.20-7.17 m | 128.6, 129.4, 126.5 |
| | Ar C | - | 137.8 |
| | CO | - | 171.2 |
| Pro | α CH | 4.34, dd, *J* = 5.0, *J* = 7.7 | 61.2 |
| | β $CH_2$ | 1.96-1.93, m; 1.25-1.23, m | 28.0 |
| | γ $CH_2$ | 1.89-1.82, m; 1.48-1.43, m | 25.0 |
| | δ $CH_2$ | 3.58-3.53, m | 47.4 |
| | CO | - | 171.4 |

*NMR data of the minor conformer

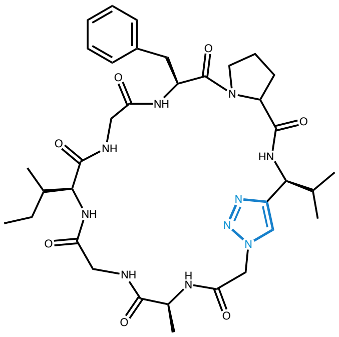

**CP1**; Cyclo(-VTzGaAG$_b$IG$_c$FP-) **4:1**

$^1$H NMR: (700 MHz, DMSO); HSQC and HMBC analysis (700 MHz, DMSO)*

| Amino acid* | Atome | $^1$H chemical shift | $^{13}$C chemical shift |
|---|---|---|---|
| Val | NH | 8.51, d, *J* = 9.3 | - |
| | α CH | 4.63, t, *J* = 9.6 | 53.0 |
| | β CH | 2.02-1.98 m | 33.0 |
| | γ $CH_3$ | 0.65, d, *J* = 6.7; | 20.3 |
| | | 0.82, d, *J* = 6.6 | 19.8 |
| | Tz CH | 7.75, s | 124.4 |
| | Tz C | | 148.9 |
| Gly (a) | α $CH_2$ | 5.16, s | 51.6 |
| | CO | - | 167.2 |
| Ala | NH | 9.10, d, *J* = 6.1 | - |
| | α CH | 3.93, q, *J* = 6.9 | 50.2 |
| | β $CH_3$ | 1.29, d, *J* = 7.0 | 16.6 |
| | CO | - | 171.9 |
| Gly (b) | NH | 8.39, dd, *J* = 5.8, *J* = 6.4 | - |
| | α $CH_2$ | 4.01-3.98, m; | 42.8 |
| | | 3.40, dd, *J* = 5.0, *J* = 16.3 | |
| | CO | - | 169.3 |
| Ile | NH | 7.31 | - |
| | α CH | 4.01-3.98, m | 57.7 |
| | β CH | 1.58-1.54, m | 35.8 |
| | γ $CH_2$ | 1.35-1.31, m; 1.02-0.98, m | 25.2 |
| | γ $CH_3$ | 0.88, d, *J* = 6.8; | 15.9 |
| | δ $CH_3$ | 0.69, t, *J* = 7.4 | 10.8 |
| | CO | - | 171.6 |
| Gly (c) | NH | 7.95-7.92, m | - |
| | α $CH_2$ | 3.83, dd, *J* = 7.4, *J* = 16.7; | 41.9 |
| | | 3.70, dd, *J* = 4.2, *J* = 16.8 | |

| | | | |
|---|---|---|---|
| | CO | | 169.8 |
| Phe | NH | 8.49, d, *J* = 3.6 | - |
| | α CH | 4.22-4.19, m; | 54.0 |
| | β $CH_2$ | 2.98, dd, *J* = 6.1, *J* = 13.2;<br>2.83, dd, *J* = 10.0, *J* = 13.2 | 37.5 |
| | Ar CH | 7.32-7.18, m | 129.9, 129.5, 129.0 |
| | Ar C | - | 136.4 |
| | CO | - | ND |
| Pro | α CH | 3.52-3.51, m | 60.9 |
| | β $CH_2$ | 2.02-1.97, m; 0.77-0.71, m | 29.8 |
| | γ $CH_2$ | 1.64-1.60, m; 1.32-1.27, m | 21.9 |
| | δ $CH_2$ | 3.38-3.31, m;<br>3.17, t, *J* = 10.0 | 46.3 |
| | CO | - | 169.3 |

*NMR data of the major conformer

$^1$H NMR: (700 MHz, DMSO); HSQC and HMBC analysis (700 MHz, DMSO)*

| **Amino acid*** | **Atome** | **$^1$H chemical shift** | **$^{13}$C chemical shift** |
|---|---|---|---|
| Val | NH | 8.89, d, *J* = 7.6 | - |
| | α CH | 4.79, dd, *J* = 7.2, *J* = 8.8 | 51.6 |
| | β CH | 2.15-2.06 m | 32.4 |
| | γ $CH_3$ | 0.86-0.83, m;<br>0.81-0.77, m; | 19.3<br>15.6 |
| | Tz CH | 7.65, s | 123.5 |
| | Tz C | | 147.7 |
| Gly (a) | α $CH_2$ | 5.06, dd, *J* = 15.9, *J* = 52.9 | 51.9 |
| | CO | - | 165.3 |
| Ala | NH | 8.64, d, *J* = 7.7 | - |
| | α CH | 4.40, t, *J* = 7.0 | 48.8 |
| | β $CH_3$ | 1.21, d, *J* = 6.8 | 18.4 |
| | CO | - | 172.1 |
| Gly (b) | NH | 8.56-8.55, m | - |
| | α $CH_2$ | 4.02-3.98, m; 3.86-3.85, m | 41.7 |
| | CO | - | ND |
| Ile | NH | 7.44, d, *J* = 8.2 | - |
| | α CH | 2 t, *J* = 7.9 | 57.5 |
| | β CH | 1.66-1.64, m | 35.9 |
| | γ $CH_2$ | 1.48-1.43, m; 1.11-1.05, m | 25.1 |
| | $CH_3$ | 0.86-0.83, m; | 14.5 |
| | $CH_3$ | 0.81-0.77, m | 11.0 |
| | CO | - | ND |
| Gly (c) | NH | 8.05-8.03, m | - |
| | α $CH_2$ | 3.55-3.53,m;<br>3.24, dd, *J* = 3.8, *J* = 16.3 | 42.4 |
| | CO | | ND |
| Phe | NH | 7.93-7.92, m | - |
| | α CH | 4.67-4.66, m; | 52.4 |
| | β $CH_2$ | 2.78-2.77, m; 2.99-2.97, m | 36.5 |
| | Ar CH | 7.31-7.15, m | 129.3, 128.5, 126.7 |

| | | | |
|---|---|---|---|
| | Ar C | - | 136.4 |
| | CO | - | ND |
| Pro | α CH | 4.32, dd, *J* = 4.0, *J* = 7.8 | 50.9 |
| | β $CH_2$ | 2.03-2.02, m; 1.48-1.43, m | 27.0 |
| | γ $CH_2$ | 1.94-1.91, m | 28.9 |
| | δ $CH_2$ | 3.62-3.53, m | 47.5 |
| | CO | - | ND |

*NMR data of the minor conformer

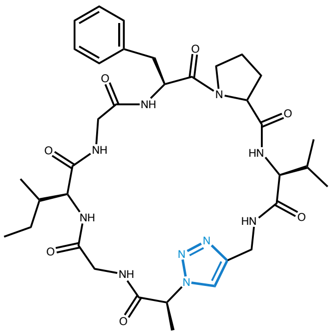

**CP2**; Cyclo(-VG$_a$TzAG$_b$IG$_c$FP-) **1:1**

$^1$H NMR: (700 MHz, DMSO); HSQC and HMBC analysis (700 MHz, DMSO)*

| Amino acid* | Atome | $^1$H chemical shift | $^{13}$C chemical shift |
|---|---|---|---|
| Val | NH | 7.75, d, *J* = 8.6 | - |
| | α CH | 3.80-3.76, m | 61.6 |
| | β CH | 1.97-1.92 m | 29.6 |
| | γ $CH_3$ | 0.86-0.79, m | 19.8/18.7 |
| | CO | | 170.9 |
| Gly (a) | NH | 7.43, d, *J* = 6.7 | - |
| | α $CH_2$ | 4.53, dd, *J* = 7.7, *J* = 14.6; 4.06-3.98, m | 34.2 |
| | Tz CH | 8.12, s | 122.3 |
| | Tz C | | 144.1 |
| Ala | α CH | 5.63, q, *J* = 6.5 | 57.9 |
| | β $CH_3$ | 1.67, d, *J* = 6.8 | 19.4 |
| | CO | - | 170.9 |
| Gly (b) | NH | 9.42-9.38, m | - |
| | α $CH_2$ | 4.08-4.06, m; 3.65, dd, *J* = 6.1, *J* = 14.2 | 43.7 |
| | CO | - | 171.9 |
| Ile | NH | 8.61, d, *J* = 5.9 | - |
| | α CH | 4.06-3.98, m | 59.3 |
| | β CH | 1.92-1.88, m | 35.8 |
| | γ $CH_2$ | 1.78-1.74, m; 1.35-1.28, m | 24.5 |
| | γ $CH_3$ | 0.92, d, *J* = 6.8; | 15.9 |
| | δ $CH_3$ | 0.86-0.79, m | 12.2 |
| | CO | - | 170.5 |
| Gly (c) | NH | 7.87-7.85, m | - |
| | α $CH_2$ | 3.76-3.71, m; 2.50 | 42.4 |
| | CO | | 168.8 |
| Phe | NH | 7.98-7.97, m | - |

| | | | |
|---|---|---|---|
| | α CH | 4.68-4.63, m; | 52.6 |
| | β $CH_2$ | 2.87-2.82, m;<br>2.70-2.65, m | 36.6 |
| | Ar CH | 7.22-7.13, m | 129.7, 128.5, 126.3 |
| | Ar C | | 138.7 |
| | CO | - | ND |
| Pro | α CH | 3.43, d, *J* = 7.6 | 60.5 |
| | β $CH_2$ | 1.78-1.74, m; 0.72-0.64, m | 30.1 |
| | γ $CH_2$ | 1.59-1.53, m; 1.35-1.28, m | 21.7 |
| | δ $CH_2$ | 3.30-3.25, m; 3.22-3.15, m | 46.1 |
| | CO | - | 172.8 |

*NMR data of the first conformer

$^1$H NMR: (700 MHz, DMSO); HSQC and HMBC analysis (700 MHz, DMSO)*

| **Amino acid*** | **Atome** | **$^1$H chemical shift** | **$^{13}$C chemical shift** |
|---|---|---|---|
| Val | NH | 7.66, d, *J* = 8.8 | - |
| | α CH | 4.06-3.98, m | 59.3 |
| | β CH | 2.08-2.03, m | 30.1 |
| | γ $CH_3$ | 0.86-0.79, m | 19.8/14.4 |
| | CO | | 170.9 |
| Gly (a) | NH | 8.19-8.15, m | |
| | α $CH_2$ | 4.48, dd, *J* = 6.3, *J* = 15.5;<br>4.15, dd, *J* = 4.5, *J* = 15.0 | 34.9 |
| | Tz CH | 7.98, s | 122.7 |
| | Tz C | | 145.3 |
| Ala | α CH | 5.43, q, *J* = 6.8 | 58.4 |
| | β $CH_3$ | 1.62, d, *J* = 6.8 | 18.9 |
| | CO | - | 169.0 |
| Gly (b) | NH | 8.41-8.45, m | - |
| | α $CH_2$ | 4.11, dd, *J* = 7.5, *J* = 16.9;<br>3.58, d, *J* = 15.6 | 41.7 |
| | CO | - | 169.3 |
| Ile | NH | 8.39, d, *J* = 7.1 | - |
| | α CH | 4.06-3.98, m | 58.1 |
| | β CH | 1.74-1.69, m | 36.1 |
| | γ $CH_2$ | 1.50-1.42, m; 1.13-1.09, m | 25.1 |
| | γ $CH_3$ | 0.86-0.79, m | 15.6 |
| | δ $CH_3$ | 0.86-0.79, m | 11.2 |
| | CO | - | 172.1 |
| Gly (c) | NH | 8.63-8.66, m | - |
| | α $CH_2$ | 4.06-3.98, m; 3.25-3.22, m | 42.4 |
| | CO | - | 170.7 |
| Phe | NH | 7.38-7.35, m | - |
| | α CH | 4.08-4.06, m | 53.5 |
| | β $CH_2$ | 2.93, dd, *J* = 3.8, *J* = 12.5;<br>2.87-2.82, m | 37.9 |
| | Ar CH | 7.39-7.34, m; 7.33-7.29, m | 130.0, 129.2, 127.6 |
| | Ar C | | 136.6 |

| | | | |
|---|---|---|---|
| | CO | - | 170.5 |
| Pro | α CH | 4.31, d, *J* = 8.4 | 60.9 |
| | β $CH_2$ | 1.88-1.82, m; 1.73-1.69, m | 29.3 |
| | γ $CH_2$ | ND | ND |
| | δ $CH_2$ | 3.49-3.47, m; 2.50 | 47.2 |
| | CO | - | 171.6 |

*NMR data of the second conformer

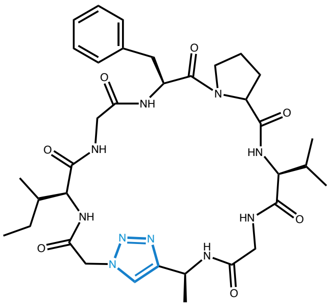


**CP3**; Cyclo(-VGaATzG$_b$IG$_c$FP-) **1:0.9**

$^{1}$H NMR: (700 MHz, DMSO); HSQC and HMBC analysis (700 MHz, DMSO)*

| Amino acid* | Atome | $^1$H chemical shift | $^{13}$C chemical shift |
|---|---|---|---|
| Val | NH | 7.89-7.83, m | - |
| | α CH | 3.82-3.79, m | 60.0 |
| | β CH | 1.95-1.92 m | 29.1/29.6 |
| | γ $CH_3$ | 0.96, d, *J* = 6.6; | 19.6 |
| | | 0.90-0.86, m | 19.6 |
| | CO | | 171.9 |
| Gly (a) | NH | 8.46-8.41, m | - |
| | α $CH_2$ | 4.09, dd, *J* = 8.4, *J* = 16.8; 3.44-3.38, m | 42.4 |
| | CO | | 168.6 |
| Ala | NH | 8.34, d, *J* = 9.3 | - |
| | α CH | 5.12-5.07, m | 41.4 |
| | β $CH_3$ | 1.37, d, *J* = 6.9 | 21.5 |
| | Tz CH | 7.69, s | 123.2 |
| | Tz C | - | 150.3 |
| Gly (b) | α $CH_2$ | 5.12-5.07, m | 51.4 |
| | CO | - | 166.9 |
| Ile | NH | 8.79-8.76, m | - |
| | α CH | 3.82-3.79, m | 60.0 |
| | β CH | 1.77-1.73, m | 35.2 |
| | γ $CH_2$ | 1.65-1.58, m; 1.24-1.21, m | 25.4 |
| | γ $CH_3$ | 0.91-0.86, m | 15.6 |
| | δ $CH_3$ | 0.90-0.84, m | 11.0 |
| | CO | - | 171.9 |
| Gly (c) | NH | 8.79-8.76, m | - |
| | α $CH_2$ | 3.92-3.87, m; 3.21, dd, *J* = 4.5, *J* = 16.9 | 42.4 |
| | CO | | 168.4 |
| Phe | NH | 7.65, d, *J* = 9.2 | - |

| | | | |
|---|---|---|---|
| | α CH | 4.55-4.51, m | 52.8 |
| | β $CH_2$ | 2.70-2.66, m;<br>2.13-2.07, m | 36.6 |
| | Ar CH | 7.22-7.13, m; 7.04-7.00, m | 129.5, 128.3, 126.3 |
| | Ar C | | 139.0 |
| | CO | - | ND |
| Pro | α CH | 4.30, t, *J* = 7.4 | 60.2 |
| | β $CH_2$ | 2.19-2.13, m; 1.75-1.68, m | 29.4 |
| | γ $CH_2$ | 1.92-1.89, m; 1.87-1.83, m | 25.6 |
| | δ $CH_2$ | 3.66-3.59, m; 3.50-3.46, m | 47.7 |
| | CO | - | 168.6 |

*NMR data of the major conformer

$^1$H NMR: (700 MHz, DMSO); HSQC and HMBC analysis (700 MHz, DMSO)*

| Amino acid* | Atome | $^1$H chemical shift | $^{13}$C chemical shift |
|---|---|---|---|
| Val | NH | 8.65-8.61, m | - |
| | α CH | 3.70, dd, *J* = 6.3, *J* = 9.0 | 61.6 |
| | β CH | 1.95-1.92 m | 29.1/29.6 |
| | γ $CH_3$ | 0.94, d, *J* = 6.6;<br>0.87-0.85, m | 20.0<br>19.5 |
| | CO | | 172.3 |
| Gly (a) | NH | 8.13-8.07, m | - |
| | α $CH_2$ | 3.77, dd, *J* = 6.0, *J* = 17.3;<br>3.66-3.59, m | 42.0 |
| | CO | | 168.7 |
| Ala | NH | 7.96, d, *J* = 8.4 | - |
| | α CH | 5.25-5.17, m | 42.6 |
| | β $CH_3$ | 1.26, d, *J* = 7.0 | 23.3 |
| | Tz CH | 8.01, s | 122.3 |
| | Tz C | - | 150.7 |
| Gly (b) | α $CH_2$ | 5.25-5.17, m;<br>5.15-5.12, m | 51.9 |
| | CO | - | 166.7 |
| Ile | NH | 8.79-8.76, m | - |
| | α CH | 3.94-3.90, m | 59.3 |
| | β CH | 1.81-1.77, m | 34.9 |
| | γ $CH_2$ | 1.57-1.52, m; 1.21-1.17, m | 25.0 |
| | s | 0.89-0.83, m | 15.6 |
| | δ $CH_3$ | 0.90-0.84, m | 11.0 |
| | CO | - | 171.6 |
| Gly (c) | NH | 8.65-8.61, m | - |
| | α $CH_2$ | 3.87-3.83, m;<br>3.54, dd, *J* = 5.8, *J* = 16.9 | 43.1 |
| | CO | | 169.8 |
| Phe | NH | 7.45, d, *J* = 5.8 | - |
| | α CH | 4.55-4.51, m | 52.8 |
| | β $CH_2$ | 2.75, dd, *J* = 8.3, *J* = 12.8;<br>2.69-2.62, m | 38.2 |
| | Ar CH | 7.30-7.20, m; 7.10-7.06, m | 130.0, 128.8, 127.2 |

| | | | |
|---|---|---|---|
| | Ar C | | 136.40 |
| | CO | - | 169.8 |
| Pro | α CH | 3.97-3.94, m | 59.5 |
| | β $CH_2$ | 1.80-1.76, m; 1.53-1.48, m | 31.0 |
| | γ $CH_2$ | 1.65-1.58, m; 1.51-1.46, m | 22.2 |
| | δ $CH_2$ | 3.29-3.25, m; 3.17-3.12, m | 46.8 |
| | CO | - | 172.6 |

*NMR data of the minor conformer

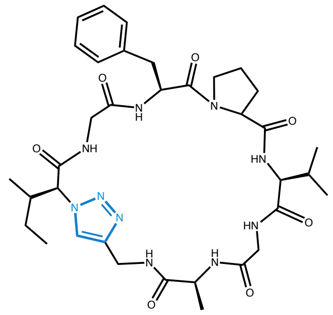

**CP4**; Cyclo(-VGaAG$_b$TzIG$_c$FP-) **1:1**

$^1$H NMR: (700 MHz, DMSO); HSQC and HMBC analysis (700 MHz, DMSO)*

| Amino acid* | Atome | $^1$H chemical shift | $^{13}$C chemical shift |
|---|---|---|---|
| Val | NH | 7.88-7.85, m | - |
| | α CH | 4.08, dd, $J$ = 6.4, $J$ = 8.3 | 58.4 |
| | β CH | 2.04-1.95, m | 30.3 |
| | γ $CH_3$ | 0.82, d, $J$ = 6.7; | 19.8 |
| | | 0.81, d, $J$ = 6.7 | 18.4 |
| | CO | | 171.4 |
| Gly (a) | NH | 8.16-8.14, m | - |
| | α $CH_2$ | 3.93, dd, $J$ = 6.4, $J$ = 16.2; | 42.6 |
| | | 3.40-3.38, m | |
| | CO | | 168.4 |
| Ala | NH | 7.98-7.96, m | - |
| | α CH | 4.32, quintet, $J$ = 7.2 | 48.4 |
| | β $CH_3$ | 1.20, d, $J$ = 7.1 | 19.1 |
| | CO | - | 172.4 |
| Gly (b) | NH | 8.53, dd, $J$ = 5.0, $J$ = 7.0 | - |
| | α $CH_2$ | 4.59-4.58, m; 4.05-4.00, m | 34.4 |
| | Tz CH | 8.02, s | 121.4 |
| | Tz C | - | 145.3 |
| Ile | α CH | 5.19, d, $J$ = 10.1 | 66.8 |
| | β CH | 2.16-2.09, m | 37.7 |
| | γ $CH_2$ | 1.01-0.95, m; 0.86-0.83, m | 24.7 |
| | γ $CH_3$ | 0.87, d, $J$ = 6.7 | 15.4 |
| | δ $CH_3$ | 0.78-0.73, m | 10.5 |
| | CO | - | 168.1 |
| Gly (c) | NH | 8.99, dd, $J$ = 3.5, $J$ = 7.9 | - |
| | α $CH_2$ | 4.22, dd, $J$ = 7.9, $J$ = 15.5; | 42.6 |
| | | 3.25-3.22, m | |
| | CO | | 168.8 |

| | | | |
|---|---|---|---|
| Phe | NH | 8.44, d, *J* = 7.8 | - |
| | α CH | 4.50-4.46, m | 53.7 |
| | β $CH_2$ | 2.99, dd, *J* = 4.1, *J* = 14.0; 2.78-2.74, m | 37.0 |
| | Ar CH | 7.31-7.29, m; 7.28-7.24, m; 7.22-7.19, m | 129.7, 128.6, 127.0 137.6 |
| | Ar C | | |
| | CO | - | 170.0 |
| Pro | α CH | 4.46-4.44, m | 60.5 |
| | β $CH_2$ | 2.04-1.95, m; 1.80-1.72, m | 27.9 |
| | γ $CH_2$ | 1.80-1.72, m | 25.1 |
| | δ $CH_2$ | 3.53-3.50, m; 3.43-3.40, m | 47.2 |
| | CO | - | 171.2 |

*NMR data of the first conformer

$^1$H NMR: (700 MHz, DMSO); HSQC and HMBC analysis (700 MHz, DMSO)*

| Amino acid* | Atome | $^1$H chemical shift | $^{13}$C chemical shift |
|---|---|---|---|
| Val | NH | 8.01, d, *J* = 6.5 | - |
| | α CH | 3.87, t, *J* = 6.5 | 59.6 |
| | β CH | 1.92-1.85, m | 30.3 |
| | γ $CH_3$ | 0.78-0.73, m; 0.72, d, *J* = 6.8 | 19.4 18.9 |
| | CO | | 171.4 |
| Gly (a) | NH | 8.00-7.98, m | - |
| | α $CH_2$ | 3.67, ddd, *J* = 5.4, *J* = 16.4, *J* = 50.3 | 42.8 |
| | CO | | 169.3 |
| Ala | NH | 7.87-7.85, m | - |
| | α CH | 4.17-4.13, m | 48.9 |
| | β $CH_3$ | 1.20, d, *J* = 7.1 | 17.7 |
| | CO | - | 172.1 |
| Gly (b) | NH | 8.20, dd, *J* = 5.4, *J* = 6.7 | - |
| | α $CH_2$ | 4.57-4.53, m; 4.01-3.99, m | 34.5 |
| | Tz CH | 7.85, s | 121.8 |
| | Tz C | - | 145.6 |
| Ile | α CH | 5.07, d, *J* = 10.1 | 67.4 |
| | β CH | 2.16-2.09, m | 37.7 |
| | γ $CH_2$ | 1.01-0.95, m; 0.86-0.83, m | 24.7 |
| | γ $CH_3$ | 0.87, d, *J* = 6.7 | 15.4 |
| | δ $CH_3$ | 0.78-0.73, m | 10.5 |
| | CO | - | 167.7 |
| Gly (c) | NH | 8.62, dd, *J* = 4.1, *J* = 7.2 | - |
| | α $CH_2$ | 4.05-4.03, m; 3.40-3.37, m | 41.9 |
| | CO | | 167.9 |
| Phe | NH | 8.15-8.12, m | - |
| | α CH | 4.57-4.53, m | 52.3 |
| | β $CH_2$ | 2.85, dd, *J* = 7.3, *J* = 13.3; 2.80-2.77, m | 39.1 |

| | | | |
|---|---|---|---|
| | Ar CH | 7.28-7.24, m; 7.22-7.19, m | 129.7, 128.3, 126.3 |
| | | 7.19-7.18, m | 137.6 |
| | Ar C | | |
| | CO | - | 169.8 |
| Pro | α CH | 4.17-4.13, m | 59.5 |
| | β $CH_2$ | 1.92-1.85, m; 1.66-1.57, m | 31.6 |
| | γ $CH_2$ | 1.66-1.57, m | 22.1 |
| | δ $CH_2$ | 3.36-3.32, m; 3.27-3.25, m | 46.8 |
| | CO | - | 171.9 |

*NMR data of the second conformer

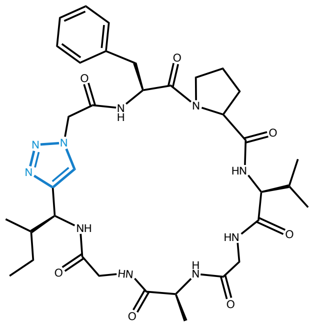

**CP5**; Cyclo(-VGaAG$_b$ITzG$_c$FP-) **1:0.6**

$^1$H NMR: (700 MHz, DMSO); HSQC and HMBC analysis (700 MHz, DMSO)*

| **Amino acid*** | **Atome** | **$^1$H chemical shift** | **$^{13}$C chemical shift** |
|---|---|---|---|
| Val | NH | 7.86, d, $J$ = 8.8 | - |
| | α CH | 4.13, dd, $J$ = 5.5, $J$ = 8.7 | 58.1 |
| | β CH | 2.15-2.09, m | 29.7 |
| | γ $CH_3$ | 0.67, t, $J$ = 7.1; | 19.5 |
| | | 0.44, d, $J$ = 6.9 | 17.3 |
| | CO | | 172.4 |
| Gly (a) | NH | 8.05, t, $J$ = 4.7 | - |
| | α $CH_2$ | 3.79-3.74, m; | 44.4 |
| | | 3.64, dd, $J$ = 5.2, $J$ = 14.8 | |
| | CO | | 171.5 |
| Ala | NH | 8.76, d, $J$ = 5.0 | - |
| | α CH | 4.06-4.00, m | 50.6 |
| | β $CH_3$ | 1.29, d, $J$ = 7.4 | 16.7 |
| | CO | - | 172.9 |
| Gly (b) | NH | 8.43, t, $J$ = 6.3 | - |
| | α $CH_2$ | 3.82, dd, $J$ = 6.8, $J$ = 16.9; | 43.1 |
| | | 3.44-3.40, m | |
| | CO | - | 168.3 |
| Ile | NH | 7.30-7.27, m | - |
| | α CH | 5.15-5.09, m | 49.2 |
| | β CH | 1.67-1.64, m | 40.4 |
| | γ $CH_2$ | 1.35-2.30, m; 1.00-0.96, m | 25.6 |
| | γ $CH_3$ | 0.77, d, $J$ = 6.8 | 14.5 |
| | δ $CH_3$ | 0.84, t, $J$ = 7.4 | 11.8 |
| | Tz CH | 8.18, s | 124.5 |
| | Tz C | - | 147.8 |

| | | | |
|---|---|---|---|
| Gly (c) | α $CH_2$ | 5.15-5.09, m | 51.3 |
| | CO | | 166.2 |
| Phe | NH | 9.04-9.00, m | - |
| | α CH | 4.70, q, *J* = 7.4 | 52.7 |
| | β $CH_2$ | 2.96-2.90, m | 38.0 |
| | Ar CH | 7.36-7.21, m | 128.9, 127.4, 129.8 |
| | Ar C | | 136.4 |
| | CO | - | 170.7 |
| Pro | α CH | 3.90-3.86, m | 60.5 |
| | β $CH_2$ | 1.91-1.84, m; 1.68-1.58, m | 30.2 |
| | γ $CH_2$ | 1.63-1.58, m; 1.41-1.36, m | 21.9 |
| | δ $CH_2$ | 3.34-3.22, m | 46.1 |
| | CO | - | 171.2 |

*NMR data of the major species

$^1$H NMR: (700 MHz, DMSO); HSQC and HMBC analysis (700 MHz, DMSO)*

| **Amino acid*** | **Atome** | **$^1$H chemical shift** | **$^{13}$C chemical shift** |
|---|---|---|---|
| Val | NH | 7.94, d, *J* = 7.7 | - |
| | α CH | 3.79-3.74, m | 60.1 |
| | β CH | 1.84-1.78, m | 29.5 |
| | γ $CH_3$ | 0.75, d, *J* = 6.8; | 19.8 |
| | | 0.67, t, *J* = 7.1; | 19.5 |
| | CO | | 171.5 |
| Gly (a) | NH | 7.58, t, *J* = 5.7 | - |
| | α $CH_2$ | 3.90-3.86, m; | 42.6 |
| | | 3.59, dd, *J* = 5.0, *J* = 16.2 | |
| | CO | | 170.3 |
| Ala | NH | 8.50, d, *J* = 6.0 | - |
| | α CH | 3.95-3.90, m | 49.8 |
| | β $CH_3$ | 1.25, d, *J* = 7.0 | 16.9 |
| | CO | - | 172.4 |
| Gly (b) | NH | 8.36, dd, *J* = 5.7, *J* = 6.7 | - |
| | α $CH_2$ | 3.98-3.92, m; 3.38-3.33, m | 43.0 |
| | CO | - | 168.5 |
| Ile | NH | 7.63, d, *J* = 9.6 | - |
| | α CH | 4.88, dd, *J* = 7.7, *J* = 9.3 | 50.1 |
| | β CH | 1.74-1.70, m | 39.3 |
| | γ $CH_2$ | 1.44-1.41, m; 1.07-1.02, m | 24.9 |
| | γ $CH_3$ | 0.71, d, *J* = 6.7 | 15.7 |
| | δ $CH_3$ | 0.80, t, *J* = 7.5 | 11.6 |
| | Tz CH | 7.98, s | 124.1 |
| | Tz C | - | 148.6 |
| Gly (c) | α $CH_2$ | 5.28, d; 5.02, d, *J* = 16.8 | 51.3 |
| | CO | | 165.6 |
| Phe | NH | 9.04-9.00, m | - |
| | α CH | 4.54, q, *J* = 7.2 | 53.3 |
| | β $CH_2$ | 2.96-2.90, m | 38.4 |
| | Ar CH | 7.36-7.21, m | 128.9, 127.4, 129.8 |

| | | | |
|---|---|---|---|
| | Ar C | | 136.4 |
| | CO | - | 170.1 |
| Pro | α CH | 3.90-3.86, m | 61.0 |
| | β $CH_2$ | 1.91-1.84, m; 1.68-1.58, m | 30.4 |
| | γ $CH_2$ | 1.63-1.58, m; 1.51-1.42, m | 24.9 |
| | δ $CH_2$ | 3.34-3.22, m | 46.1 |
| | CO | - | 170.9 |

*NMR data of the minor species

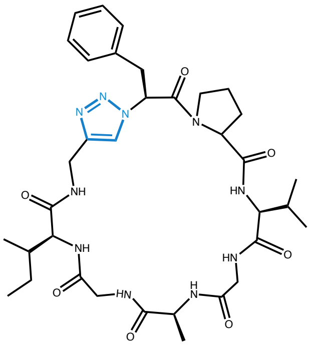

**CP6**; Cyclo(-VGaAG$_b$IG$_c$TzFP-) **2:1**

$^1$H NMR: (700 MHz, DMSO); HSQC and HMBC analysis (700 MHz, DMSO)*

| Amino acid* | Atome | $^1$H chemical shift | $^{13}$C chemical shift |
|---|---|---|---|
| Val | NH | 7.80-7.78, m | - |
| | α CH | 4.20-4.17, m | 58.1 |
| | β CH | 2.06-2.00, m | 31.4 |
| | γ $CH_3$ | 0.88-0.85, m | 19.8 |
| | | 0.87-0.84, m | 18.6 |
| | CO | | 171.3 |
| Gly (a) | NH | 8.51, t, $J$ = 5.7 | - |
| | α $CH_2$ | 3.93, d, $J$ = 15.5;<br>3.50-3.49, m | 43.3 |
| | CO | | 168.9 |
| Ala | NH | 8.07, d, $J$ = 7.8 | - |
| | α CH | 4.32-4.25, m | 48.6 |
| | β $CH_3$ | 1.24, d, $J$ = 7.1 | 18.4 |
| | CO | - | 172.5 |
| Gly (b) | NH | 8.12-8.10, m | - |
| | α $CH_2$ | 3.87, dd, $J$ = 6.7, $J$ = 16.6;<br>3.63, dd, $J$ = 4.8, $J$ = 16.7 | 42.6 |
| | CO | - | 168.7 |
| Ile | NH | 7.89, d, $J$ = 8.5 | - |
| | α CH | 4.11-4.07, m | 57.5 |
| | β CH | 1.80-1.76, m | 36.3 |
| | γ $CH_2$ | 1.50-1.43, m; 1.08-1.03, m | 24.9 |
| | γ $CH_3$ | 0.87-0.83, m | 15.8 |
| | δ $CH_3$ | 0.84-0.80, m | 11.4 |
| | CO | - | 171.3 |
| Gly (c) | NH | 8.58, dd, $J$ = 4.6, $J$ = 7.3 | - |
| | α $CH_2$ | 4.59, dd, $J$ = 7.7, $J$ = 15.3; | 34.7 |

| | | | |
|---|---|---|---|
| | | 3.93, dd, *J* = 3.2, *J* = 15.4 | |
| | Tz CH | 8.10, s | 123.0 |
| | Tz C | - | 145.4 |
| Phe | α CH | 5.85, dd, *J* = 3.8, *J* = 11.6 | 62.8 |
| | β $CH_2$ | 3.47-3.45, m; | 38.0 |
| | | 3.18, dd, *J* = 11.8, *J* = 14.5 | |
| | Ar CH | 7.24-7.21, m; | 127.4 |
| | | 7.20-7.18, m; | 128.8 |
| | | 7.11, d, *J* = 7.1 | 129.3 |
| | Ar C | | 135.8 |
| | CO | - | 167.8 |
| Pro | α CH | 4.45-4.42, m | 61.0 |
| | β $CH_2$ | 2.06-2.00, m; 1.97-1.91, m | 27.9 |
| | γ $CH_2$ | 1.97-1.88, m | 25.2 |
| | δ $CH_2$ | 3.84-3.78, m | 47.7 |
| | CO | - | 170.4 |

*NMR data of the major conformer

$^1$H NMR: (700 MHz, DMSO); HSQC and HMBC analysis (700 MHz, DMSO)*

| **Amino acid*** | **Atome** | **$^1$H chemical shift** | **$^{13}$C chemical shift** |
|---|---|---|---|
| Val | NH | 8.33, d, *J* = 5.1 | - |
| | α CH | 3.73, dd, *J* = 5.3, *J* = 7.5 | 61.0 |
| | β CH | 1.94-1.89, m | 29.3 |
| | γ $CH_3$ | 0.94, d, *J* = 6.7; | 19.8 |
| | | 0.88-0.85, m | 19.8 |
| | CO | | 171.3 |
| Gly (a) | NH | 8.27-8.22, m | - |
| | α $CH_2$ | 3.78-3.75, m; 3.53-3.50, m | 42.6 |
| | CO | | 168.7 |
| Ala | NH | 7.82, d, *J* = 7.6 | - |
| | α CH | 4.32-4.25, m | 48.6 |
| | β $CH_3$ | 1.11, d, *J* = 7.2 | 18.0 |
| | CO | - | 173.2 |
| Gly (b) | NH | 8.31, t, *J* = 5.4 | - |
| | α $CH_2$ | 3.88, dd, *J* = 5.6, *J* = 15.7; | 43.3 |
| | | 3.45-3.42, m | |
| | CO | - | 169.4 |
| Ile | NH | 7.74, d, *J* = 8.4 | - |
| | α CH | 4.12-4.08, m | 57.5 |
| | β CH | 1.89-1.82, m | 36.5 |
| | γ $CH_2$ | 1.36-1.31, m; 1.11-1.08, m | 24.5 |
| | γ $CH_3$ | 0.83-0.80, m | 15.8 |
| | δ $CH_3$ | 0.81-0.78, m | 11.4 |
| | CO | - | 170.8 |
| Gly (c) | NH | 8.27-8.22, m | - |
| | α $CH_2$ | 4.48-4.45, m; 4.17-4.13, m | 34.7 |
| | Tz CH | 7.79, s | 123.0 |
| | Tz C | - | 144.7 |

| | | | |
|---|---|---|---|
| Phe | α CH | 5.26, t, *J* = 7.7 | 62.4 |
| | β $CH_2$ | 3.49-3.47, m; | 38.2 |
| | | 3.35, dd, *J* = 7.9, *J* = 13.4 | |
| | Ar CH | 7.29-7.26, m; | 128.8 |
| | | 7.27-7.24, m | 127.4 |
| | | 7.18-7.16, m | 129.7 |
| | Ar C | | 135.8 |
| | CO | - | 166.4 |
| Pro | α CH | 4.17-4.13, m | 59.6 |
| | β $CH_2$ | 1.95-1.91, m; 1.87-1.83, m | 32.1 |
| | γ $CH_2$ | 1.63-1.55, m; 1.28-1.24, m | 22.4 |
| | δ $CH_2$ | 3.34-3.30, m; 3.26-3.22, m | 47.3 |
| | CO | - | 171.8 |

*NMR data of the minor conformer

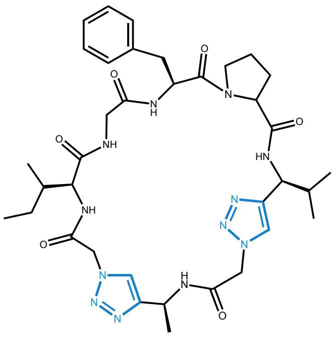

**CP13**; Cyclo(-$VTz_aG_aATz_bG_bIG_cFP$-) **3:1**

$^{1}$H NMR: (700 MHz, DMSO); HSQC and HMBC analysis (700 MHz, DMSO)*

| **Amino acid*** | **Atome** | **[1]H chemical shift** | **[13]C chemical shift** |
|---|---|---|---|
| Val | NH | 7.68, d, *J* = 8.6 | - |
| | α CH | 4.83, t, *J* = 7.5 | 51.4 |
| | β CH | 2.13-2.07, m | 32.6 |
| | γ $CH_3$ | 0.85-0.83, m | 19.3 |
| | | 0.83-0.80, m | 18.9 |
| | Tz CH | 7.81, s | 123.9 |
| | Tz C | - | 147.4 |
| Gly (a) | α $CH_2$ | 5.16, d, *J* = 16.0 | 52.1 |
| | | 5.01, d, *J* = 16.4 | |
| | CO | | 164.9 |
| Ala | NH | 8.89, d, *J* = 7.7 | - |
| | α CH | 5.04, t, *J* = 7.4 | 41.7 |
| | β $CH_3$ | 1.44, d, *J* = 6.9 | 21.0 |
| | Tz CH | 7.71, s | 123.2 |
| | Tz C | - | 149.6 |
| Gly (b) | α $CH_2$ | 4.71, d, *J* = 15.9; | 51.2 |
| | | 5.09, d, *J* = 16.4 | |
| | CO | - | 165.9 |
| Ile | NH | 8.65, d, *J* = 7.0 | - |
| | α CH | 4.08, t, *J* = 7.6 | 58.2 |
| | β CH | 1.72-1.66, m | 36.3 |
| | γ $CH_2$ | 1.49-1.45, m; 1.17-1.10, m | 29.3 |

| | | | |
|---|---|---|---|
| | γ $CH_3$ | 0.85-0.81, m | 15.6 |
| | δ $CH_3$ | 0.85-0.81, m | 11.4 |
| | CO | - | 171.6 |
| Gly (c) | NH | 8.60-8.56, m | - |
| | α $CH_2$ | 4.07-4.02, m;<br>3.30, dd, $J = 2.2$, $J = 16.5$ | 42.4 |
| | CO | - | 168.9 |
| Phe | NH | 7. 09, d, $J = 7.7$ | - |
| | α CH | 4.67-4.62, m | 52.4 |
| | β $CH_2$ | 2.99, dd, $J = 3.4$, $J = 14.2$;<br>2.78, dd, $J = 10.6$, $J = 14.2$ | 36.5 |
| | Ar CH | 7.27-7.24, m; | 128.8 |
| | | 7.21-7.18, m; | 126.7 |
| | | 7.11-7.10, m | 129.8 |
| | Ar C | | 138.5 |
| | CO | - | ND |
| Pro | α CH | 4.31, d, $J = 8.5$ | 61.0 |
| | β $CH_2$ | 2.06-1.97, m; 1.90-1.85, m | 27.1 |
| | γ $CH_2$ | 1.90-1.85, m; 1.84-1.80, m | 24.9 |
| | δ $CH_2$ | 3.60-3.56, m | 47.4 |
| | CO | - | 170.6 |

*NMR data of the major conformer

$^1$H NMR: (700 MHz, DMSO); HSQC and HMBC analysis (700 MHz, DMSO)*

| **Amino acid*** | **Atome** | **$^1$H chemical shift** | **$^{13}$C chemical shift** |
|---|---|---|---|
| Val | NH | 8.63, d, $J = 9.4$ | - |
| | α CH | 4.55, d, $J = 9.3$ | |
| | β CH | 2.13-2.07, m | |
| | γ $CH_3$ | 0.92, d, $J = 6.5$ | 15.8 |
| | | 0.74, d, $J = 6.5$ | 20.0 |
| | Tz CH | 7.52, s | |
| | Tz C | - | |
| Gly (a) | α $CH_2$ | 5.21, d, $J = 16.5$;<br>4.99-4.95, m | 51.9 |
| | CO | | |
| Ala | NH | 8.89-8.87 | - |
| | α CH | 5.06-5.03 | |
| | β $CH_3$ | 1.51, d, $J = 7.1$ | 20.7 |
| | Tz CH | 7.81, s | |
| | Tz C | - | |
| Gly (b) | α $CH_2$ | 5.14-5.10, m | 52.4 |
| | CO | - | |
| Ile | NH | 8.56, d, $J = 4.5$ | - |
| | α CH | 4.06-4.04 | 59.3 |
| | β CH | 1.80-1.76, m | 36.1 |
| | γ $CH_2$ | 1.50-1.43, m | |
| | γ $CH_3$ | 0.87-0.84, m | 14.3 |
| | δ $CH_3$ | 0.89-0.87, m | 11.5 |

| | | | |
|---|---|---|---|
| | CO | - | |
| Gly (c) | NH | 8.52, t, *J* = 5.7 | - |
| | α $CH_2$ | 3.82-3.78, m; 3.69-3.65, m | |
| | CO | - | |
| Phe | NH | 7.69-7.67 | |
| | α CH | 4.13-4.09, m | |
| | β $CH_2$ | 2.66-2.61, m; ND | |
| | Ar CH | | |
| | Ar C | | |
| | CO | - | |
| Pro | α CH | 3.26-3.22, m | |
| | β $CH_2$ | 1.59-1.54, m | |
| | γ $CH_2$ | 1.31-1.27, m | |
| | δ $CH_2$ | 3.20-3.17, m; 3.14-3.13, m | |
| | CO | - | |

*NMR data of the minor conformer

# VI. NMR spectra of final cyclic peptides

**CP0**; Cyclo(-VG$_a$AG$_b$IG$_c$FP-); $^1$H; DMSO-d6; 700 MHz; 3:1 ratio

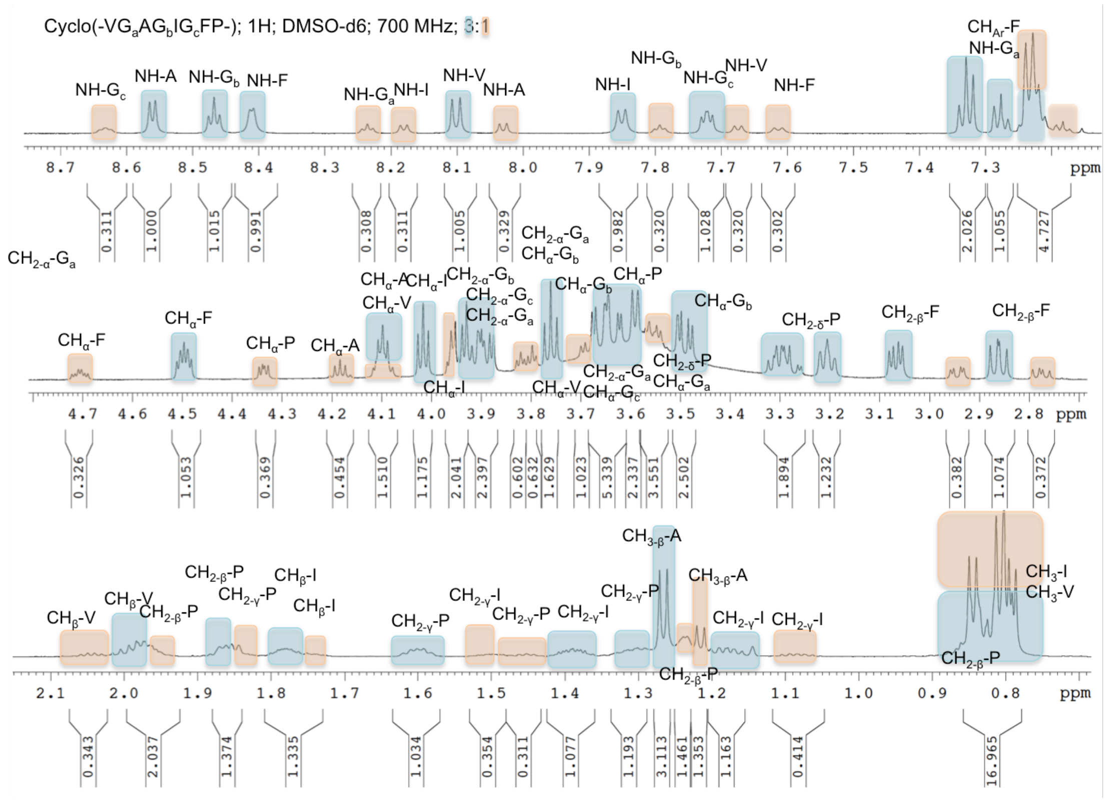

**CP0**; Cyclo(-VG$_a$AG$_b$IG$_c$FP-); HSQC; DMSO-d6; 700 MHz

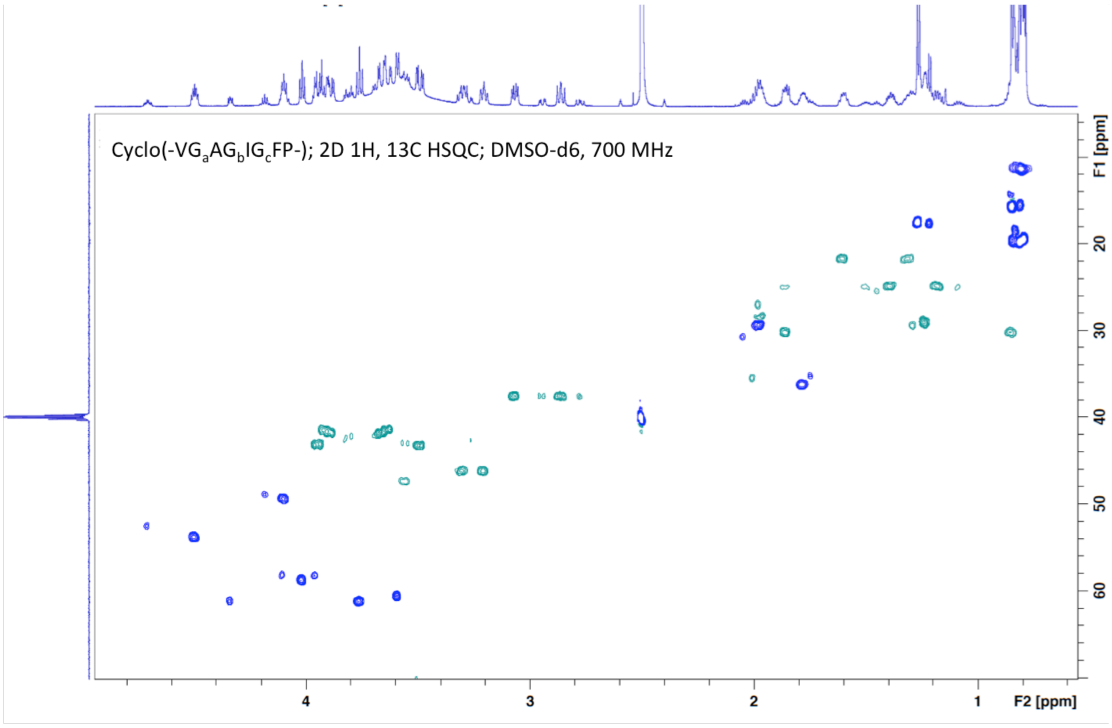

**CP1**; Cyclo(-VTzG$_a$AG$_b$IG$_c$FP-); $^1$H; DMSO-d6; 700 MHz; 4:1 ratio

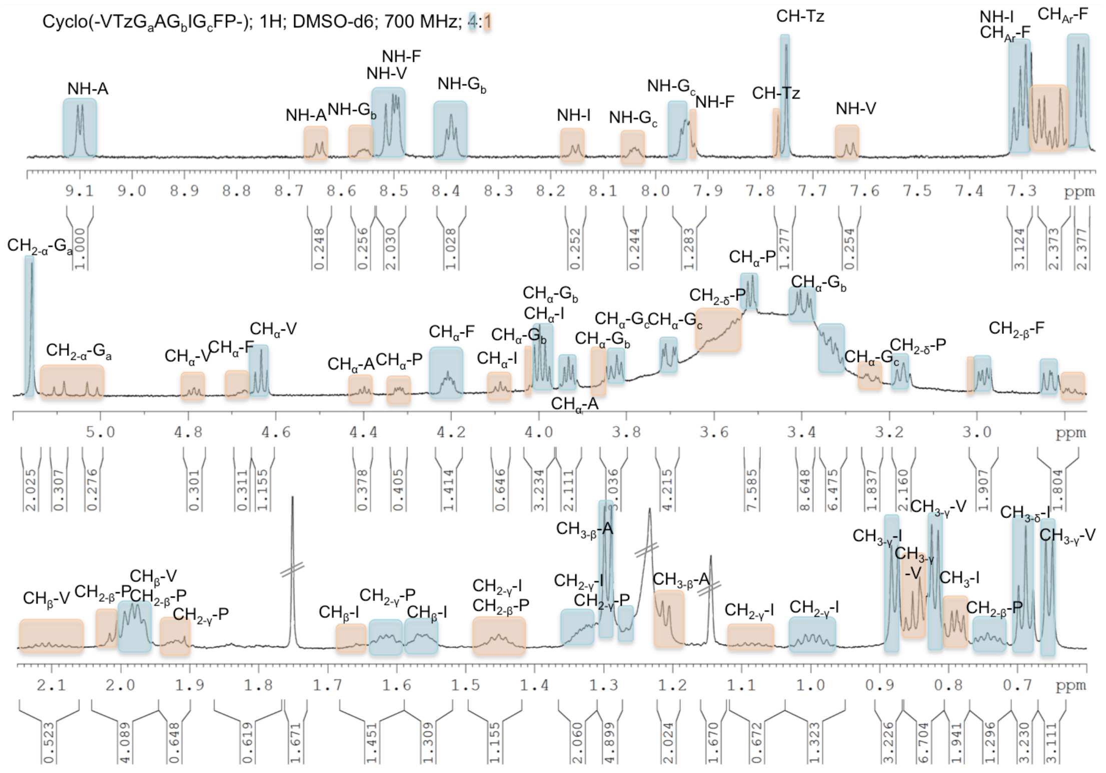


**CP1**; Cyclo(-VTzG$_a$AG$_b$IG$_c$FP-); HSQC; DMSO-d6; 700 MHz

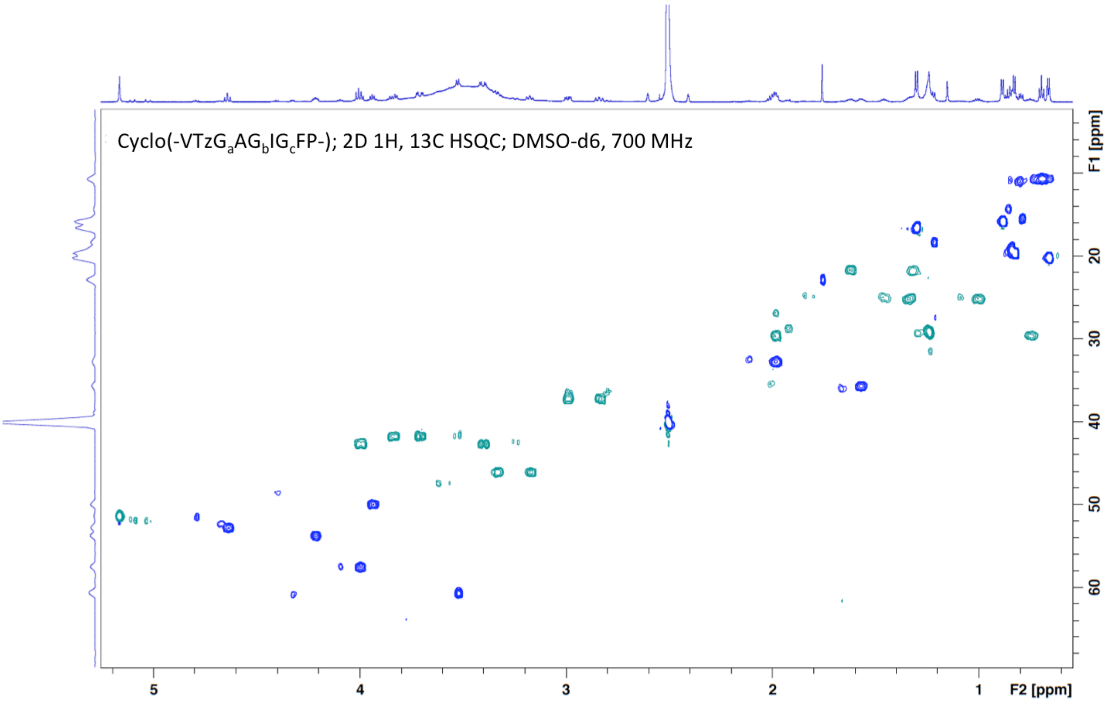

**CP2**; Cyclo(-VG$_a$TzAG$_b$IG$_c$FP-); $^1$H; DMSO-d6; 700 MHz; 1:1 ratio

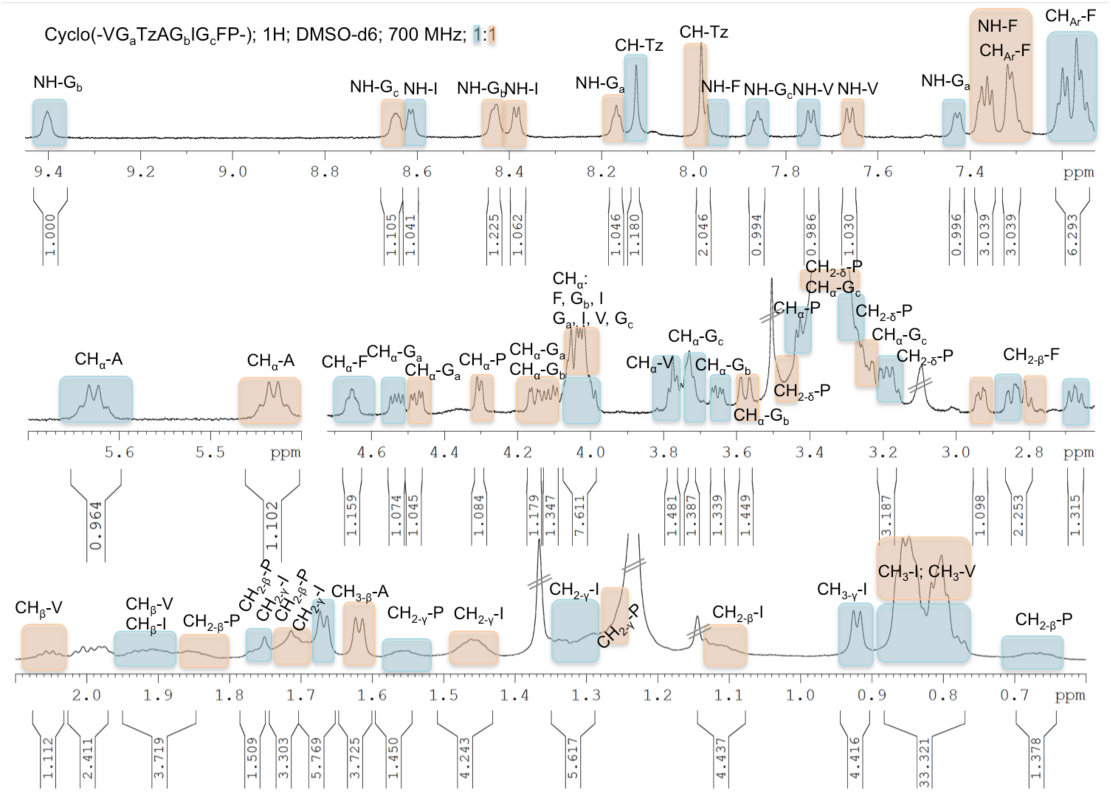

**CP2**; Cyclo(-VG$_a$TzAG$_b$IG$_c$FP-); HSQC; DMSO-d6; 700 MHz

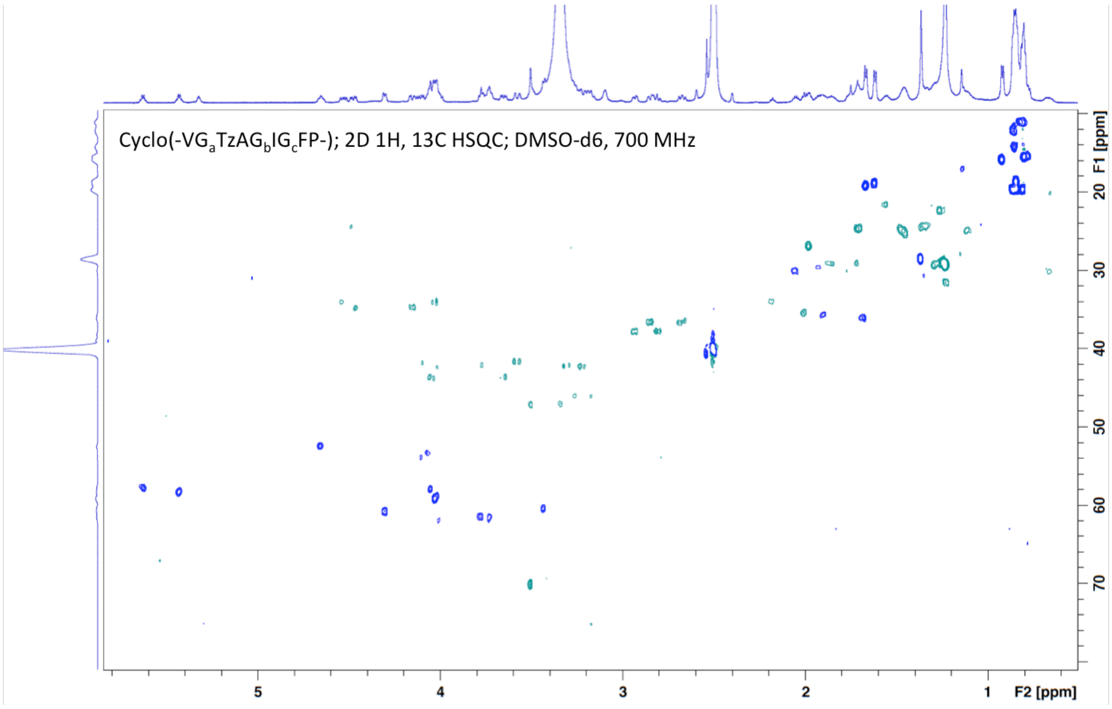

**CP3**; Cyclo(-VG$_a$ATzG$_b$IG$_c$FP-); $^1$H; DMSO-d6; 700 MHz; 1:0.9 ratio

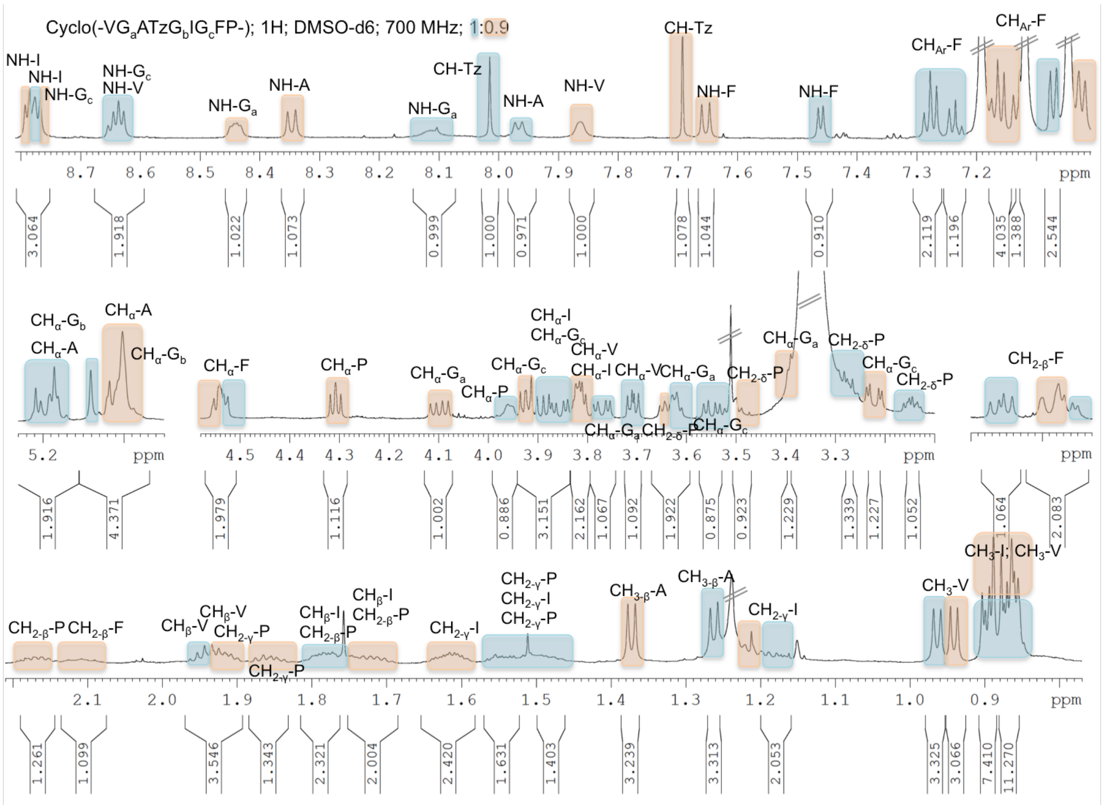


**CP3**; Cyclo(-VG$_a$ATzG$_b$IG$_c$FP-); HSQC; DMSO-d6; 700 MHz

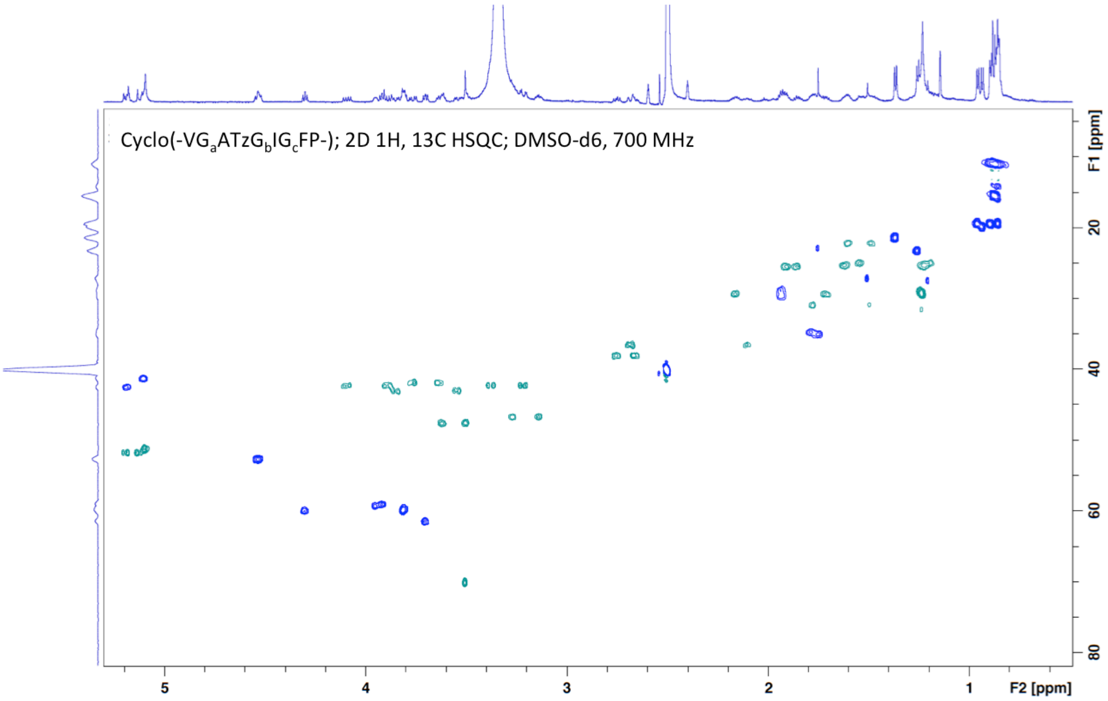

**CP4**; Cyclo(-VG$_a$AG$_b$TzIG$_c$FP-); $^1$H; DMSO-d6; 700 MHz; 1:1 ratio

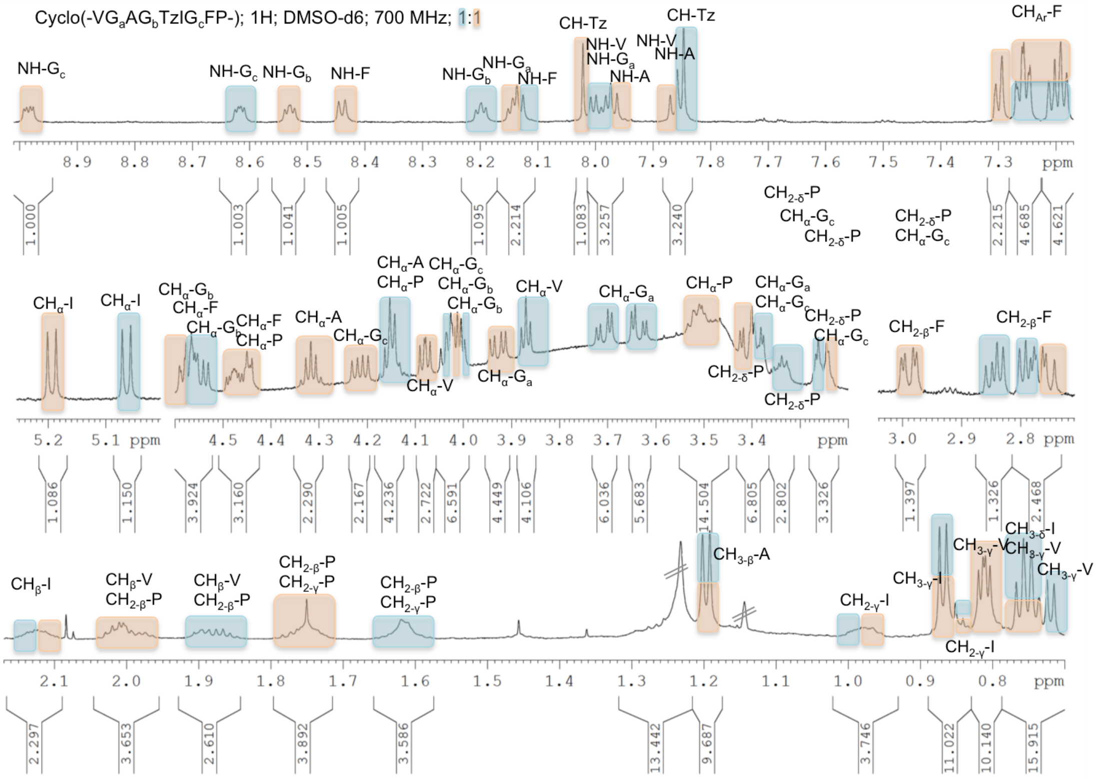

**CP4**; Cyclo(-VG$_a$AG$_b$TzIG$_c$FP-); HSQC; DMSO-d6; 700 MHz

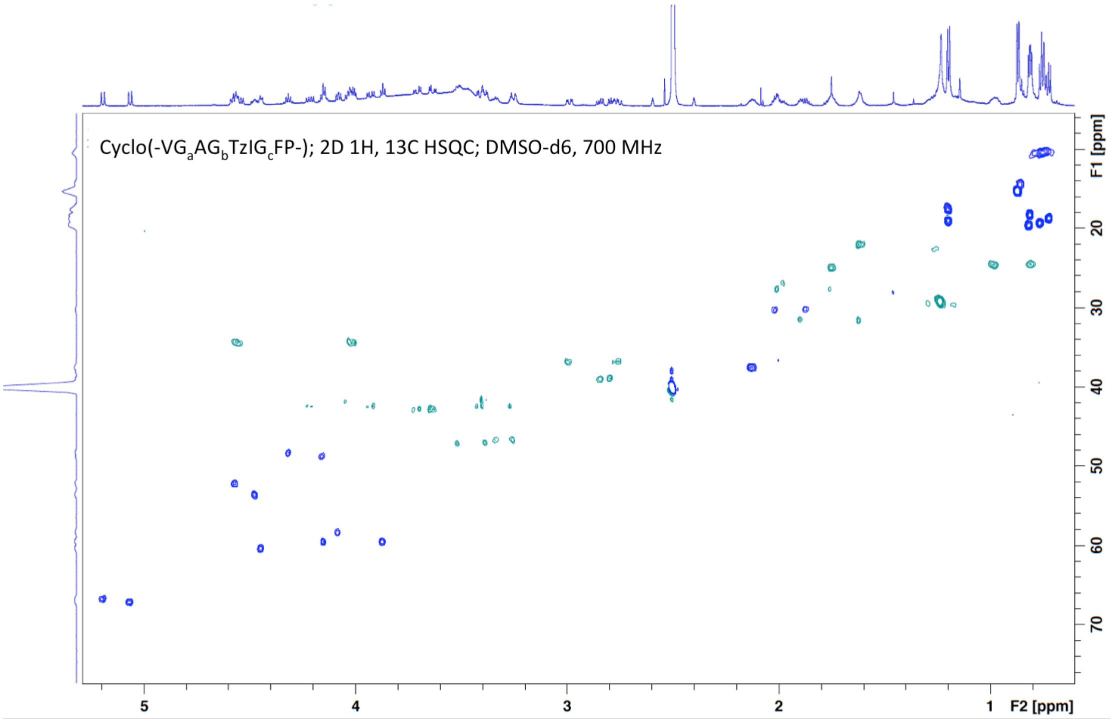

**CP5**; Cyclo(-VG$_a$AG$_b$ITzG$_c$FP-); $^1$H; DMSO-d6; 700 MHz; 1:0.6 ratio

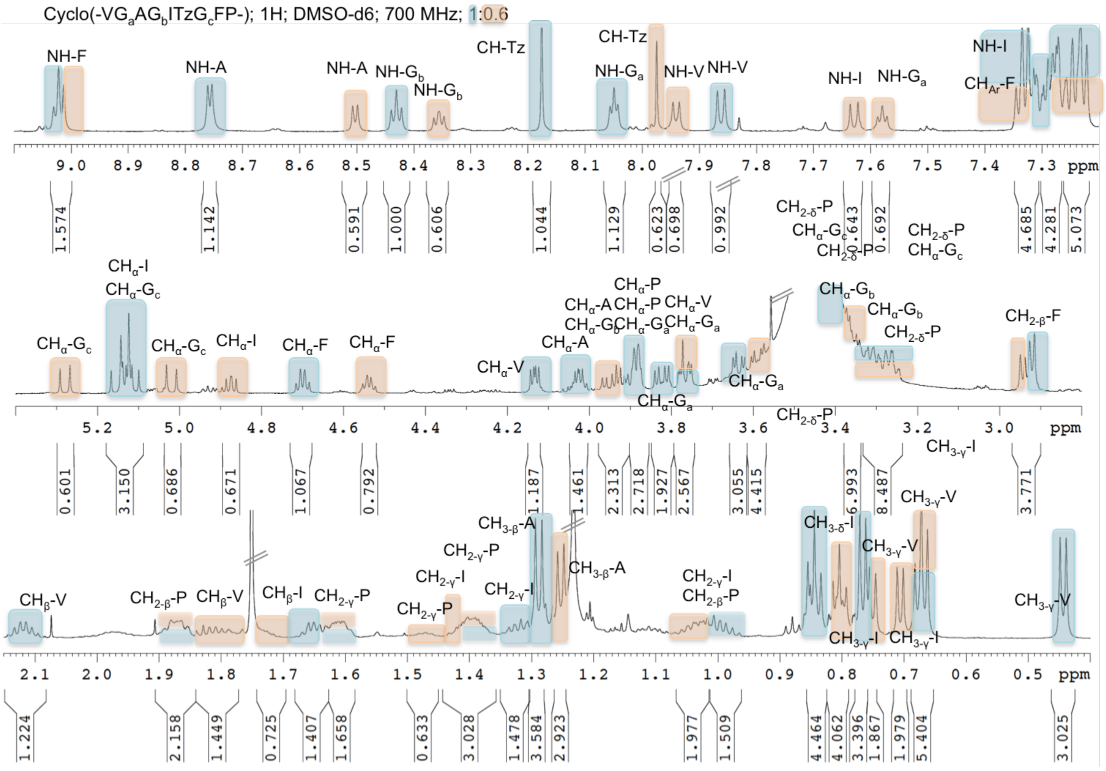


**CP5**; Cyclo(-VG$_a$AG$_b$ITzG$_c$FP-); HSQC; DMSO-d6; 700 MHz

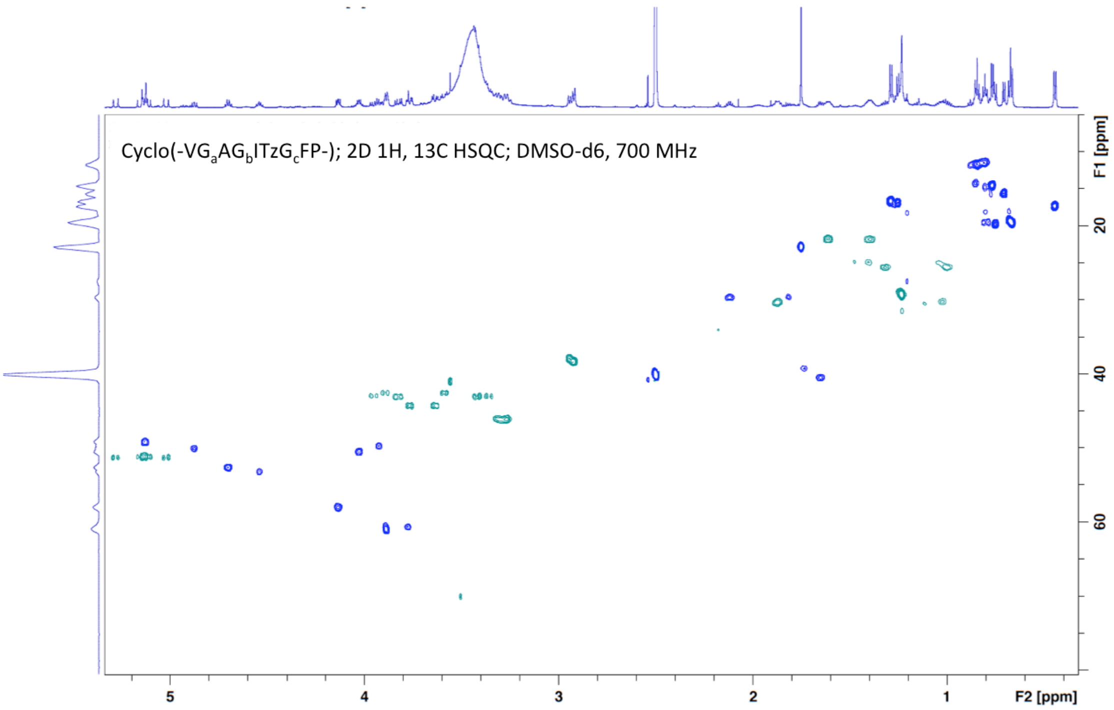

**CP6**; Cyclo(-VG$_a$AG$_b$IG$_c$TzFP-); $^1$H; DMSO-d6; 700 MHz; 2:1 ratio

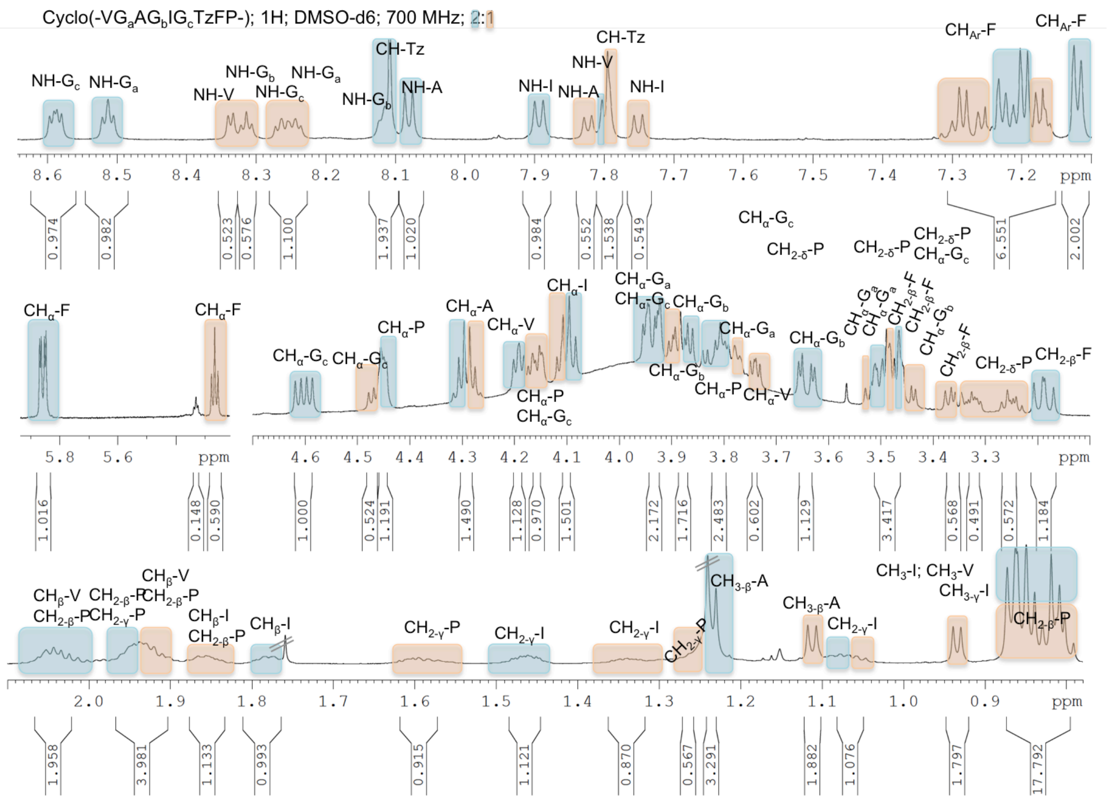


**CP6**; Cyclo(-VG$_a$AG$_b$IG$_c$TzFP-); HSQC; DMSO-d6; 700 MHz

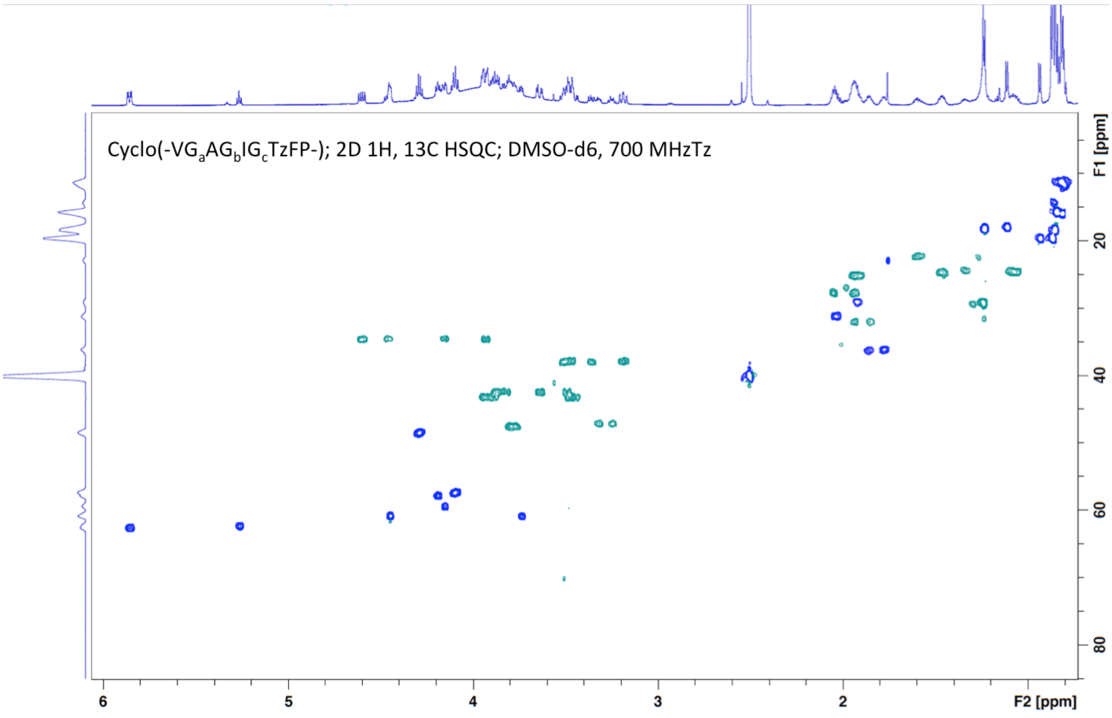

**CP13**; Cyclo(-VTzG$_a$ATzG$_b$IG$_c$FP-); $^1$H; DMSO-d6; 700 MHz; 3:1 ratio

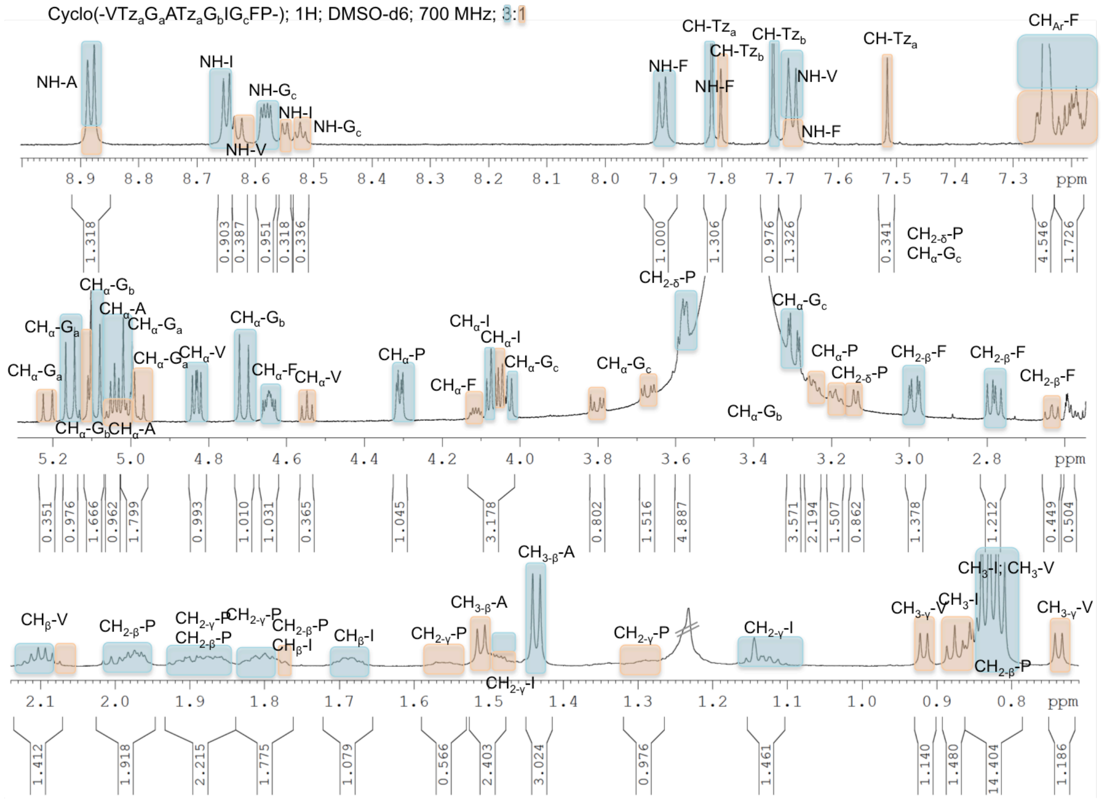


**CP13**; Cyclo(-VTzG$_a$ATzG$_b$IG$_c$FP-); HSQC; DMSO-d6; 700 MHz

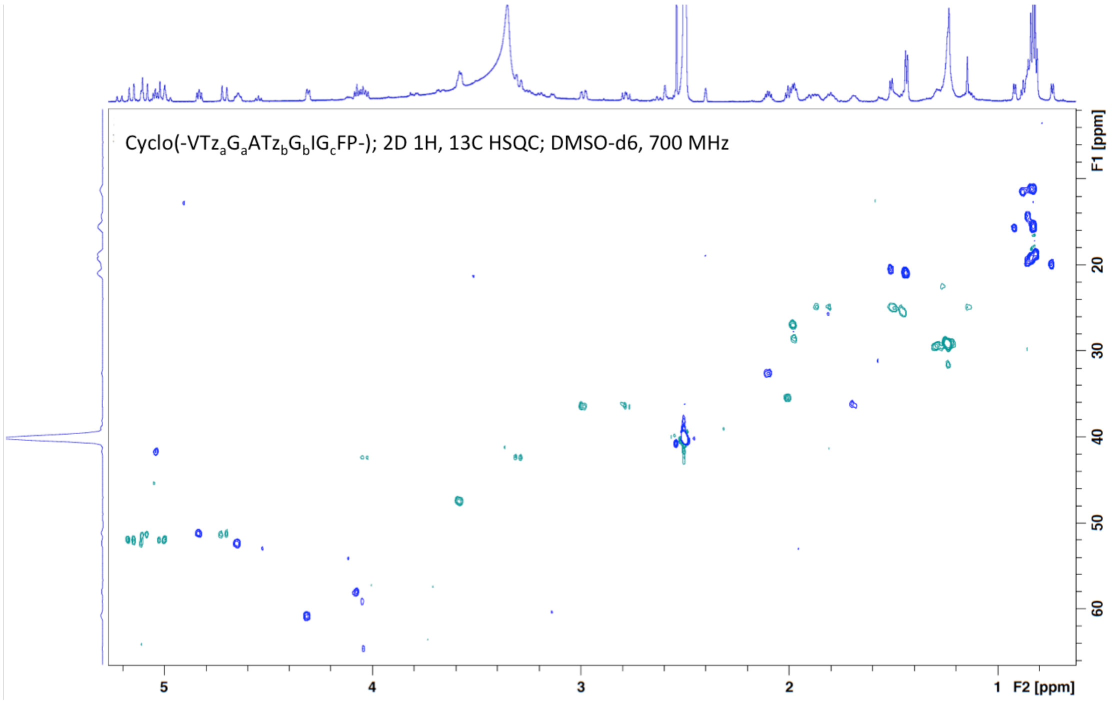

**CP135**; Cyclo(-VTzG$_a$ATzG$_b$ITzG$_c$FP-); $^{1}$H; DMSO-d6; 700 MHz; 1:1.6:1.1:x ratio*

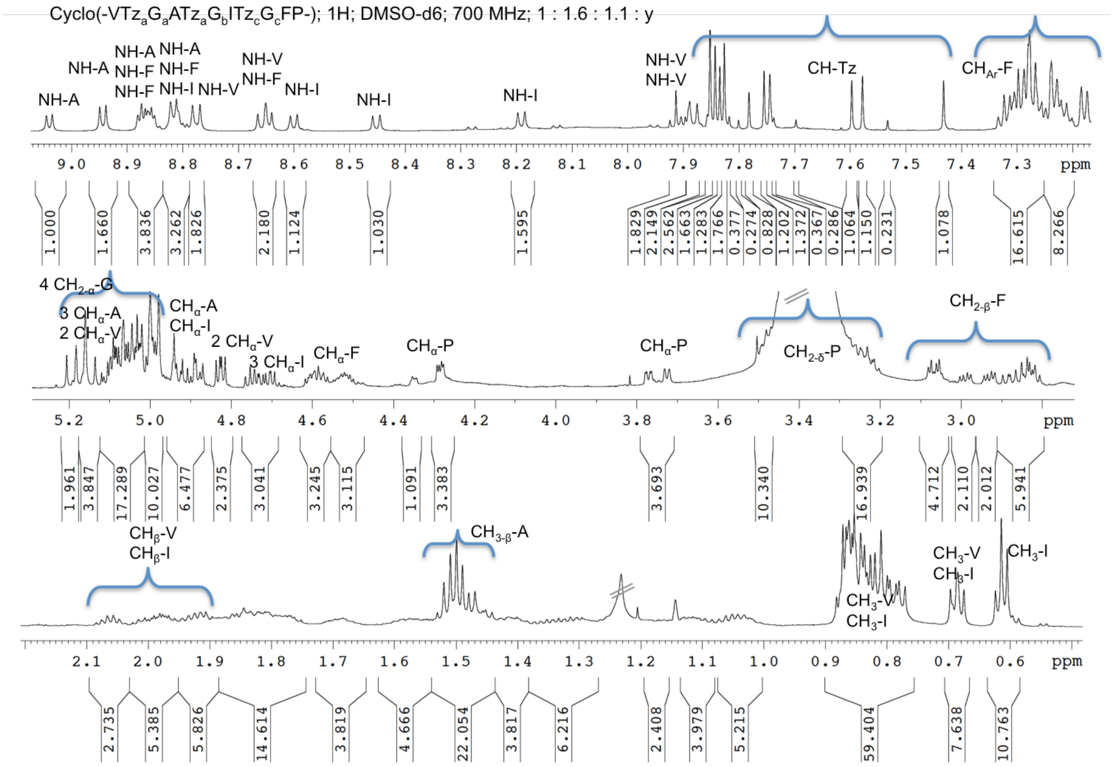


The full ratio of all four major species is not determined. The individual sequences of all four major species are not determined.

**CP135**; Cyclo(-VTzG$_a$ATzG$_b$ITzG$_c$FP-); HSQC; DMSO-d6; 700 MHz

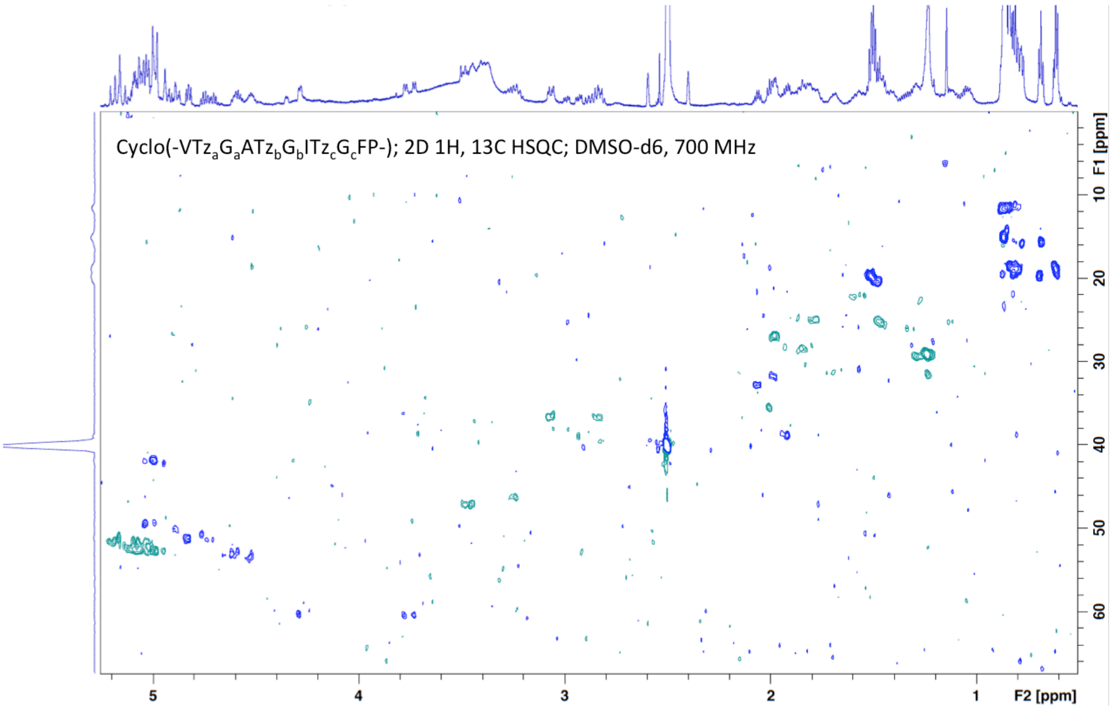

## VII. EXSY NMR of final cyclic peptides

**CP0**; Cyclo(-VG$_a$AG$_b$IG$_c$FP-); EXSY

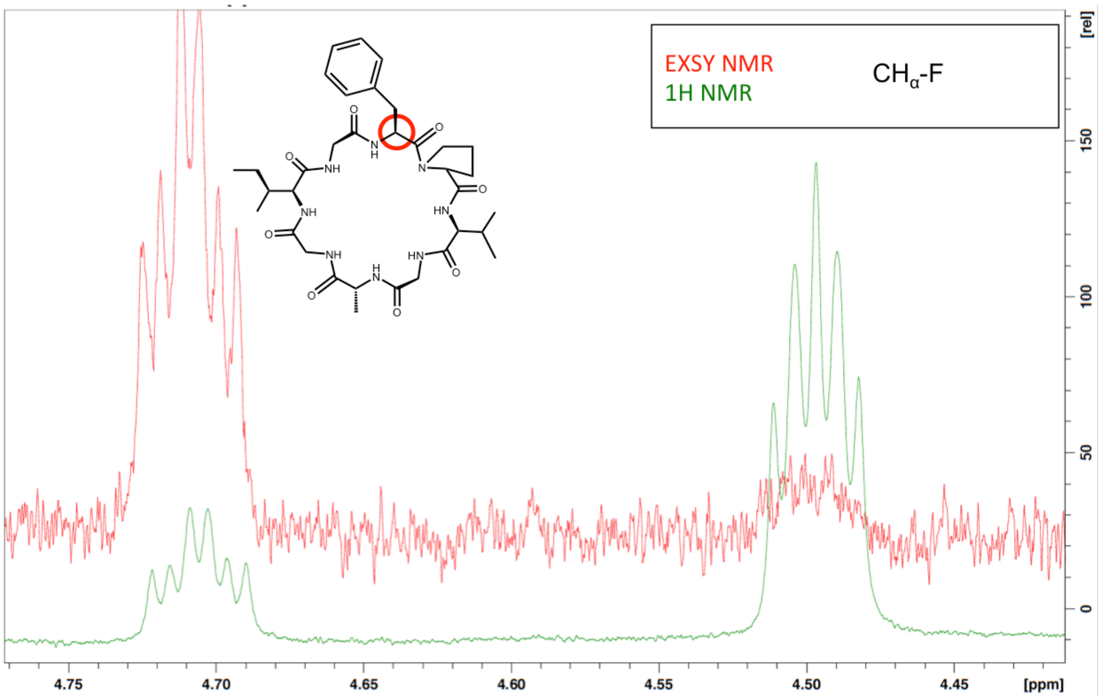


**CP1**; Cyclo(-VTzG$_a$AG$_b$IG$_c$FP-); EXSY

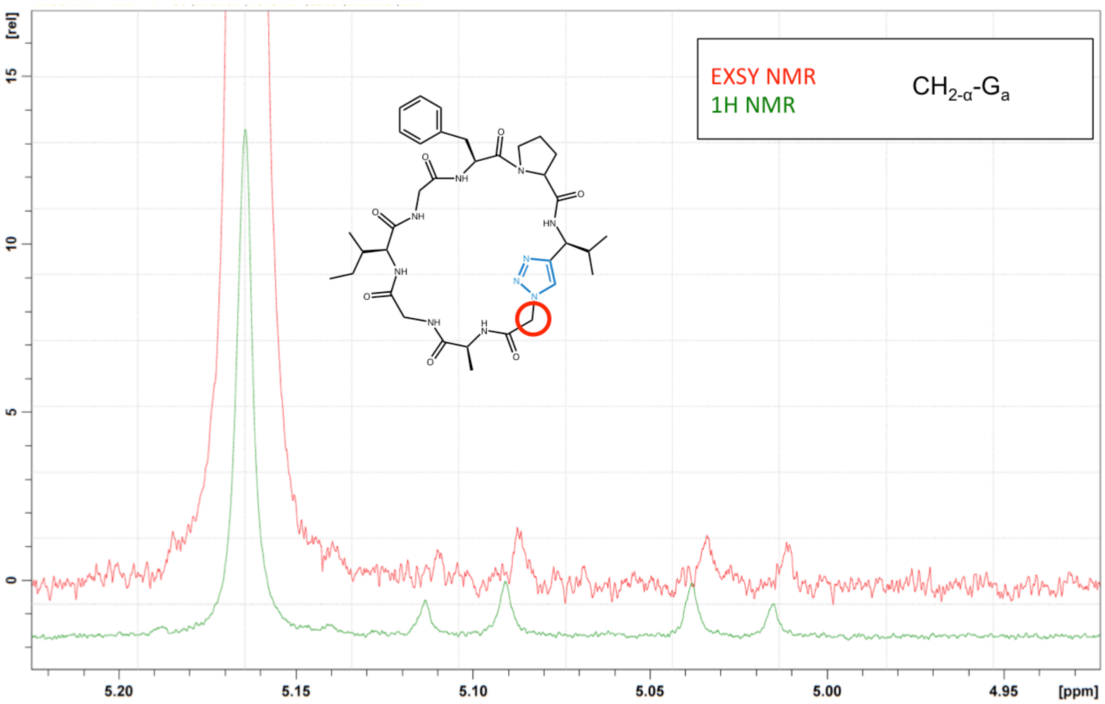


**CP2**; Cyclo(-VG$_a$TzAG$_b$IG$_c$FP-); EXSY

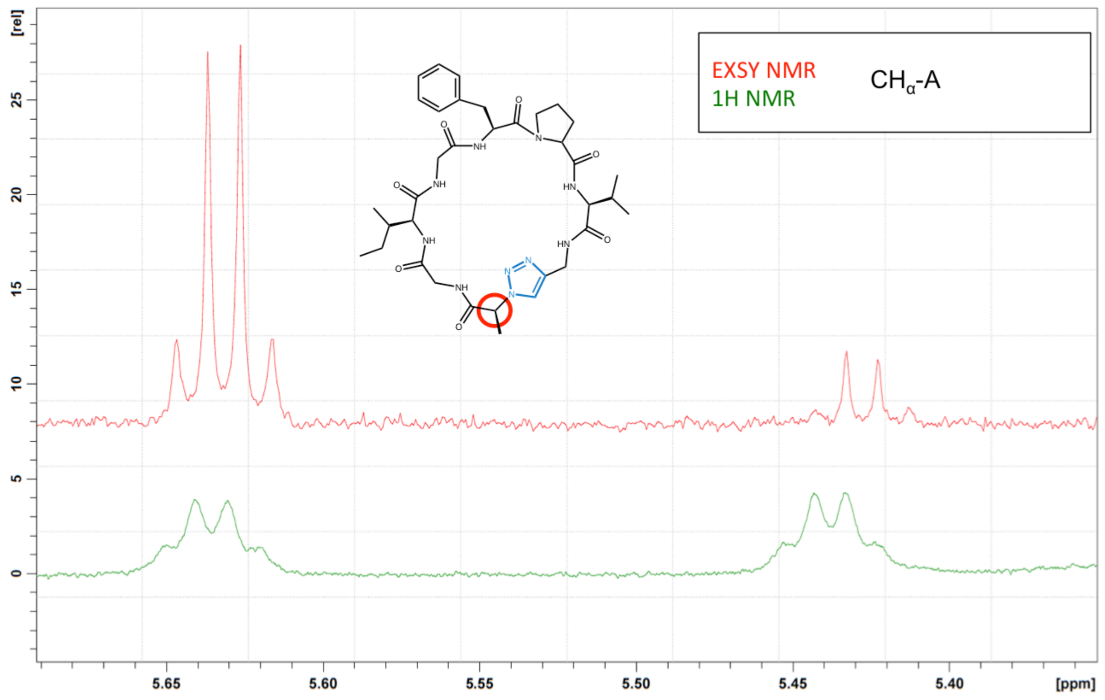


**CP3**; Cyclo(-VG$_a$ATzG$_b$IG$_c$FP-); EXSY

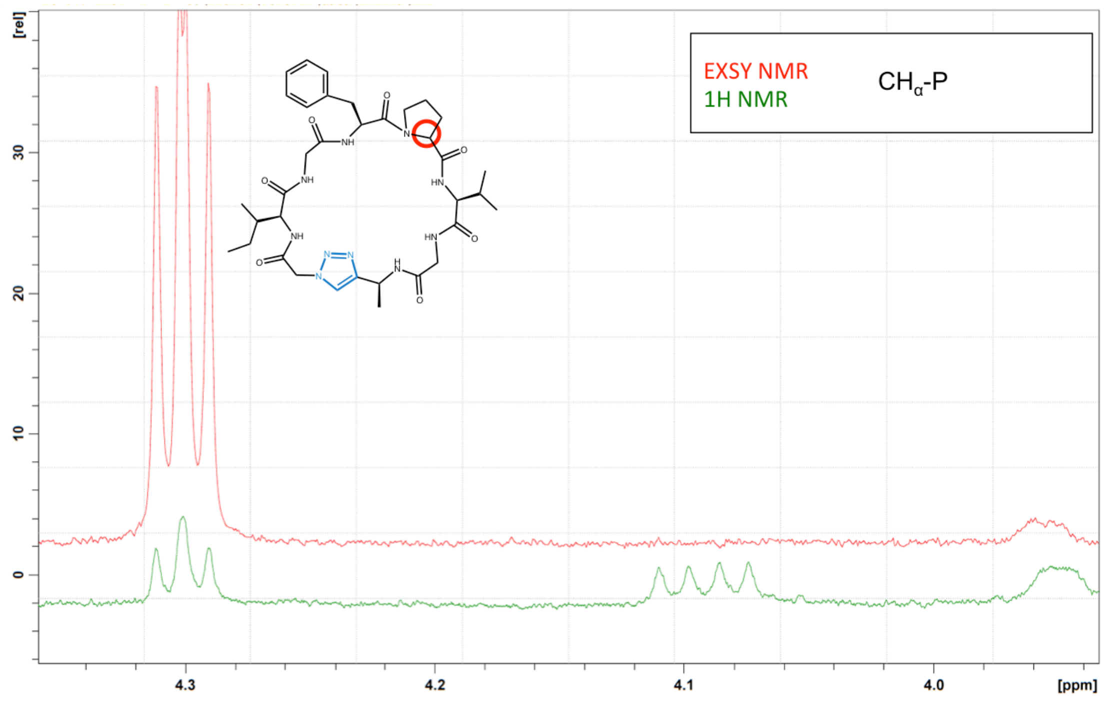

**CP4**; Cyclo(-VG$_a$AG$_b$TzIG$_c$FP-); EXSY

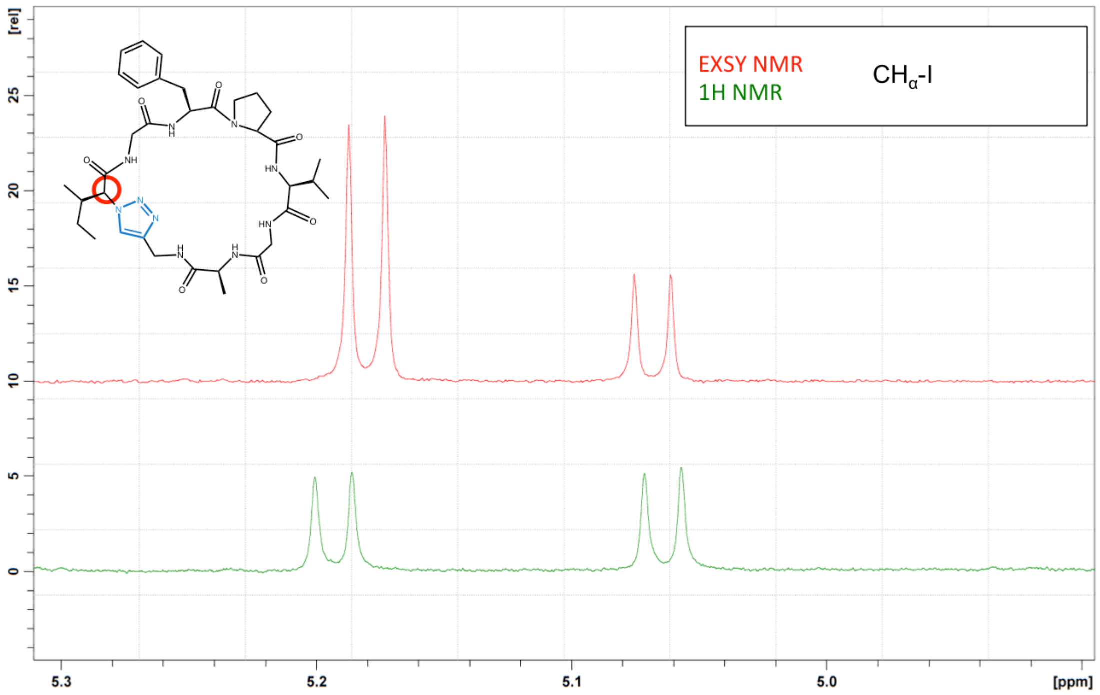


**CP5**; Cyclo(-VG$_a$AG$_b$ITzG$_c$FP-); EXSY

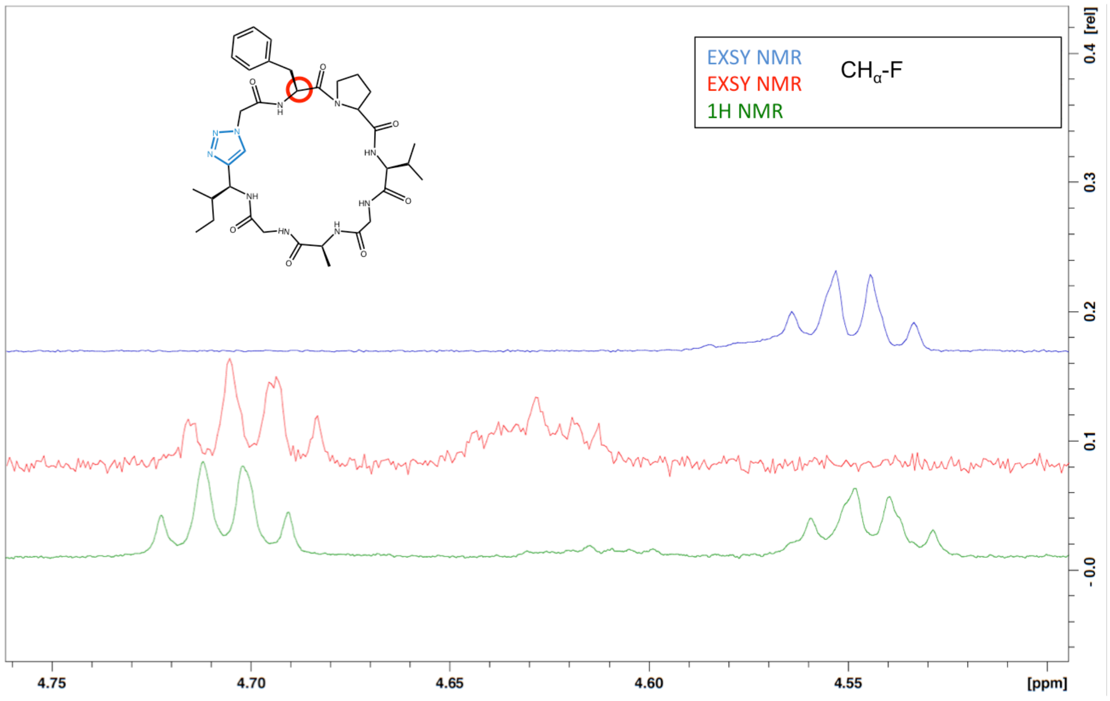

**CP6**; Cyclo(-VG$_a$AG$_b$IG$_c$TzFP-); EXSY

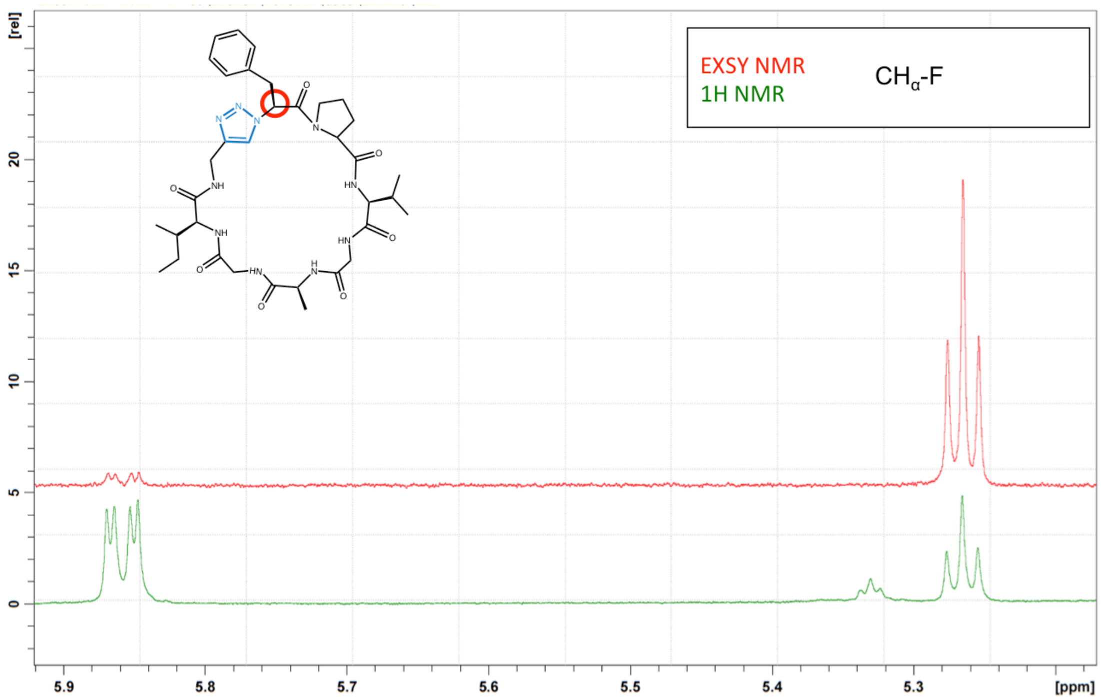


**CP13**; Cyclo(-VTzG$_a$ATzG$_b$IG$_c$FP-); EXSY

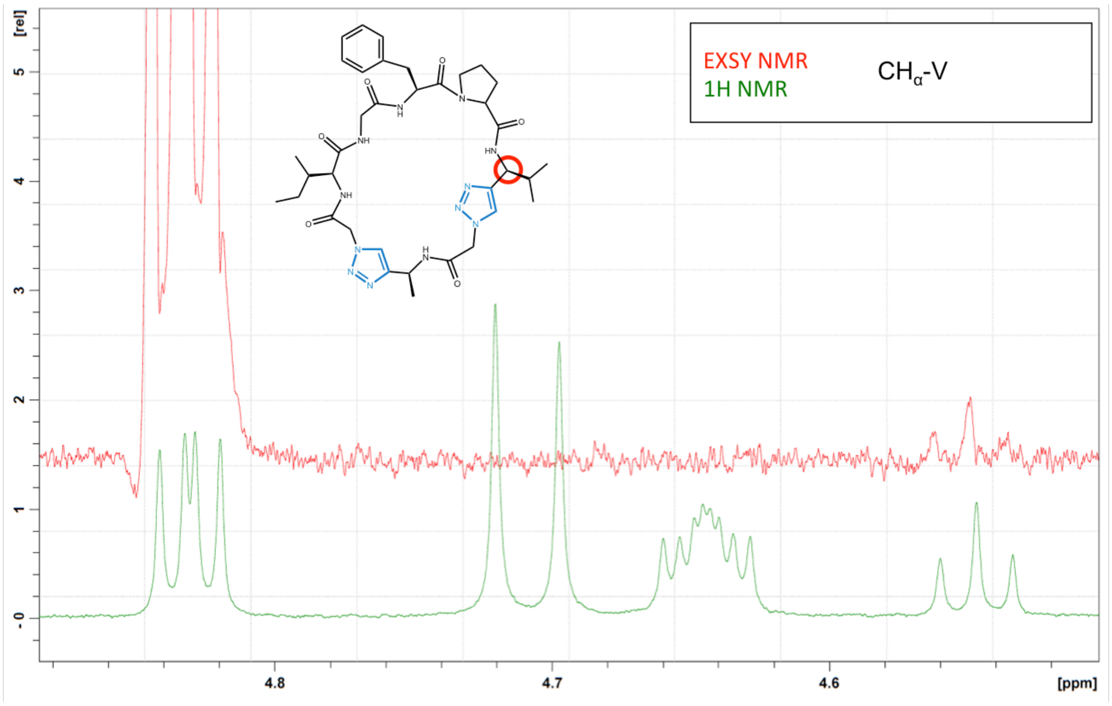

**CP135**; Cyclo(-VTzG$_a$ATzG$_b$ITzG$_c$FP-); EXSY

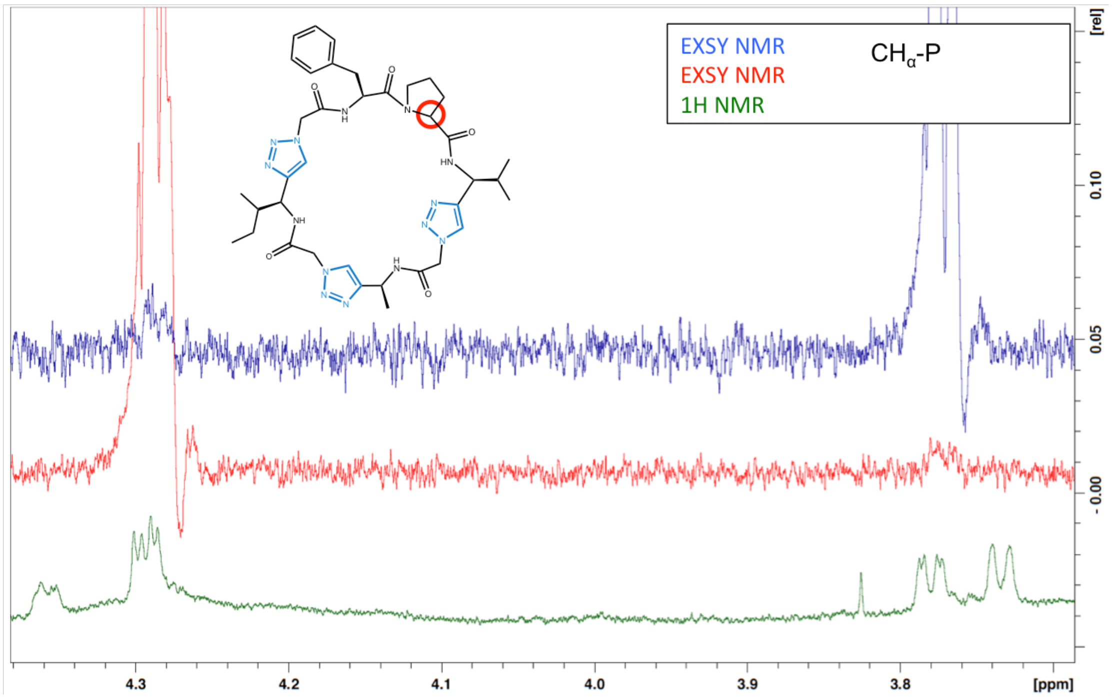


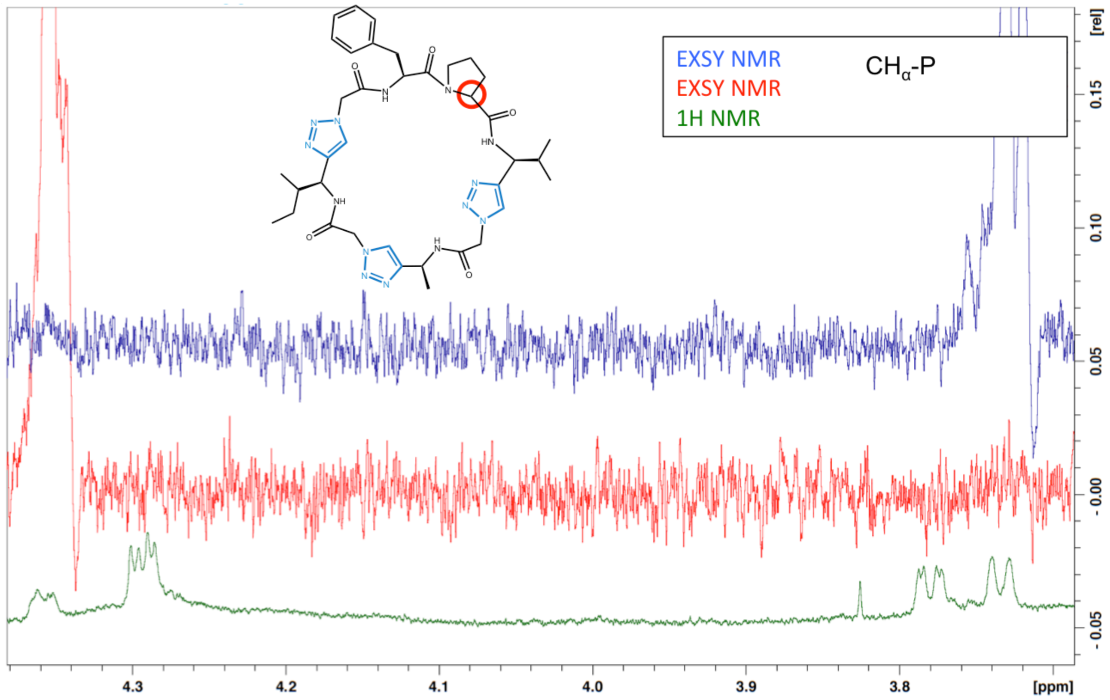

# VIII. MS-MS data of final cyclic peptides

**CP0**; Cyclo(-VG$_a$AG$_b$IG$_c$FP-)

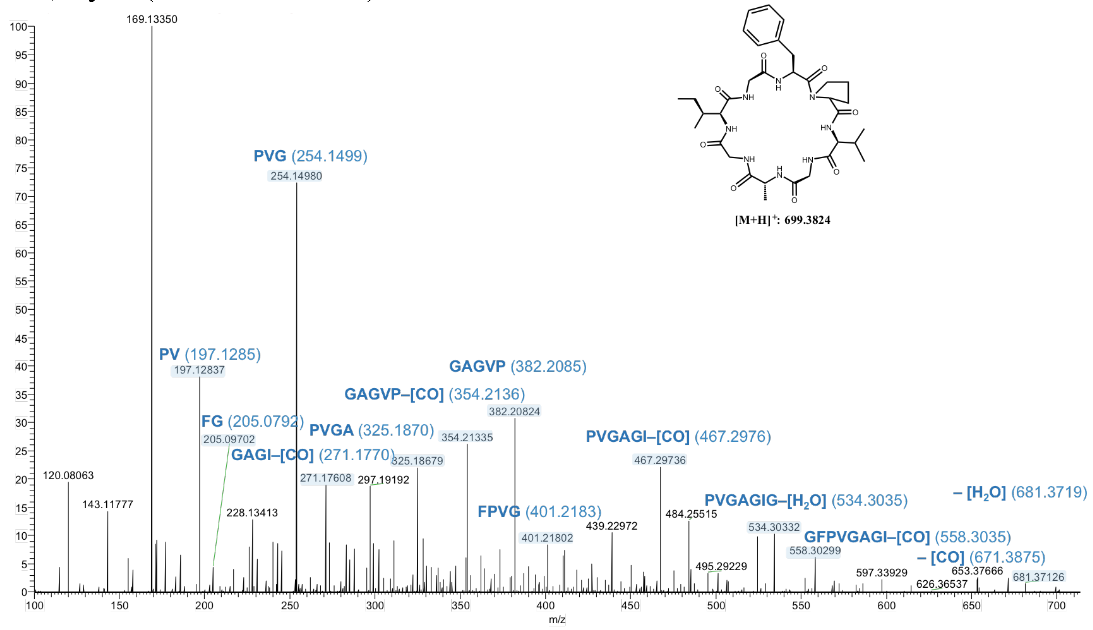


**CP1**; Cyclo(-VTzG$_a$AG$_b$IG$_c$FP-)

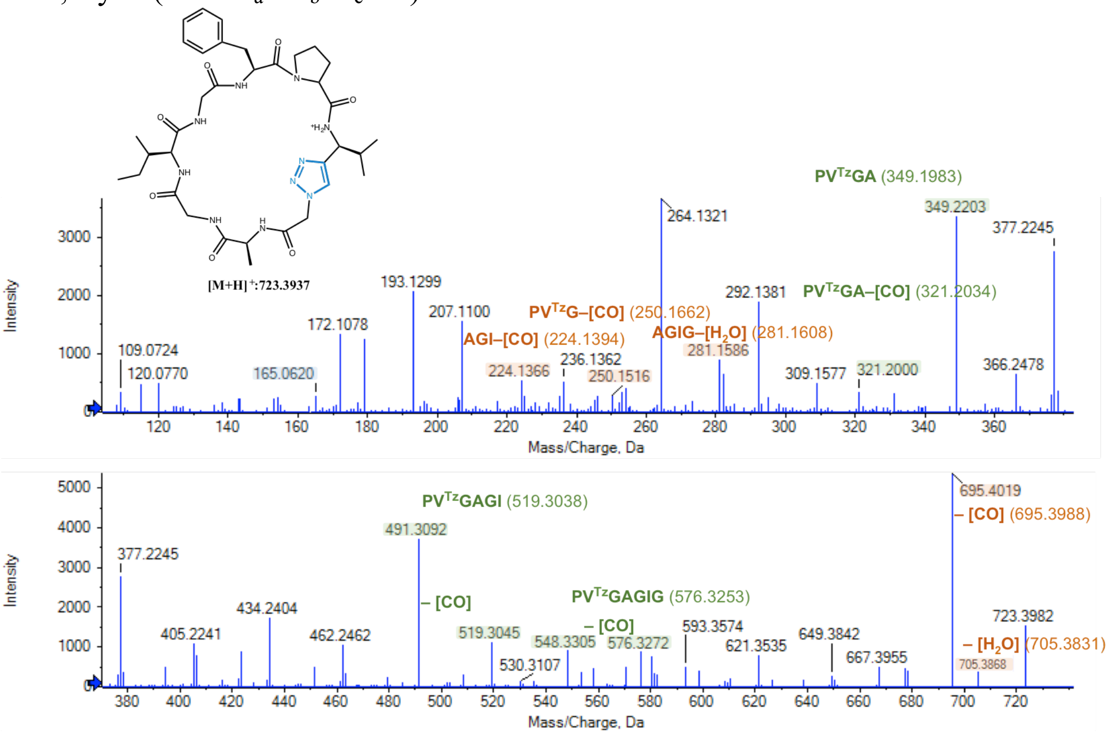

**CP2**; Cyclo(-VG$_a$TzAG$_b$IG$_c$FP-)

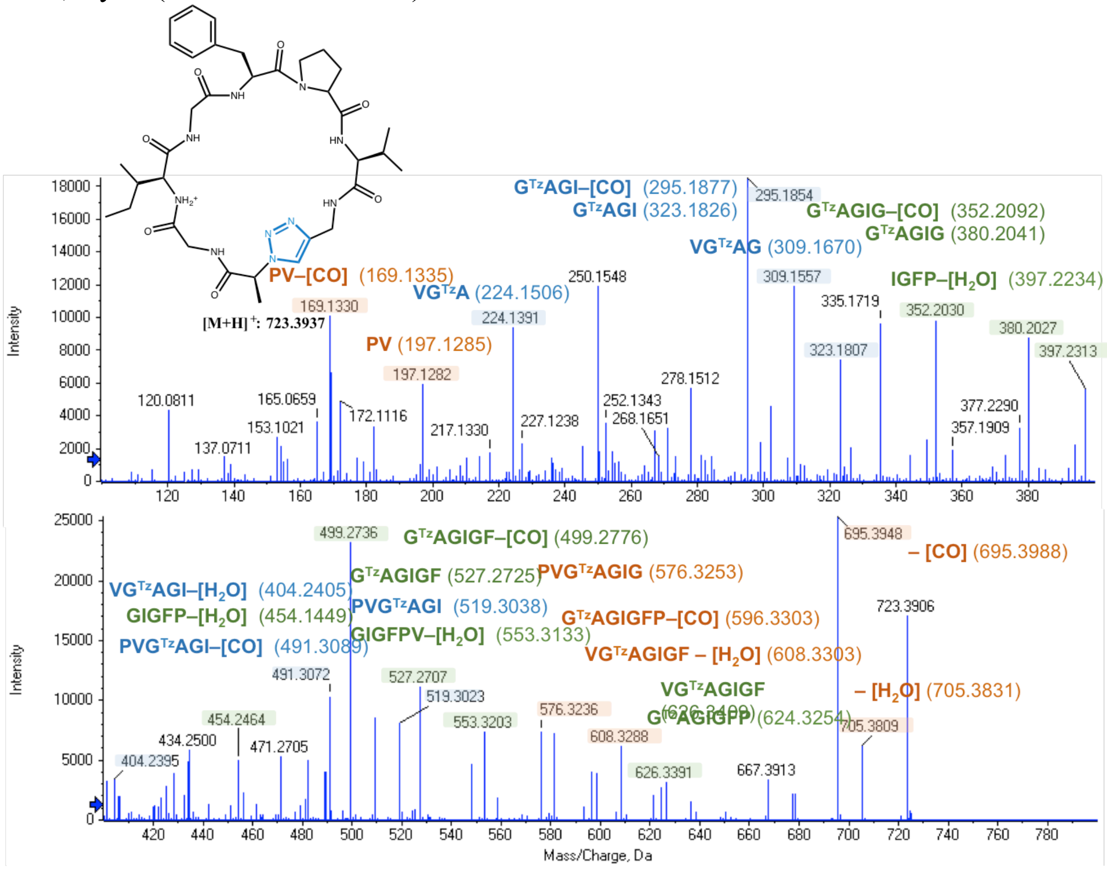


**CP3**; Cyclo(-VG$_a$ATzG$_b$IG$_c$FP-)

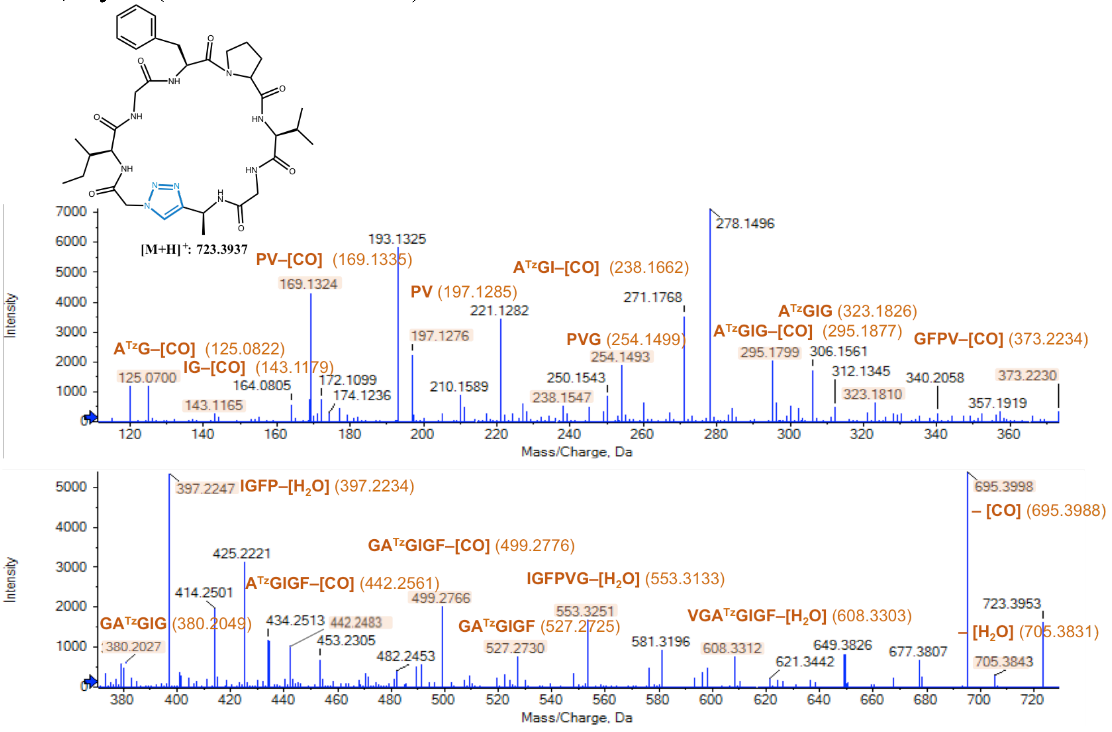


The MS-MS fragmentation data of both cyclic compounds observed in the crude mixture were identical.

**CP4**; Cyclo(-VG$_a$AG$_b$TzIG$_c$FP-)

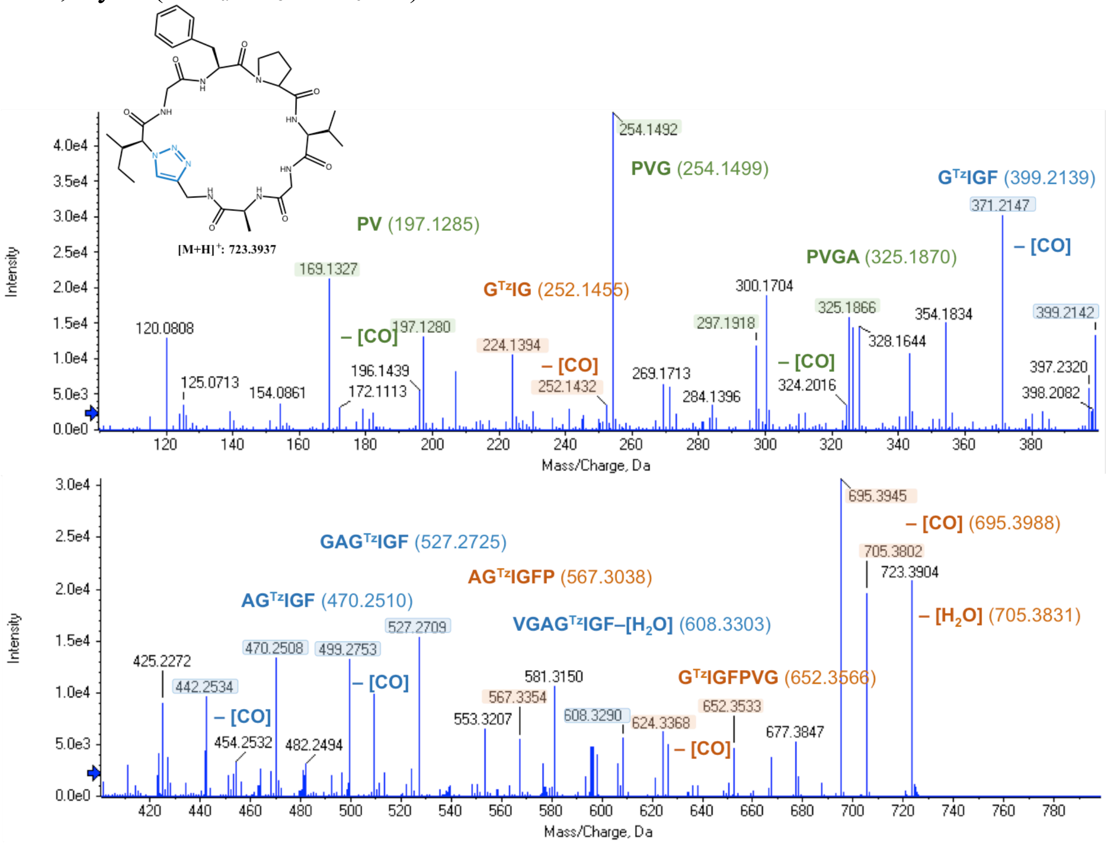


**CP5**; Cyclo(-VG$_a$AG$_b$ITzG$_c$FP-)

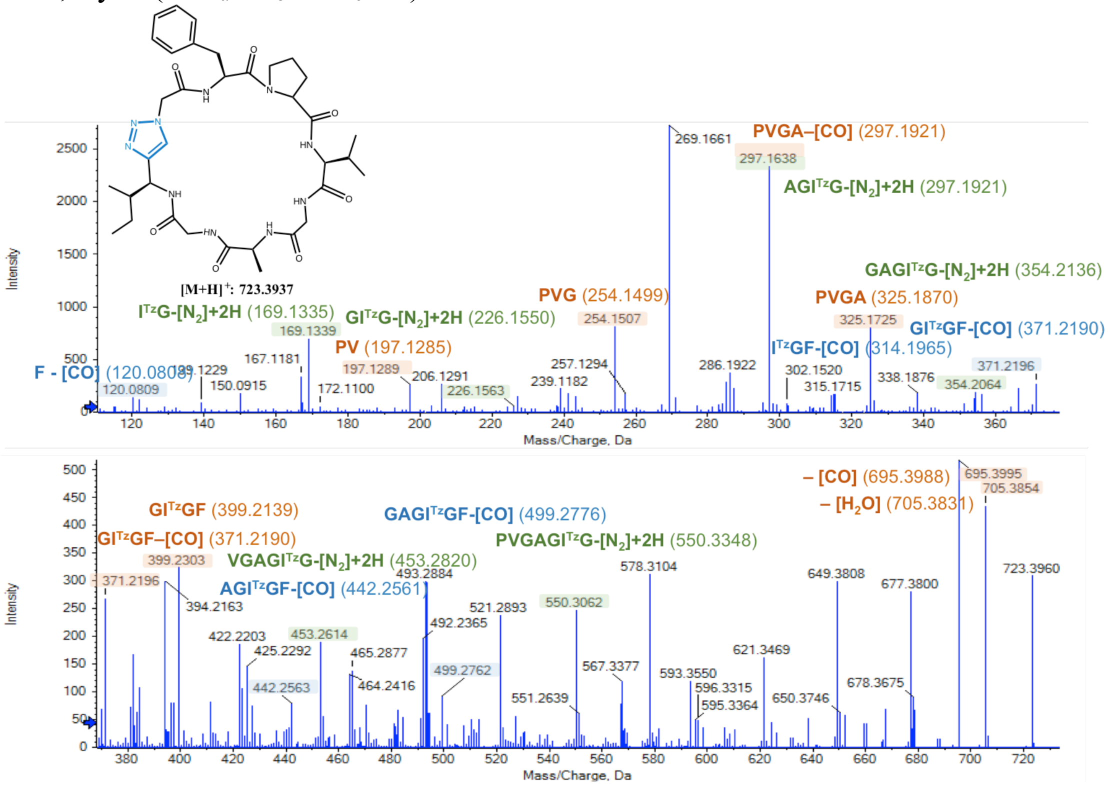

**CP6**; Cyclo(-VG$_a$AG$_b$IG$_c$TzFP-)

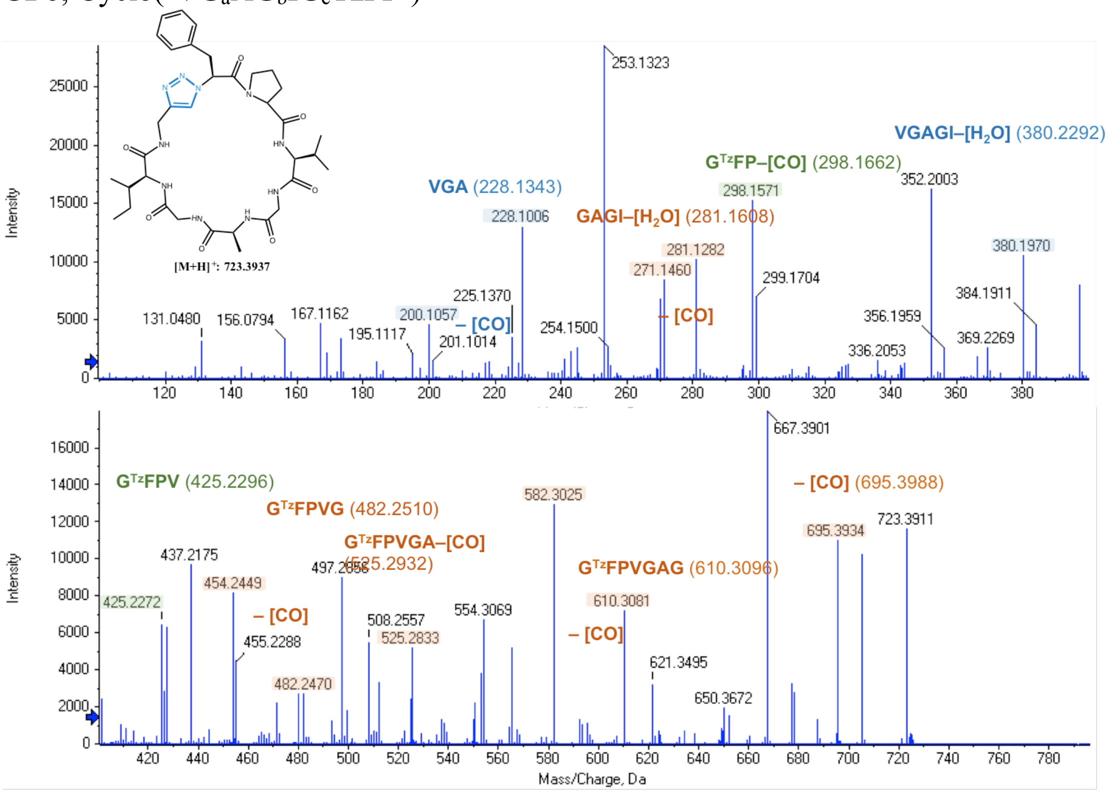


**CP13**; Cyclo(-VTzG$_a$ATzG$_b$IG$_c$FP-)

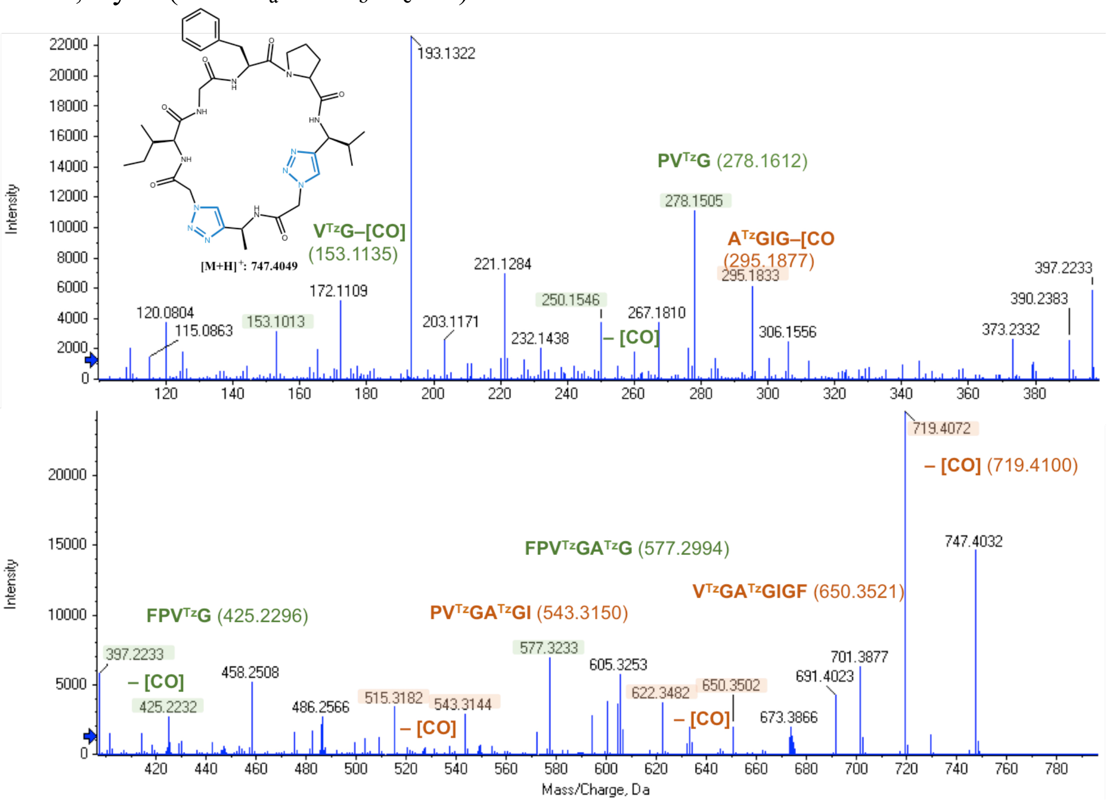


The MS-MS fragmentation data of both cyclic compounds observed in the crude mixture were identical.

**CP135**; Cyclo(-VTzG$_a$ATzG$_b$ITzG$_c$FP-)

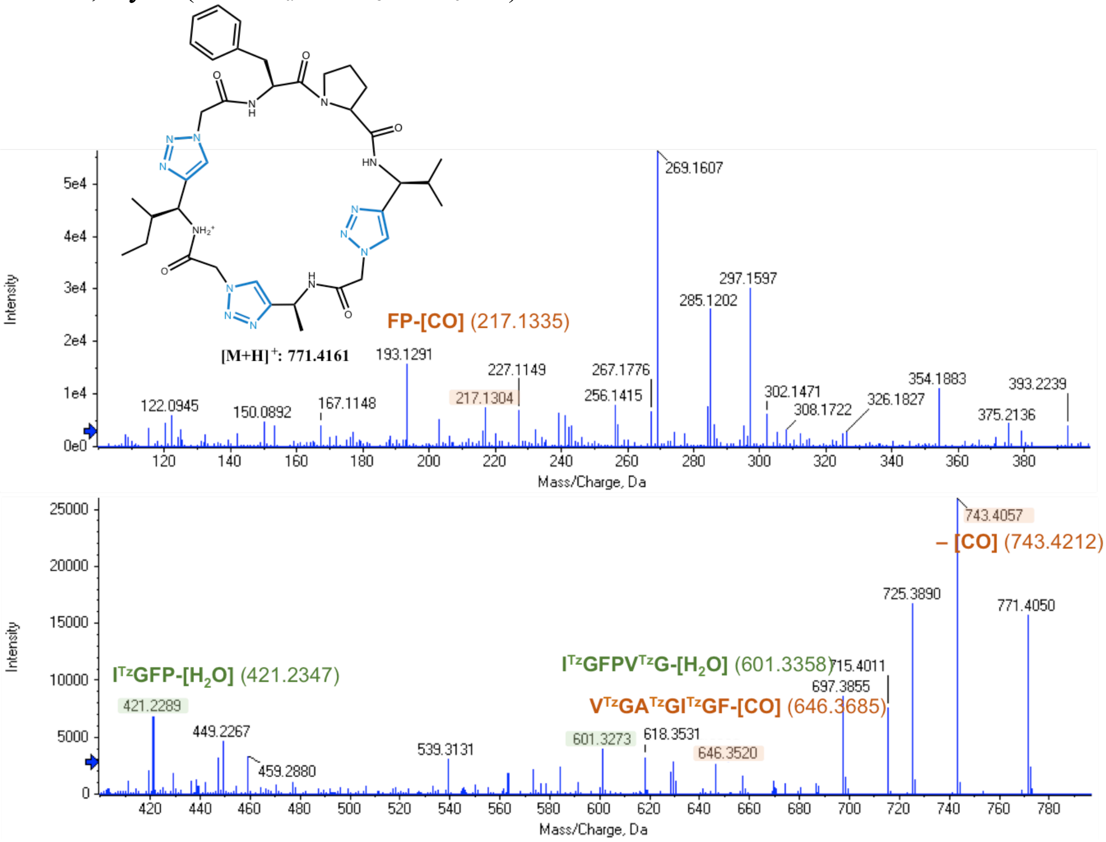


The MS-MS fragmentation data of both cyclic compounds observed in the crude mixture were identical.

# IX. LC-MS traces of crude mixtures of CP3, CP13, and CP135

LC-MS trace of the crude reaction mixture of **CP3** showing two peaks with the same desired mass. Both of these peaks were determined to be the cyclic peptide after analysis by HRMS (Table S2) and MS-MS fragmentation (section VIII).

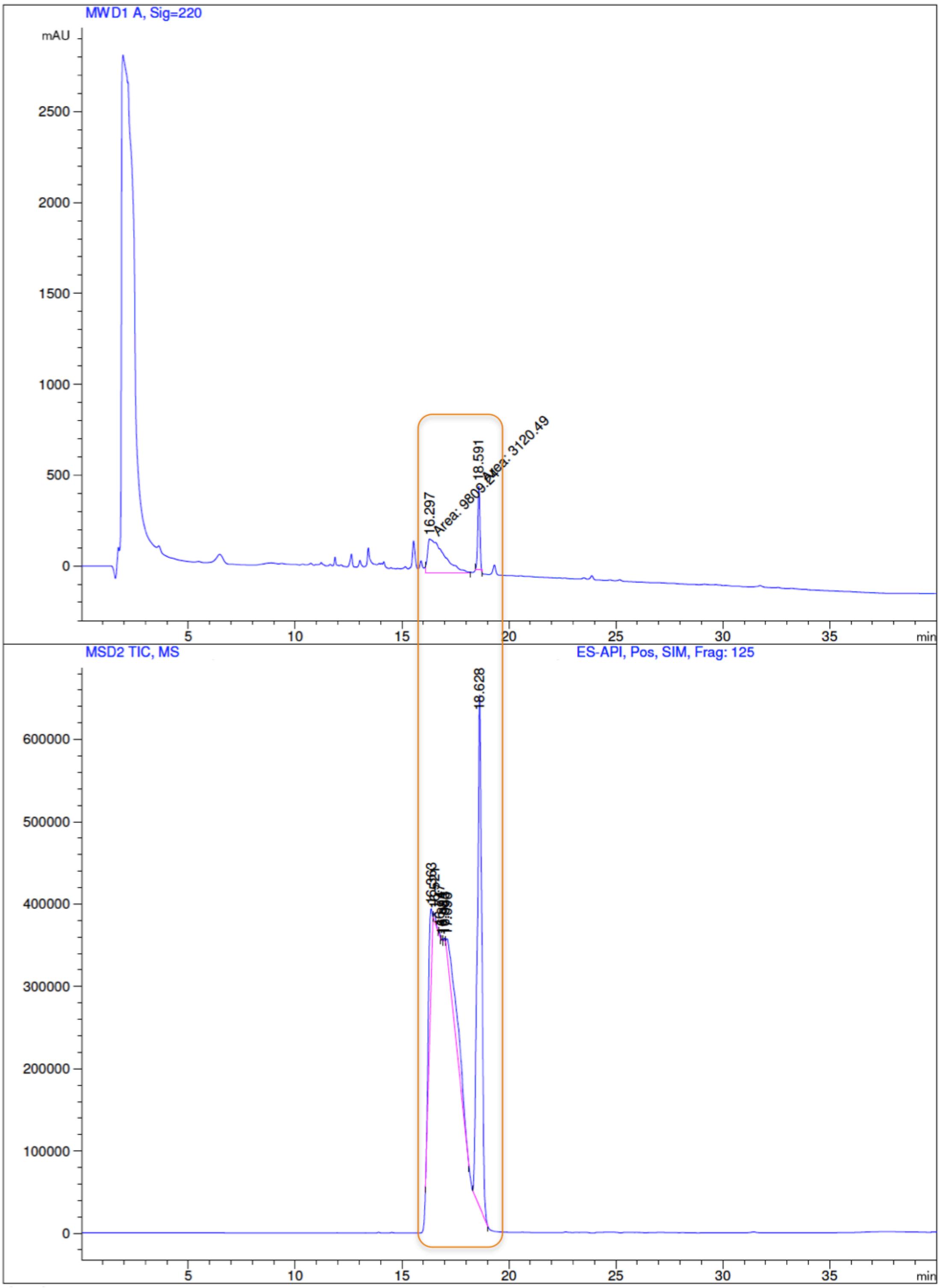

LC-MS trace of the crude reaction mixture of **CP13** showing two peaks with the same desired mass. Both of these peaks were determined to be the cyclic peptide after analysis by HRMS (Table S2) and MS-MS fragmentation (section VIII).

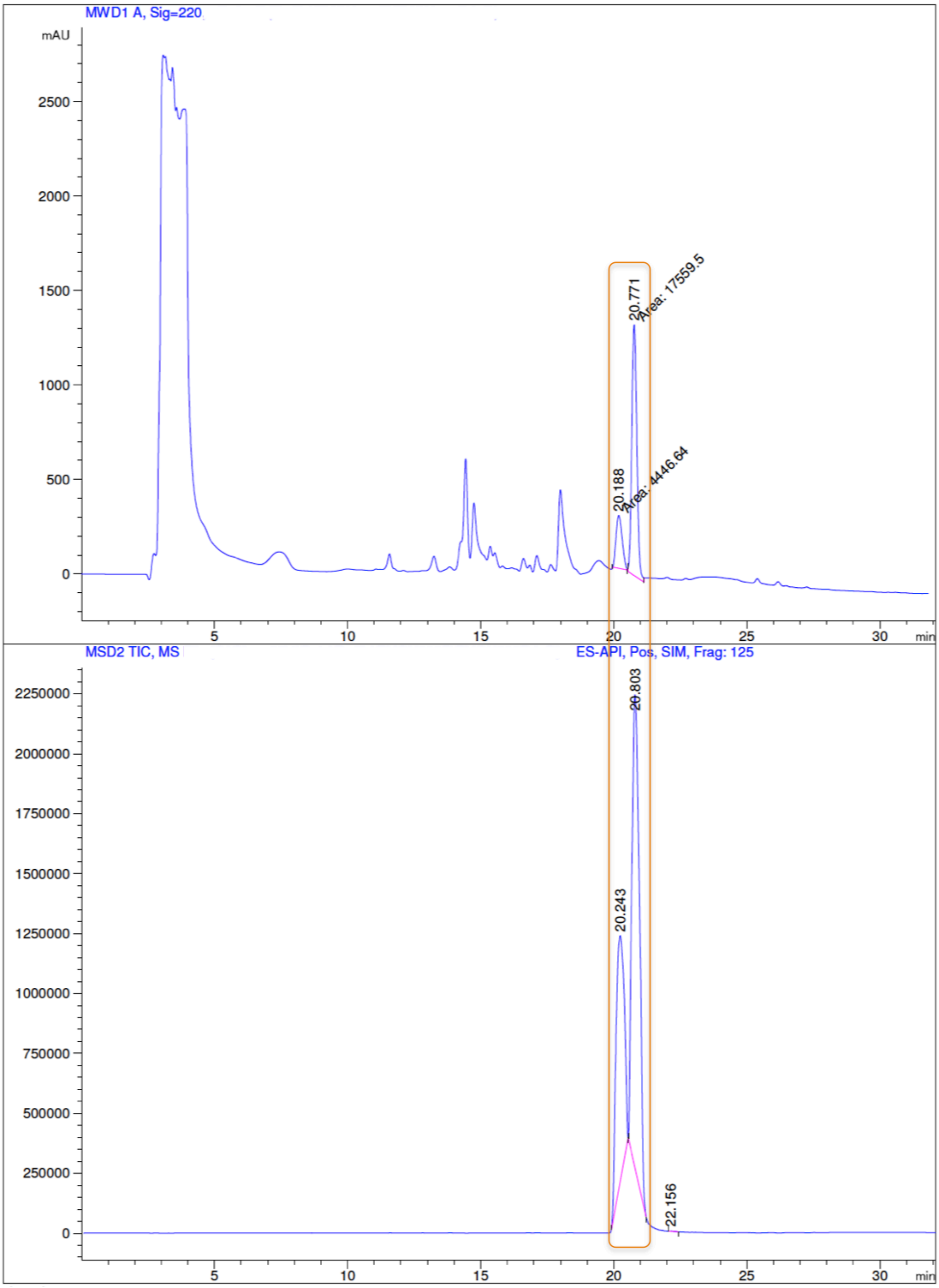

LC-MS trace of the crude reaction mixture of **CP135** showing two peaks with the same desired mass. Both of these peaks were determined to be the cyclic peptide after analysis by HRMS (Table S2) and MS-MS fragmentation (section VIII).

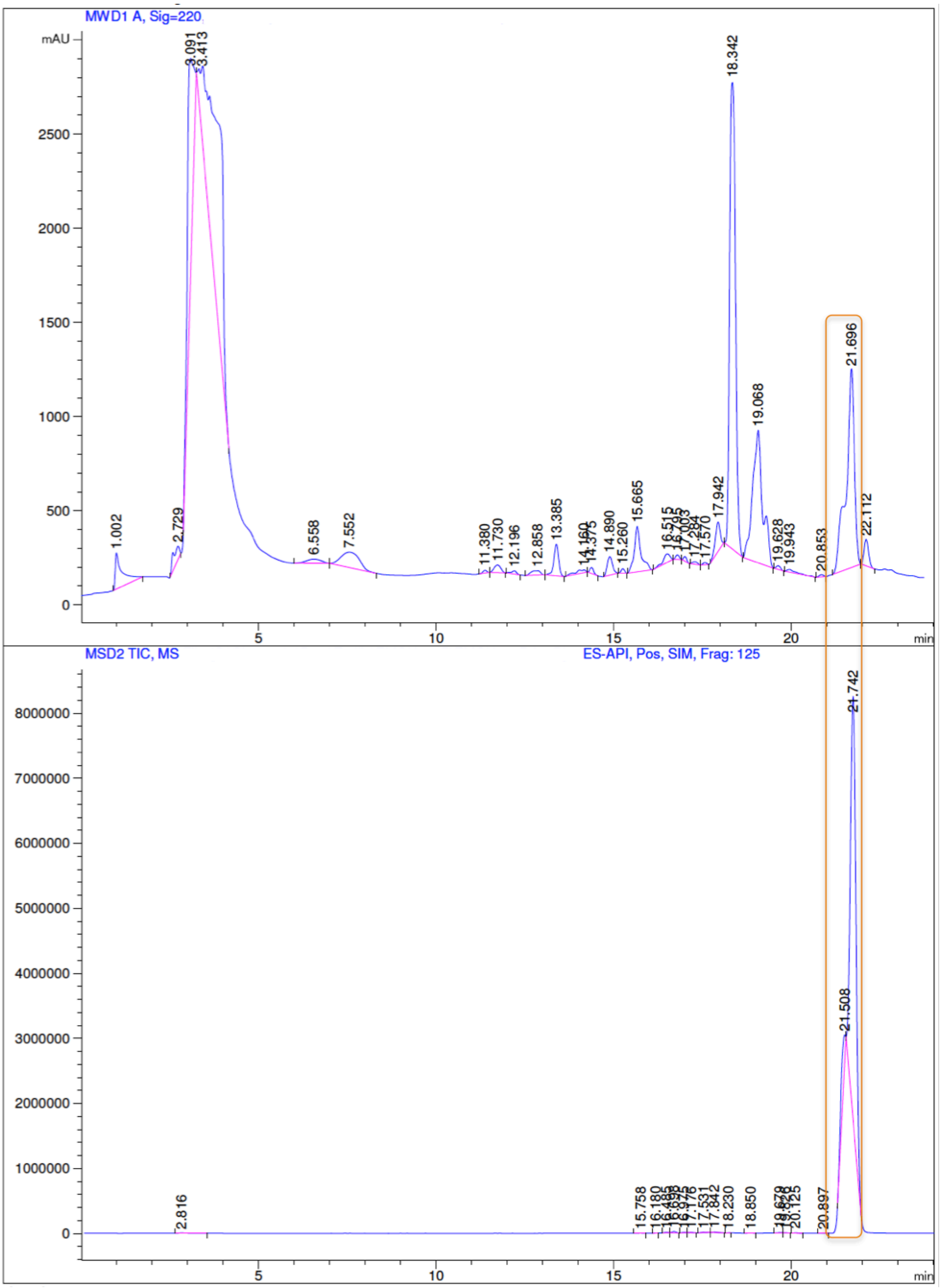

# X. LC-MS traces of final cyclic peptides

For each of the cyclic peptides **CP**, the UV trace at 220 nm obtained by HPLC is complemented by its corresponding LCMS trace at the desired molecular weight (Single Ion Monitoring SIM mode) The HPLC methods used are described in section I.

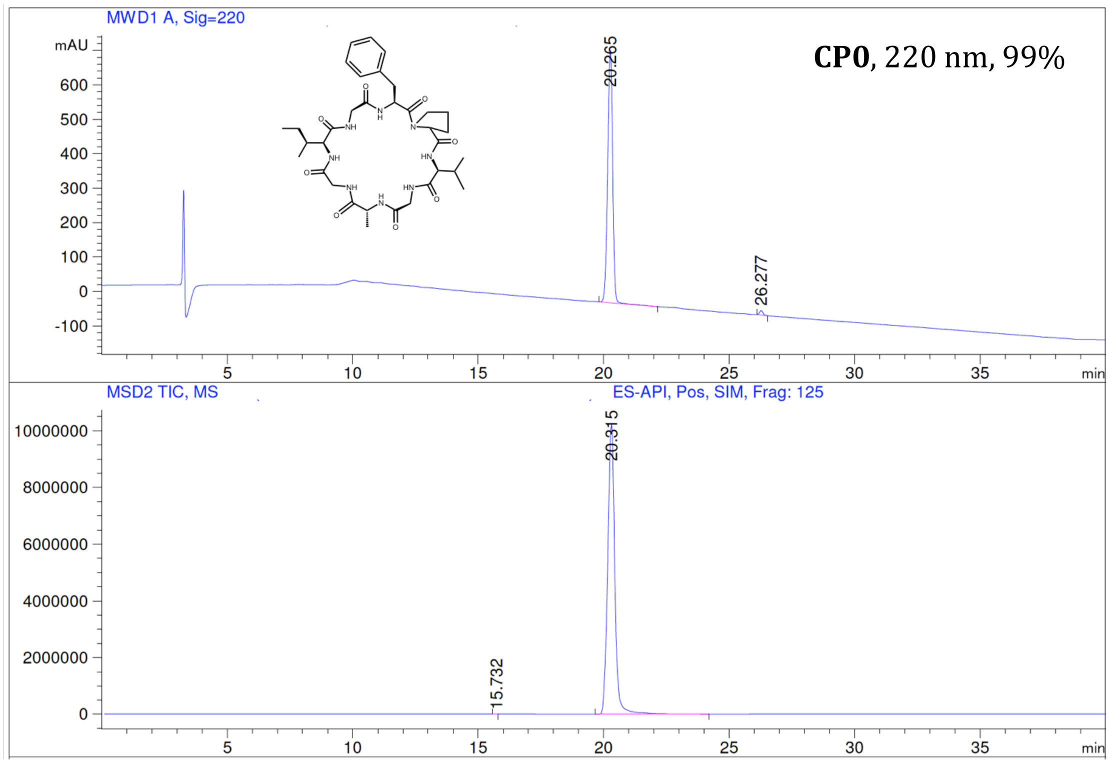


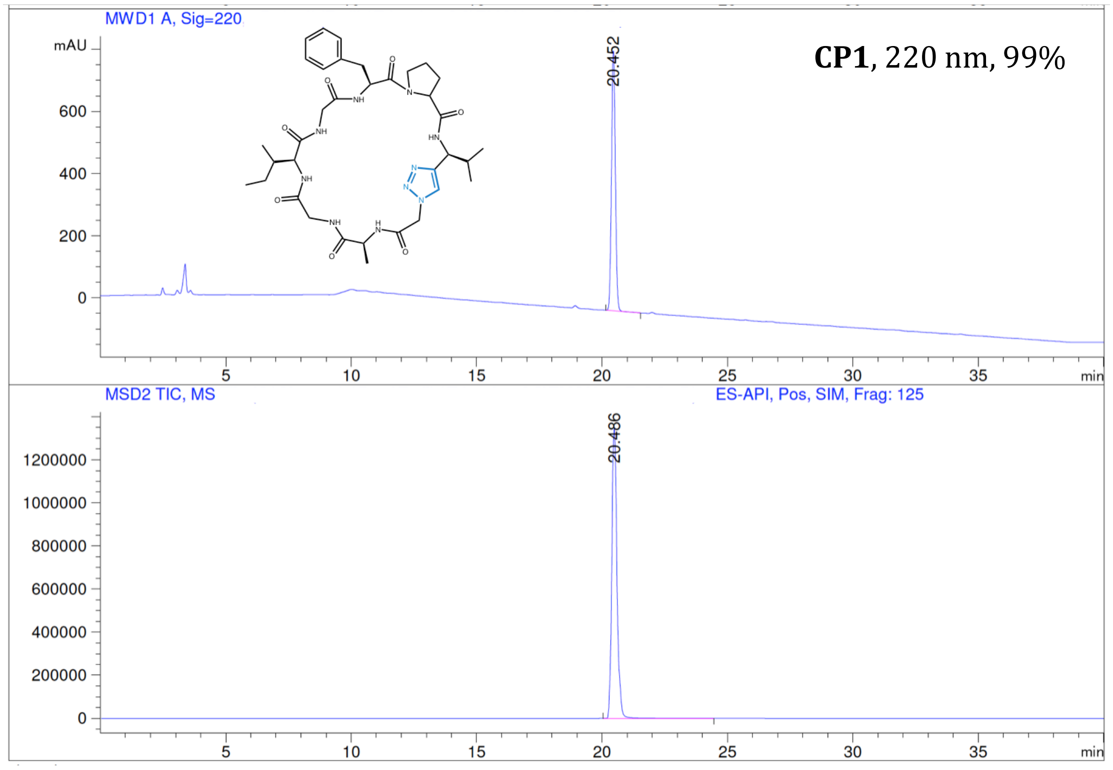

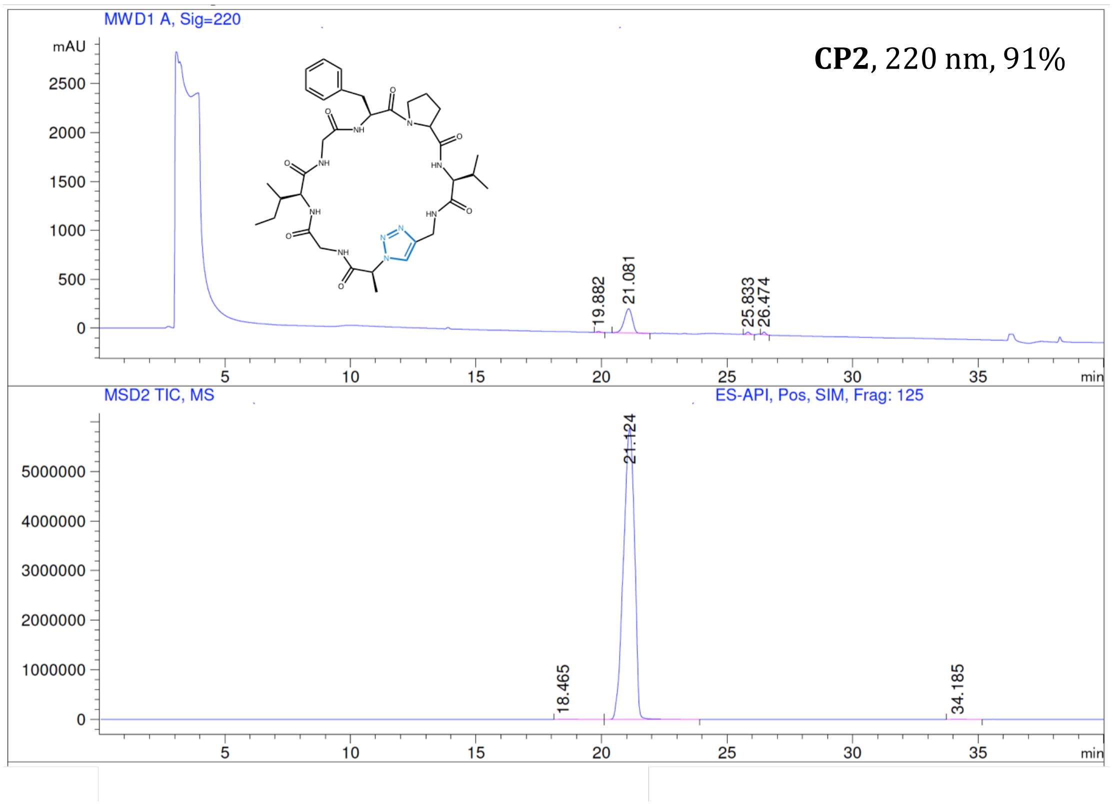
MWD1 A, Sig=220
CP2, 220 nm, 91%
mAU
2500
2000
1500
1000
500
0
19.882
21.081
25.833
26.474
5
10
15
20
25
30
35
min
MSD2 TIC, MS
ES-API, Pos, SIM, Frag: 125
5000000
4000000
3000000
2000000
1000000
0
18.465
21.124
34.185
5
10
15
20
25
30
35
min

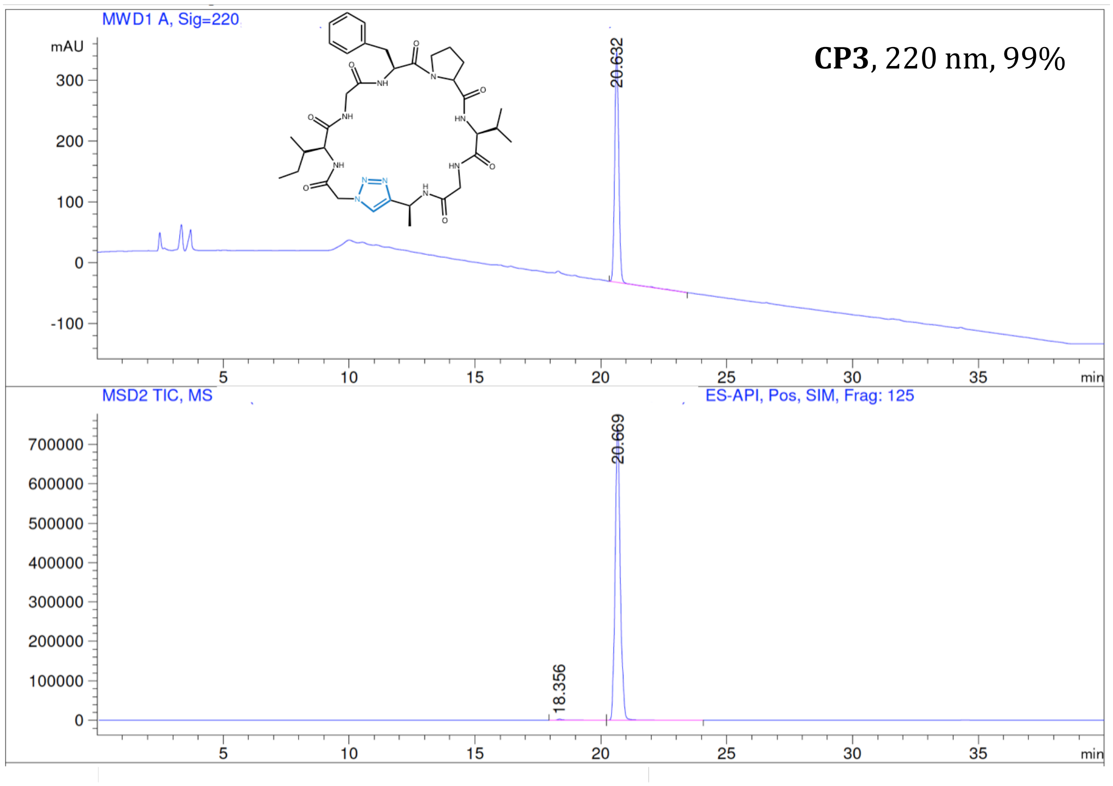
MWD1 A, Sig=220
CP3, 220 nm, 99%
mAU
300
200
100
0
-100
20.632
5
10
15
20
25
30
35
min
MSD2 TIC, MS
ES-API, Pos, SIM, Frag: 125
700000
600000
500000
400000
300000
200000
100000
0
18.356
20.669
5
10
15
20
25
30
35
min

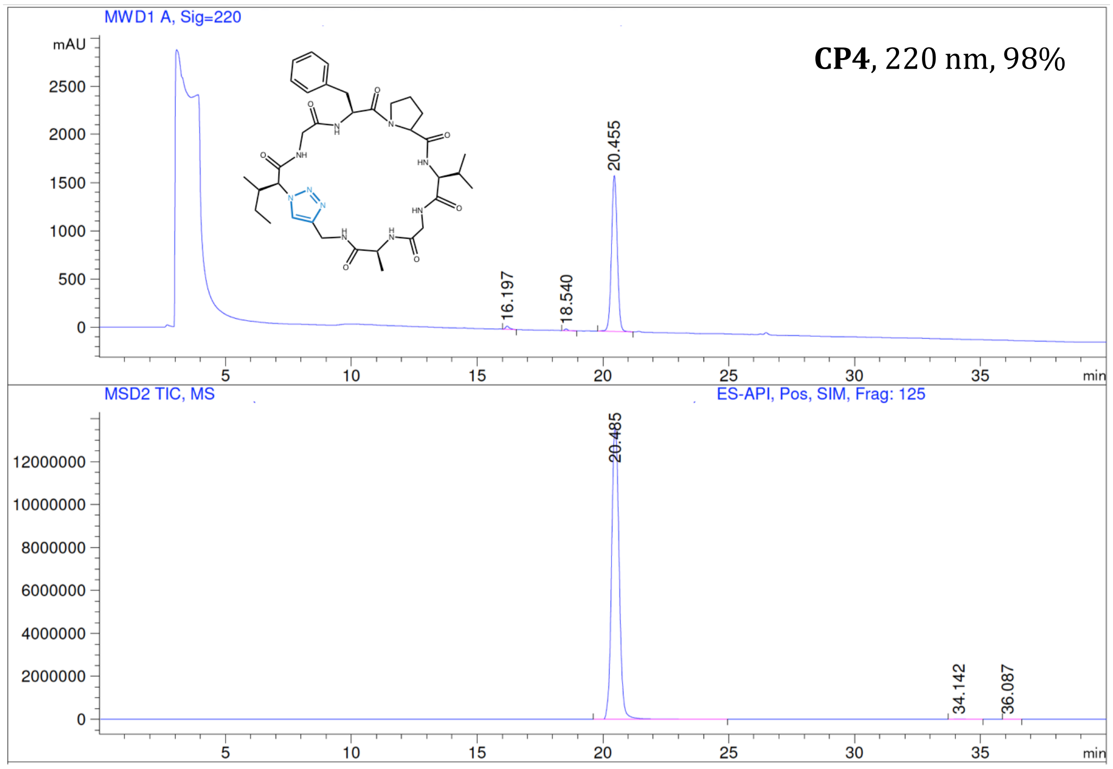
MWD1 A, Sig=220
mAU
CP4, 220 nm, 98%
16.197
18.540
20.455
min
MSD2 TIC, MS
ES-API, Pos, SIM, Frag: 125
20.485
34.142
36.087
min

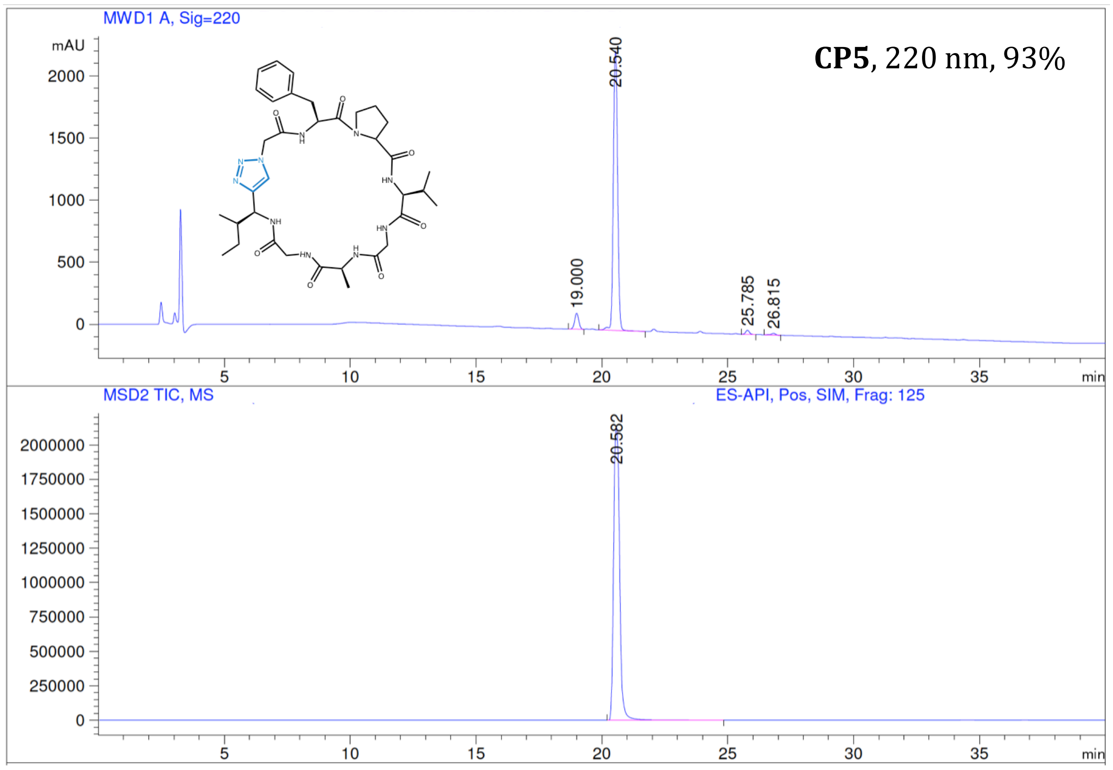
MWD1 A, Sig=220
mAU
CP5, 220 nm, 93%
19.000
20.540
25.785
26.815
min
MSD2 TIC, MS
ES-API, Pos, SIM, Frag: 125
20.582
min

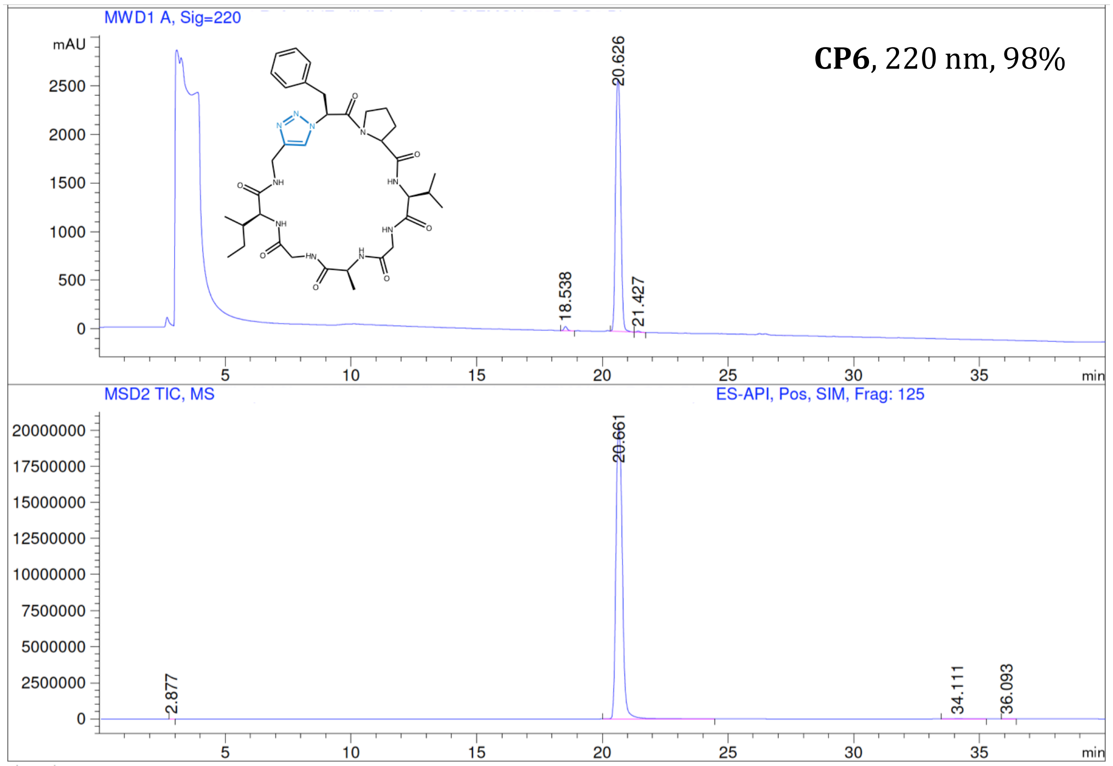
MWD1 A, Sig=220
mAU
**CP6**, 220 nm, 98%
20.626
18.538
21.427
MSD2 TIC, MS
ES-API, Pos, SIM, Frag: 125
20.661
2.877
34.111
36.093
min

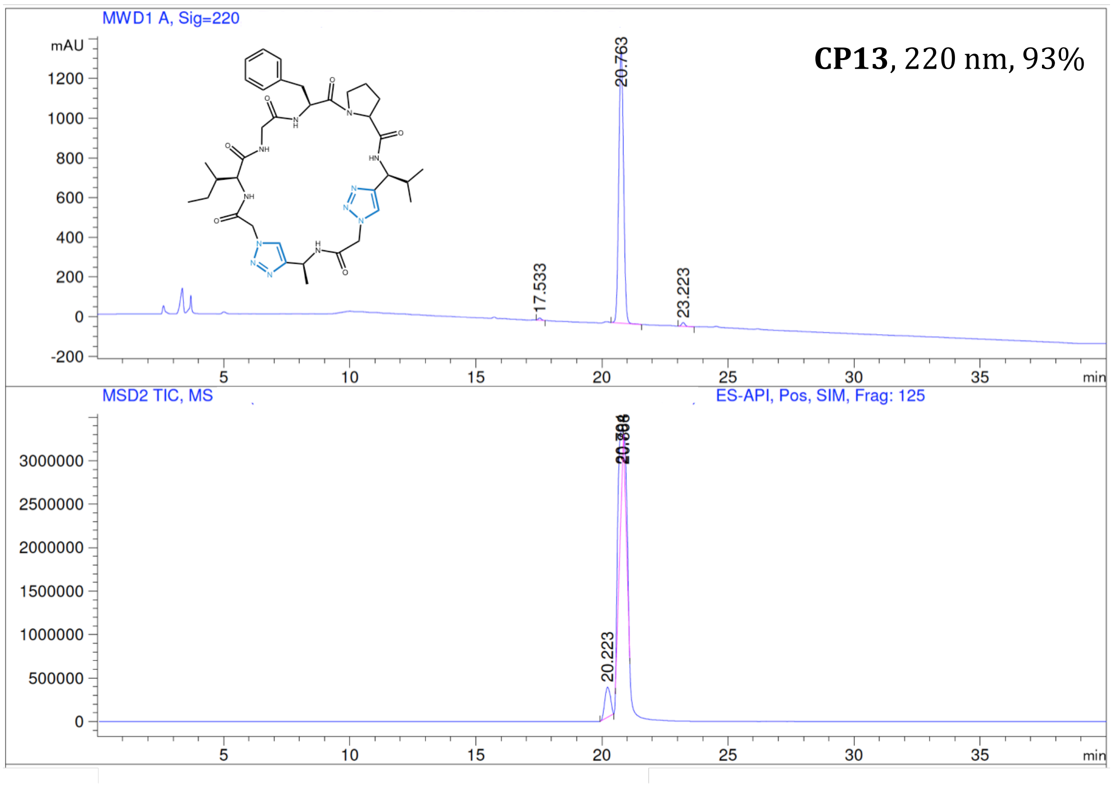
MWD1 A, Sig=220
mAU
**CP13**, 220 nm, 93%
20.763
17.533
23.223
MSD2 TIC, MS
ES-API, Pos, SIM, Frag: 125
20.223
min

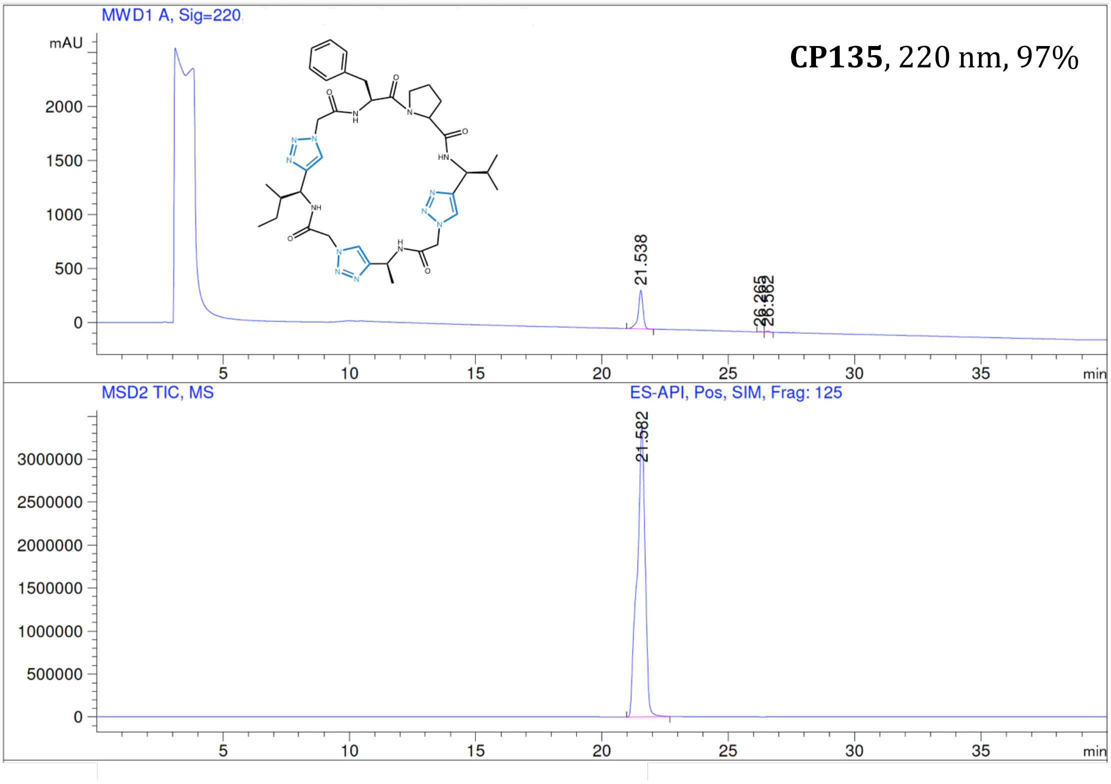


# XI. LC-MS traces of starting peptides

For each of the precursor peptides **P,** the UV trace at 220 nm obtained by HPLC is complemented by its corresponding LCMS trace at the desired molecular weight (Single Ion Monitoring SIM mode). The HPLC methods used are described in section I.

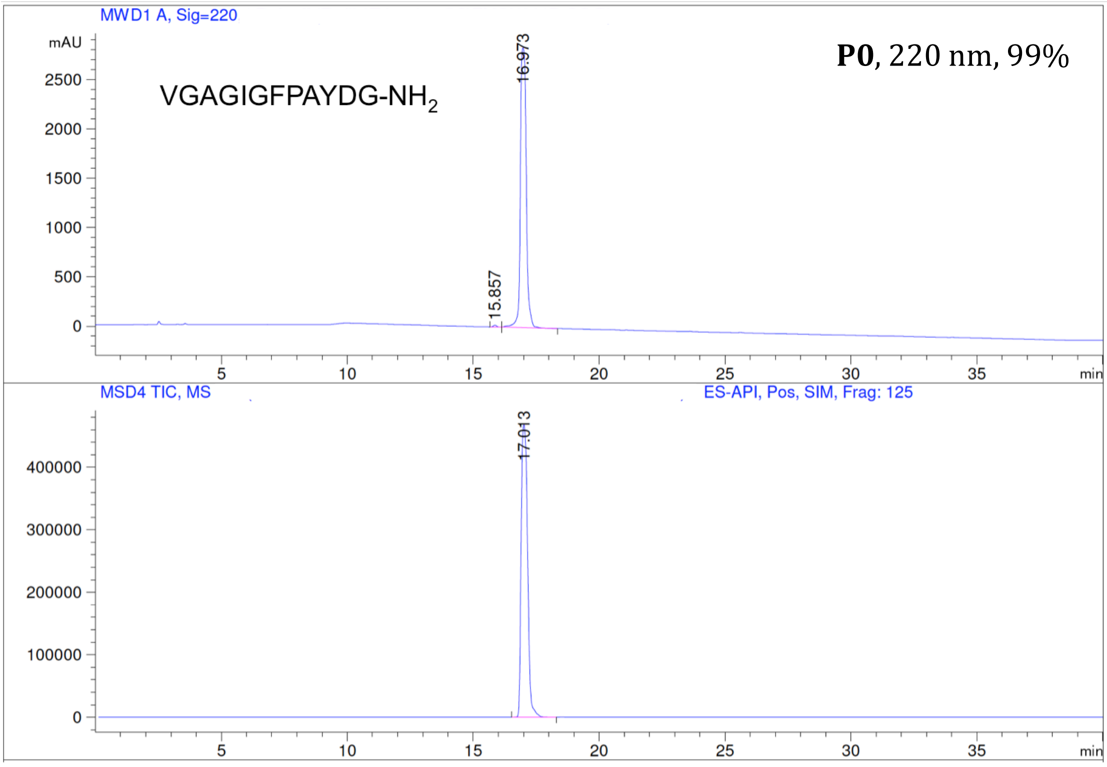

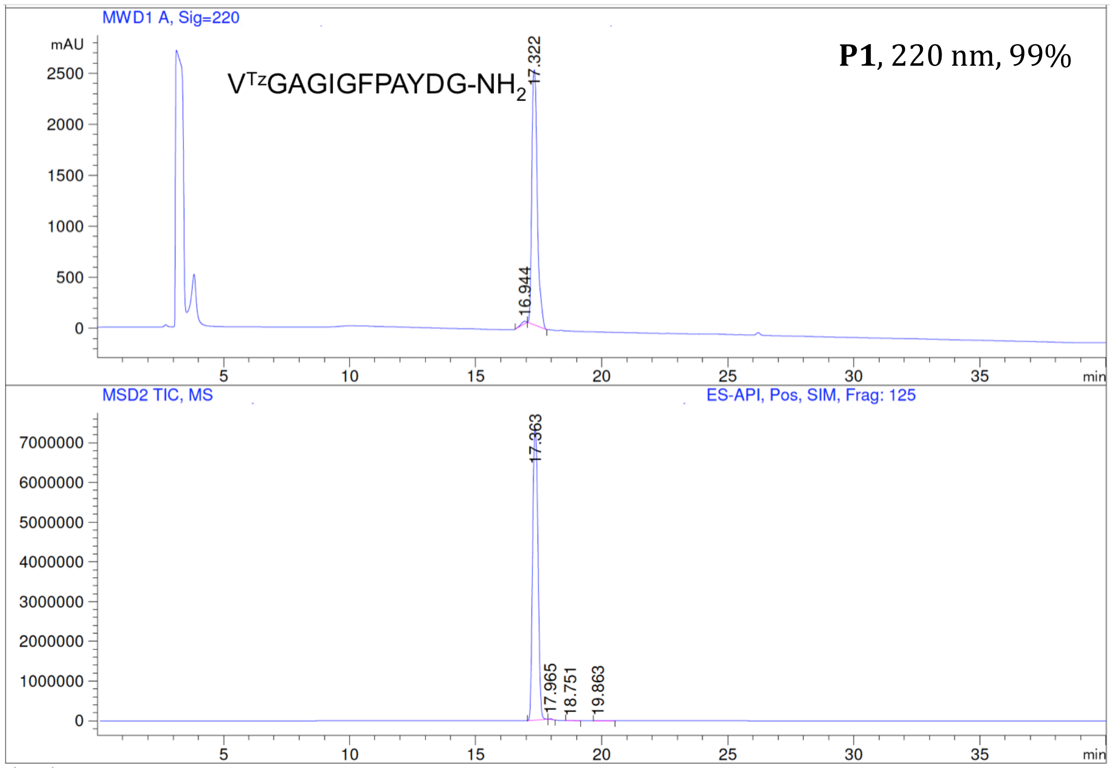

MWD1 A, Sig=220
P1, 220 nm, 99%
VTzGAGIGFPAYDG-NH2
mAU
17.322
16.944
MSD2 TIC, MS
ES-API, Pos, SIM, Frag: 125
17.363
17.965
18.751
19.863
min


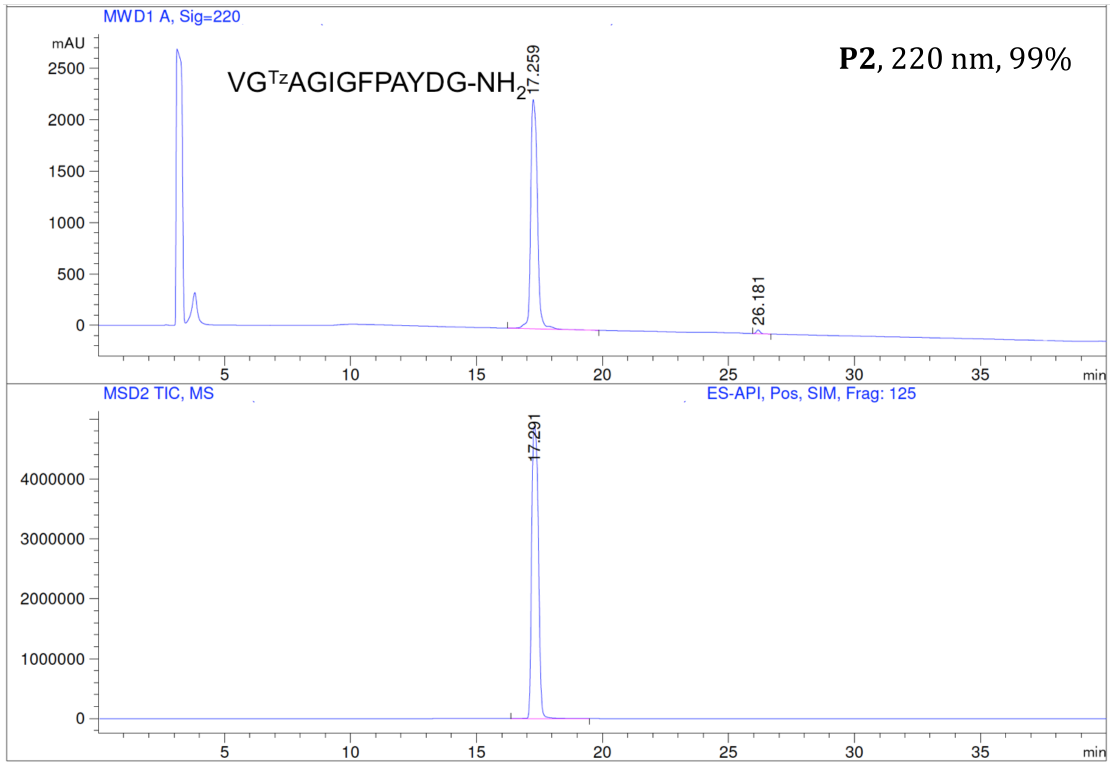

MWD1 A, Sig=220
P2, 220 nm, 99%
VGTzAGIGFPAYDG-NH2
mAU
17.259
26.181
MSD2 TIC, MS
ES-API, Pos, SIM, Frag: 125
17.291
min

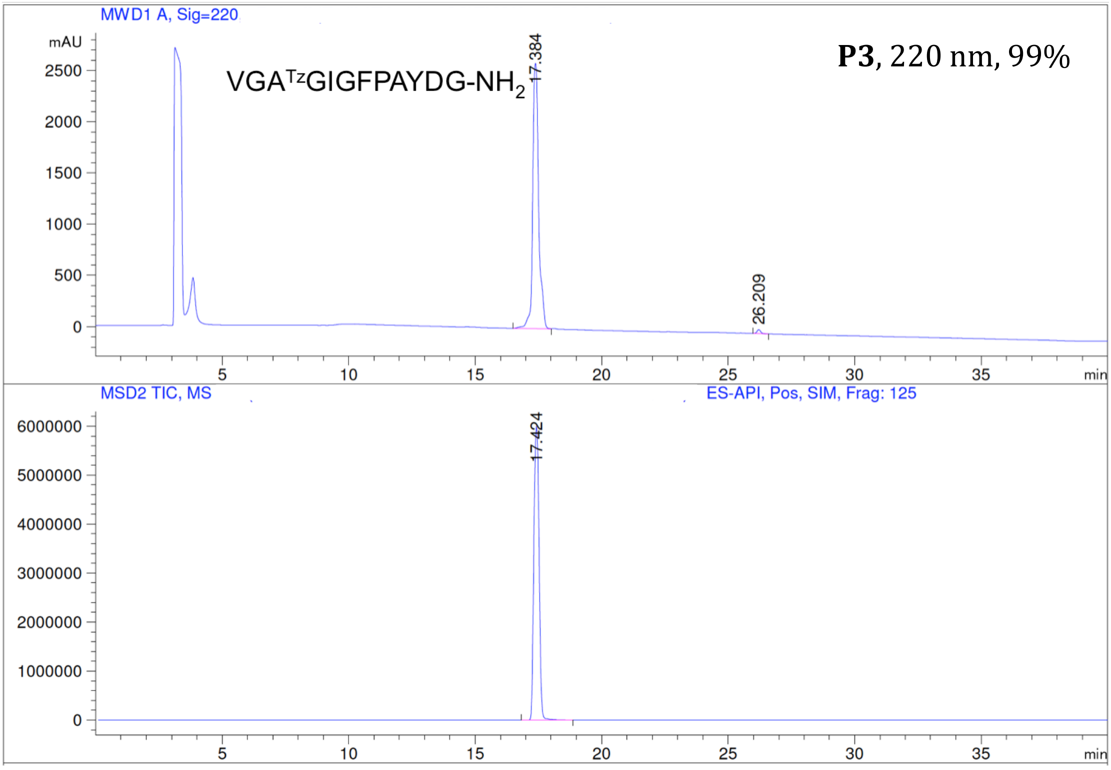
MWD1 A, Sig=220
mAU
VGA^TzGIGFPAYDG-NH2
P3, 220 nm, 99%
17.384
26.209
MSD2 TIC, MS
ES-API, Pos, SIM, Frag: 125
17.424
min

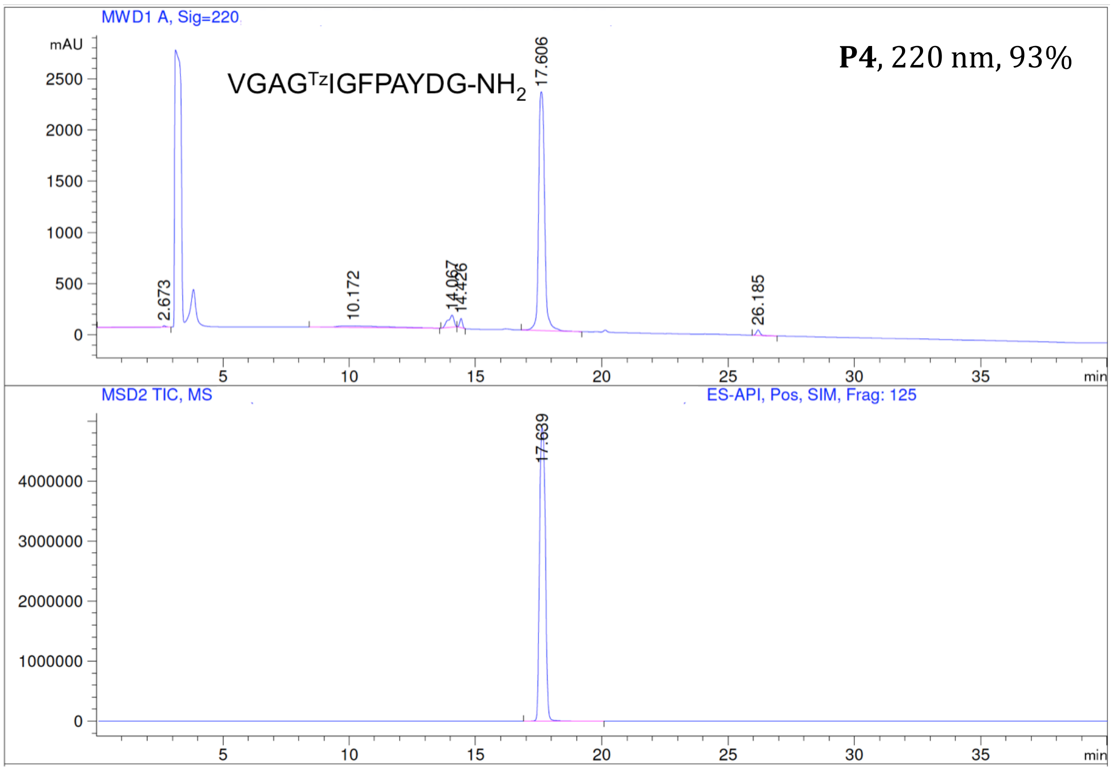
MWD1 A, Sig=220
mAU
VGAG^TzIGFPAYDG-NH2
P4, 220 nm, 93%
2.673
10.172
14.067
14.426
17.606
26.185
MSD2 TIC, MS
ES-API, Pos, SIM, Frag: 125
17.639
min

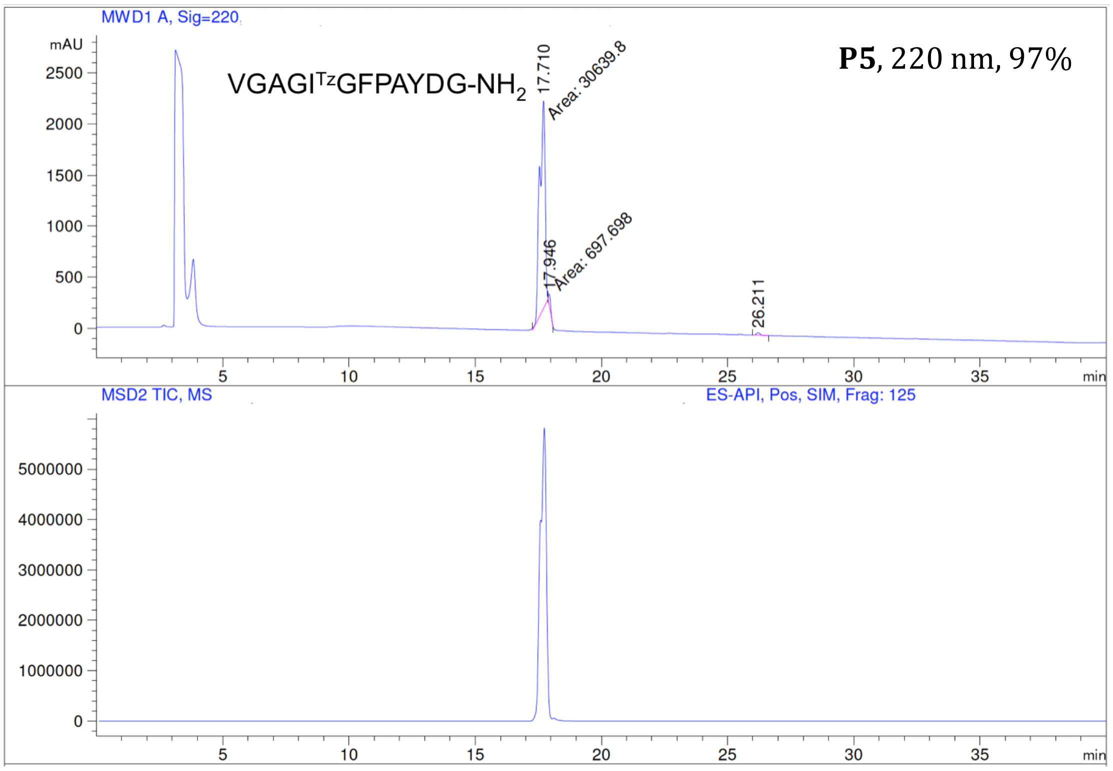
MWD1 A, Sig=220
mAU
VGAGI^TzGFPAYDG-NH2
17.710
Area: 30639.8
17.946
Area: 697.698
26.211
P5, 220 nm, 97%
MSD2 TIC, MS
ES-API, Pos, SIM, Frag: 125
min

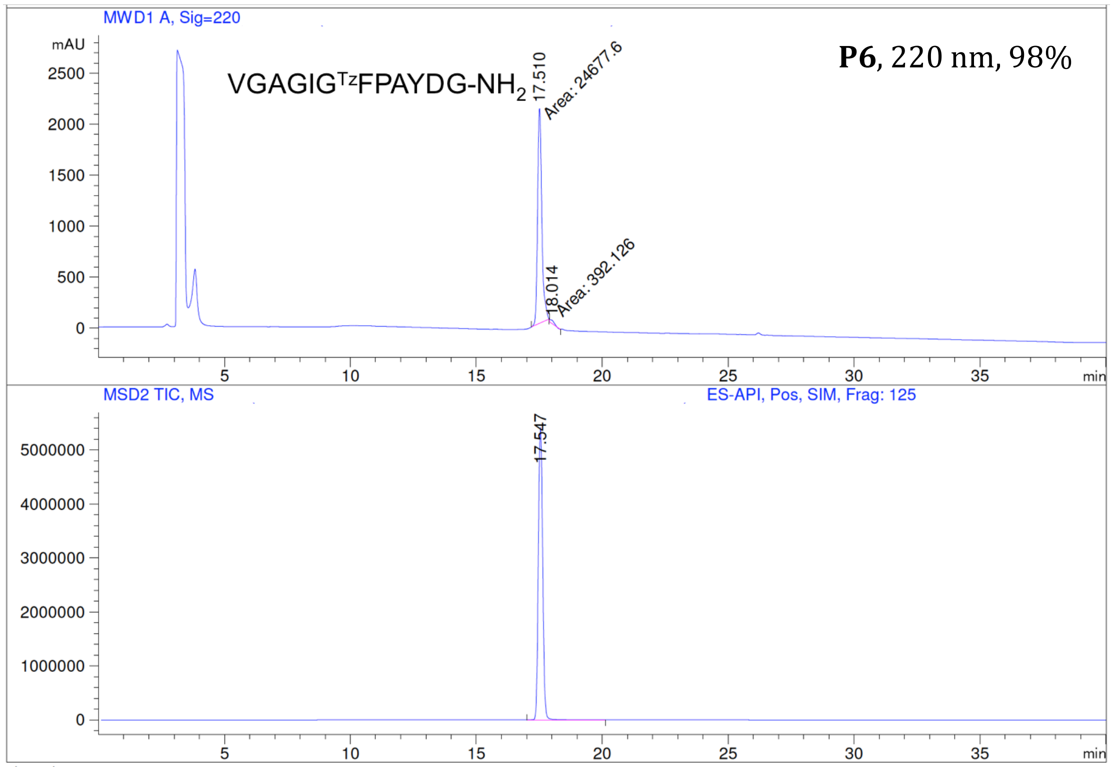
MWD1 A, Sig=220
mAU
VGAGIG^TzFPAYDG-NH2
17.510
Area: 24677.6
18.014
Area: 392.126
P6, 220 nm, 98%
MSD2 TIC, MS
ES-API, Pos, SIM, Frag: 125
17.547
min

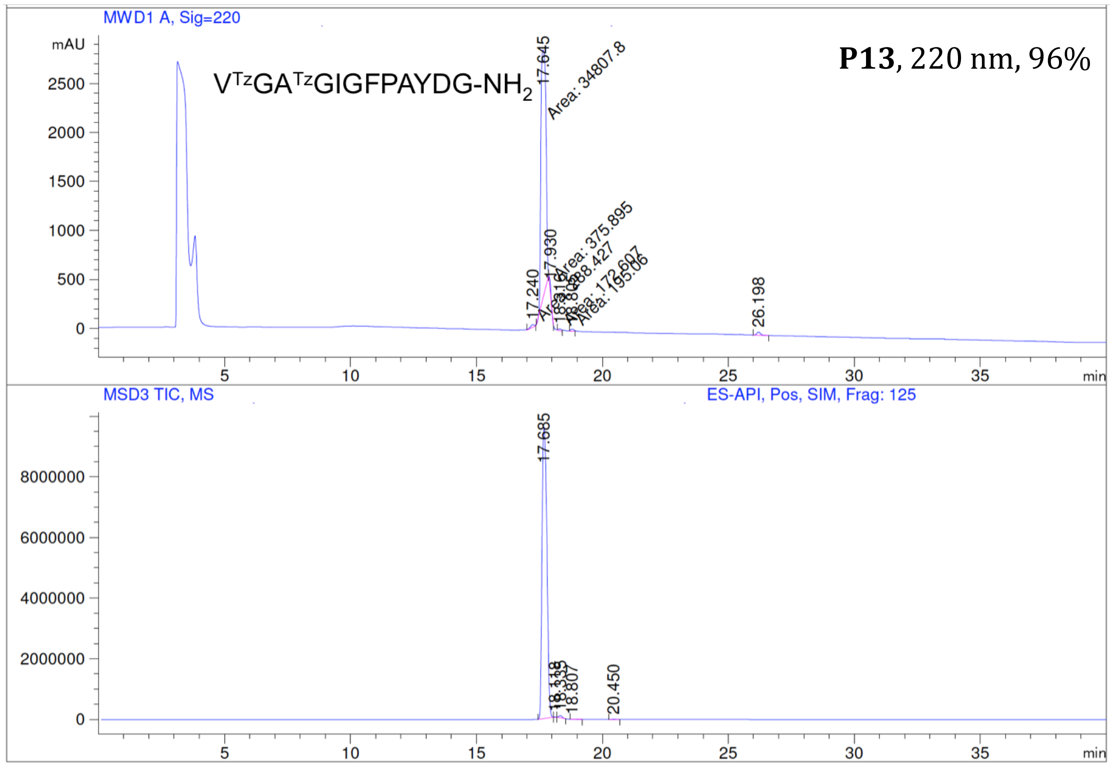
MWD1 A, Sig=220
mAU
VTzGATzGIGFPAYDG-NH2
P13, 220 nm, 96%
17.645
Area: 34807.8
17.930
17.240
26.198
MSD3 TIC, MS
ES-API, Pos, SIM, Frag: 125
17.685
18.807
20.450
min

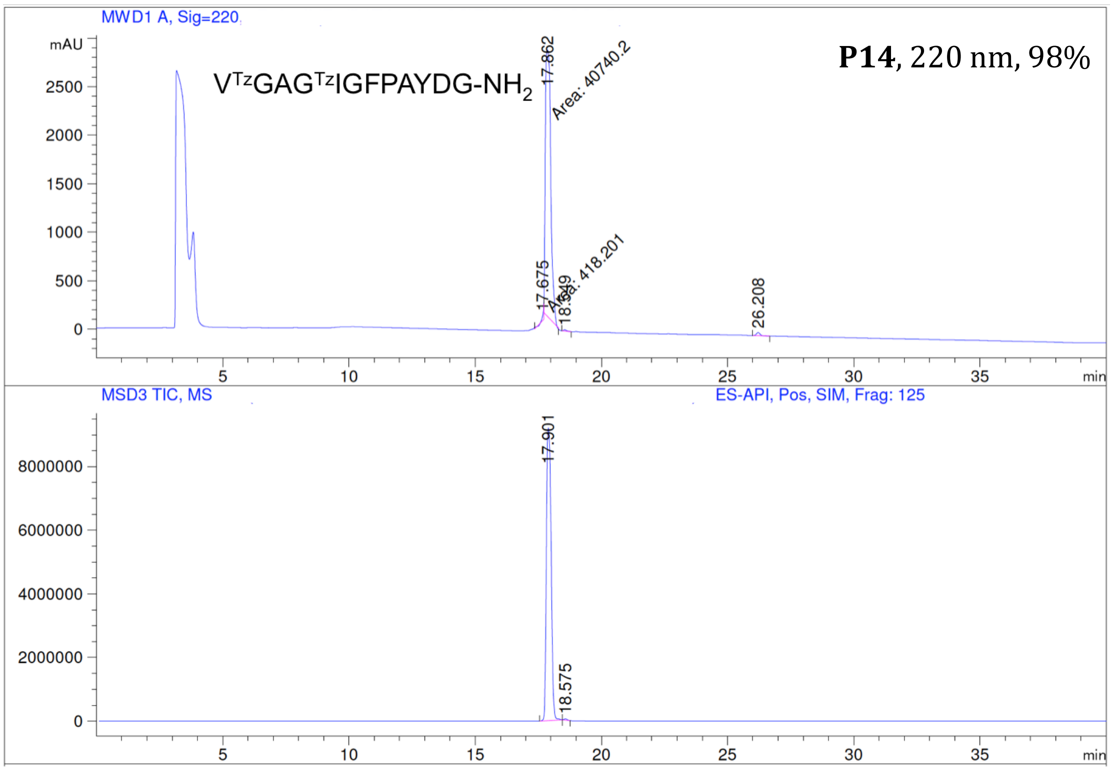
MWD1 A, Sig=220
mAU
VTzGAGTzIGFPAYDG-NH2
P14, 220 nm, 98%
17.862
Area: 40740.2
17.675
Area: 418.201
26.208
MSD3 TIC, MS
ES-API, Pos, SIM, Frag: 125
17.901
18.575
min

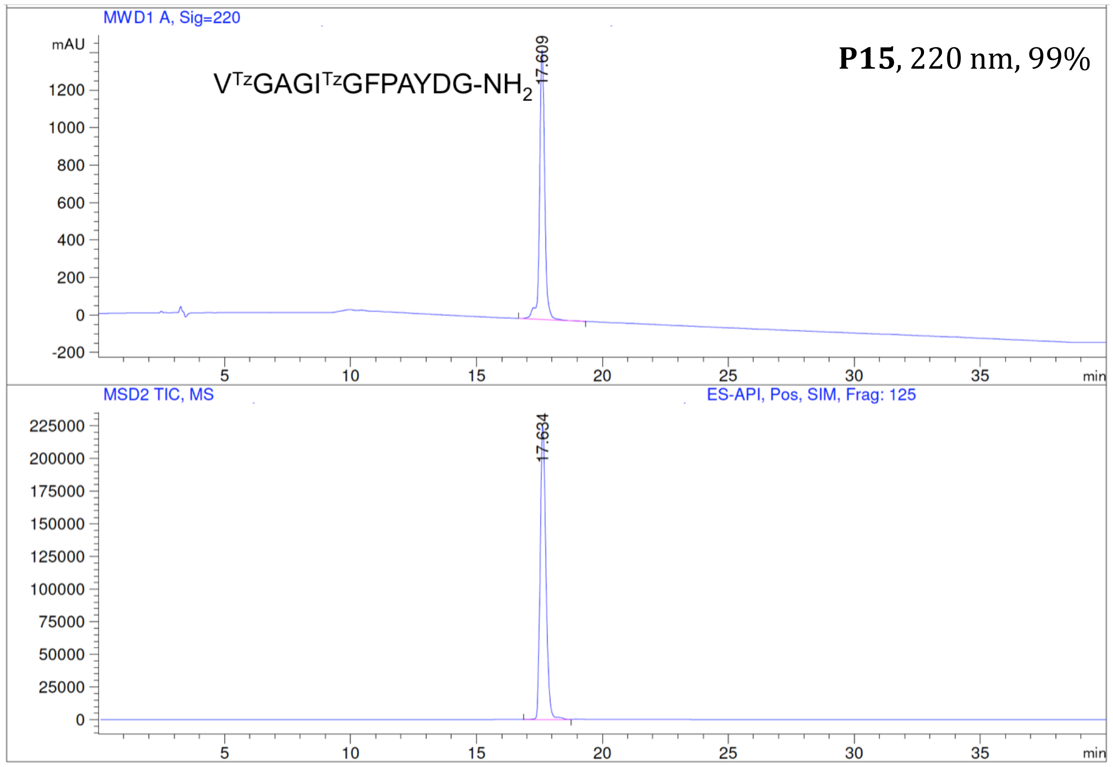
MWD1 A, Sig=220
mAU
P15, 220 nm, 99%
$V^{Tz}GAGI^{Tz}GFPAYDG-NH_2$
17.609
1200
1000
800
600
400
200
0
-200
5
10
15
20
25
30
35
min
MSD2 TIC, MS
ES-API, Pos, SIM, Frag: 125
17.634
225000
200000
175000
150000
125000
100000
75000
50000
25000
0

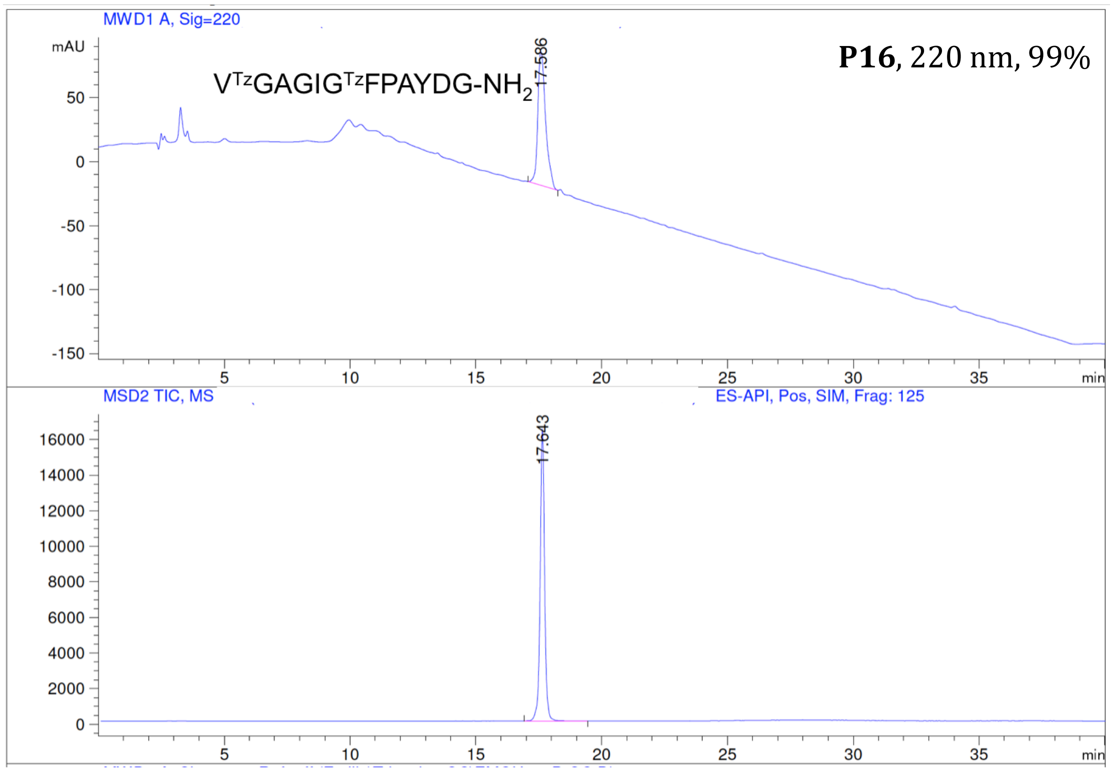
MWD1 A, Sig=220
mAU
P16, 220 nm, 99%
$V^{Tz}GAGIG^{Tz}FPAYDG-NH_2$
17.586
50
0
-50
-100
-150
5
10
15
20
25
30
35
min
MSD2 TIC, MS
ES-API, Pos, SIM, Frag: 125
17.643
16000
14000
12000
10000
8000
6000
4000
2000
0

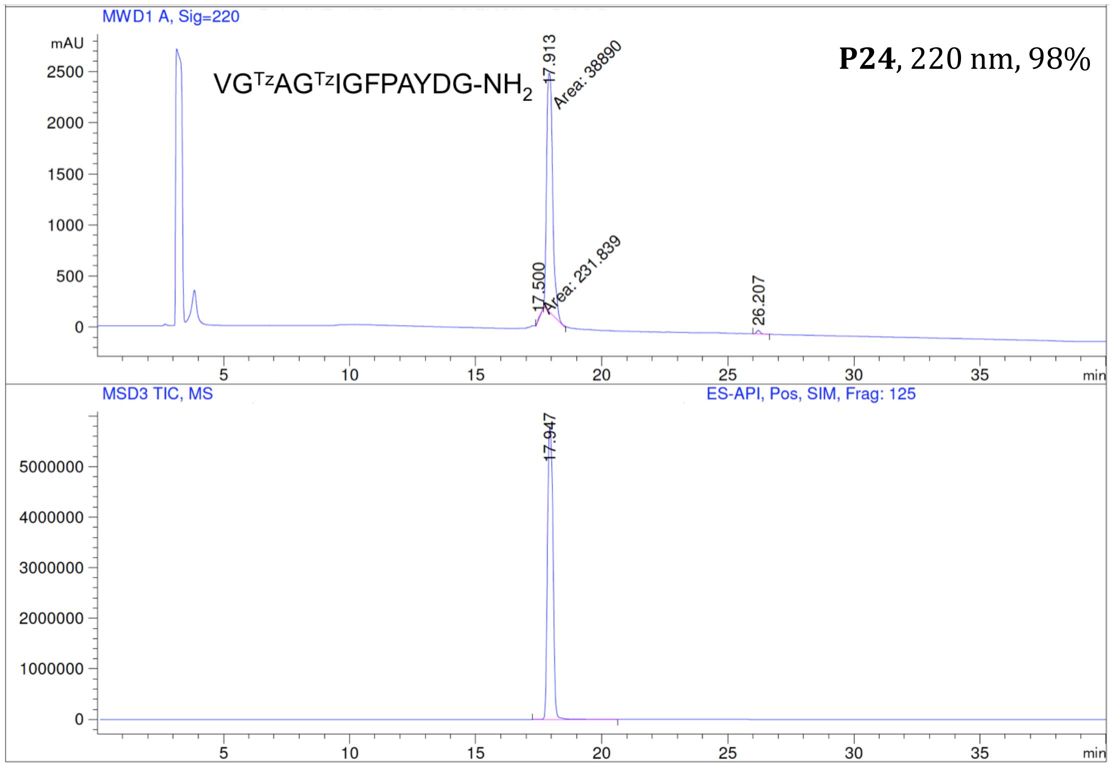


**P24**, 220 nm, 98%

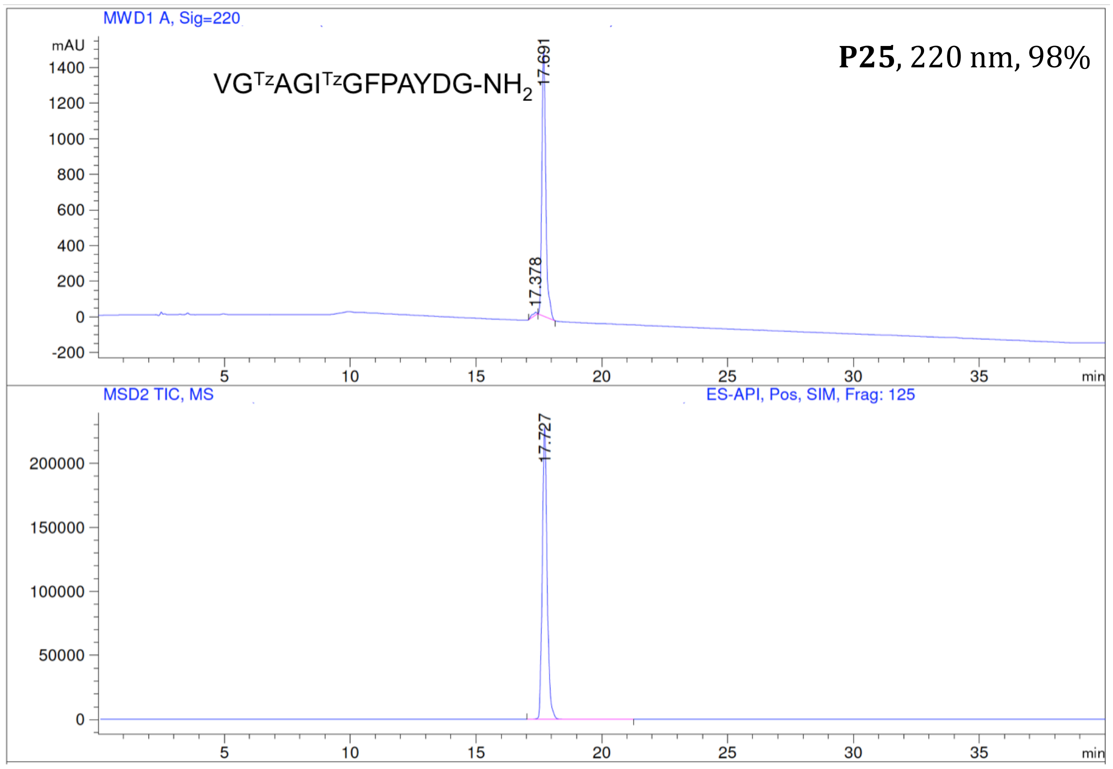


**P25**, 220 nm, 98%

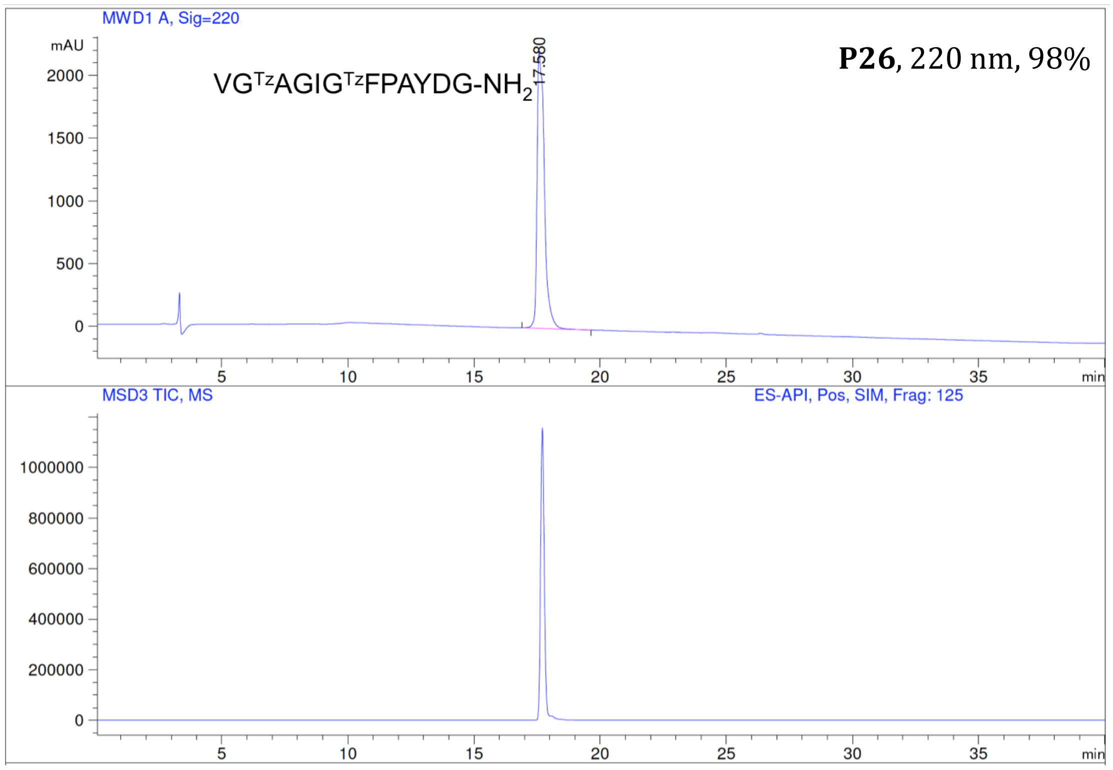
MWD1 A, Sig=220
mAU
VG$^{Tz}$AGIG$^{Tz}$FPAYDG-NH$_2$
17.580
**P26**, 220 nm, 98%
MSD3 TIC, MS
ES-API, Pos, SIM, Frag: 125
min

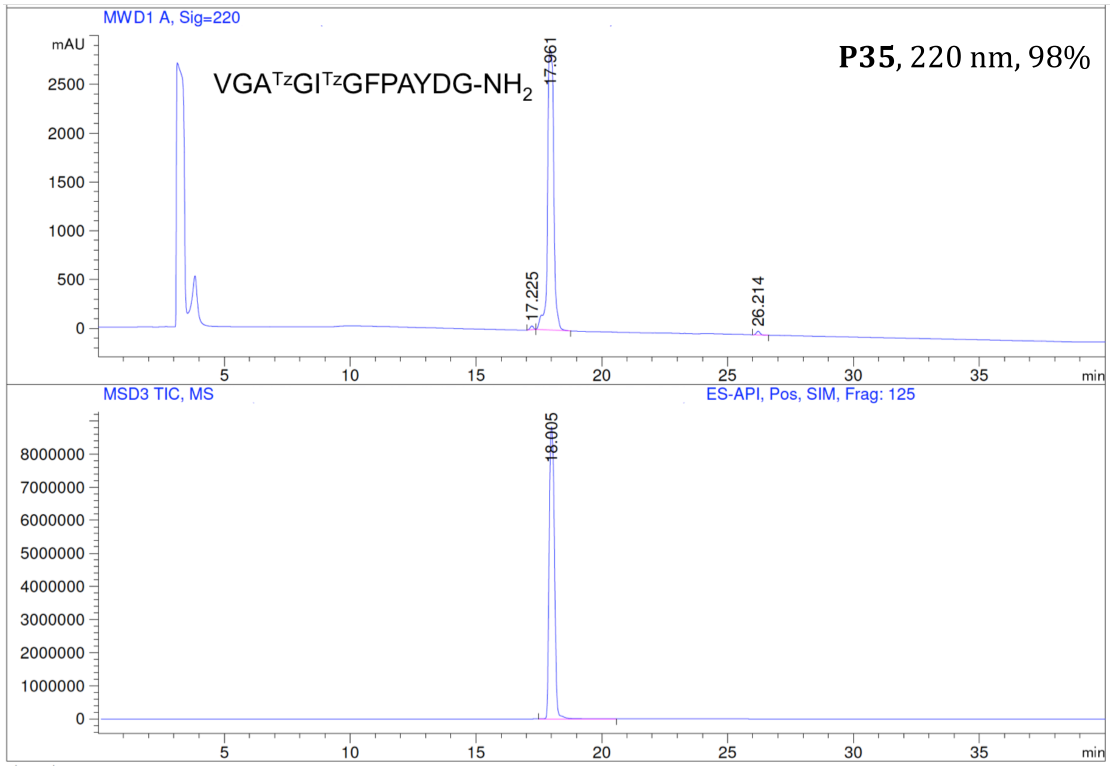
MWD1 A, Sig=220
mAU
VGA$^{Tz}$GI$^{Tz}$GFPAYDG-NH$_2$
17.961
17.225
26.214
**P35**, 220 nm, 98%
MSD3 TIC, MS
ES-API, Pos, SIM, Frag: 125
18.005
min

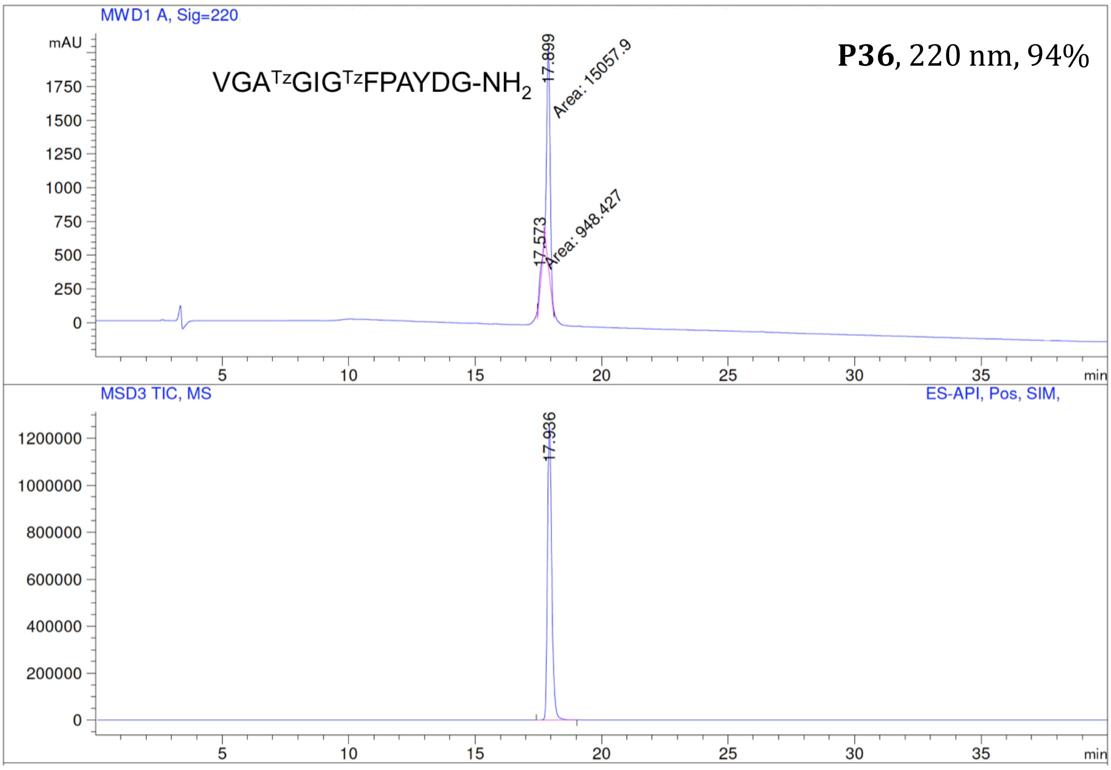
MWD1 A, Sig=220
mAU
VGA$^{Tz}$GIG$^{Tz}$FPAYDG-NH$_2$
17.899
Area: 15057.9
17.573
Area: 948.427
**P36**, 220 nm, 94%
MSD3 TIC, MS
ES-API, Pos, SIM,
17.936
min

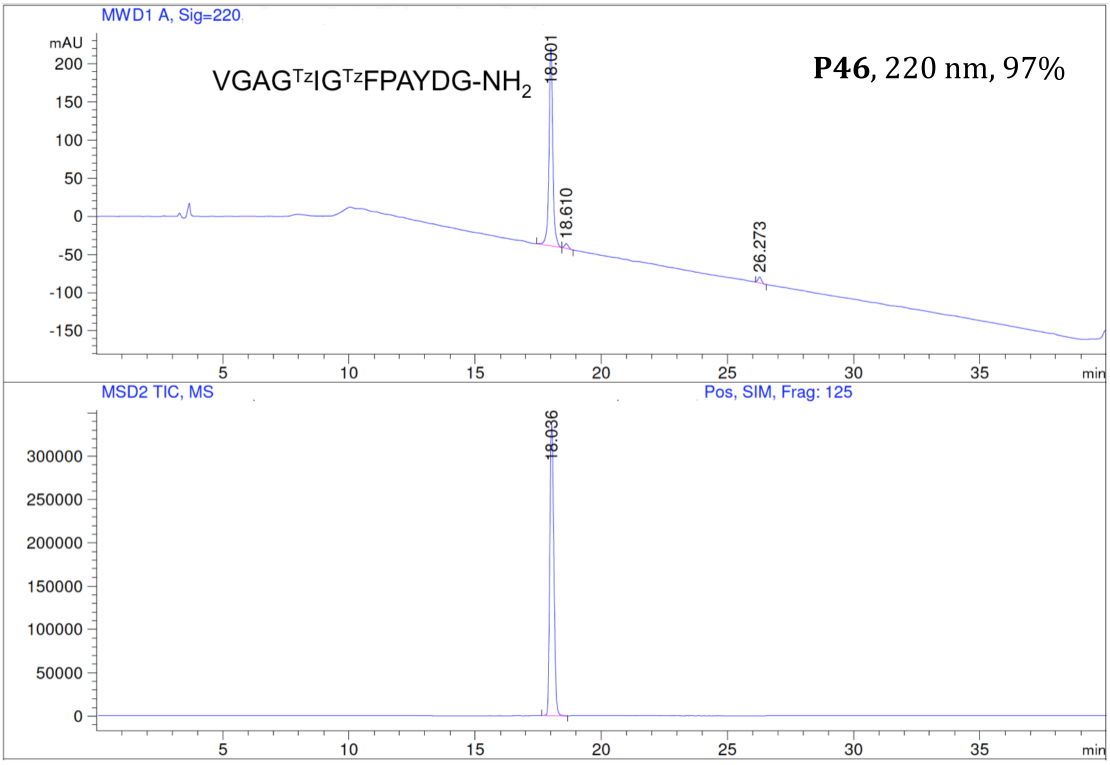
MWD1 A, Sig=220
mAU
VGAG$^{Tz}$IG$^{Tz}$FPAYDG-NH$_2$
18.001
18.610
26.273
**P46**, 220 nm, 97%
MSD2 TIC, MS
Pos, SIM, Frag: 125
18.036
min

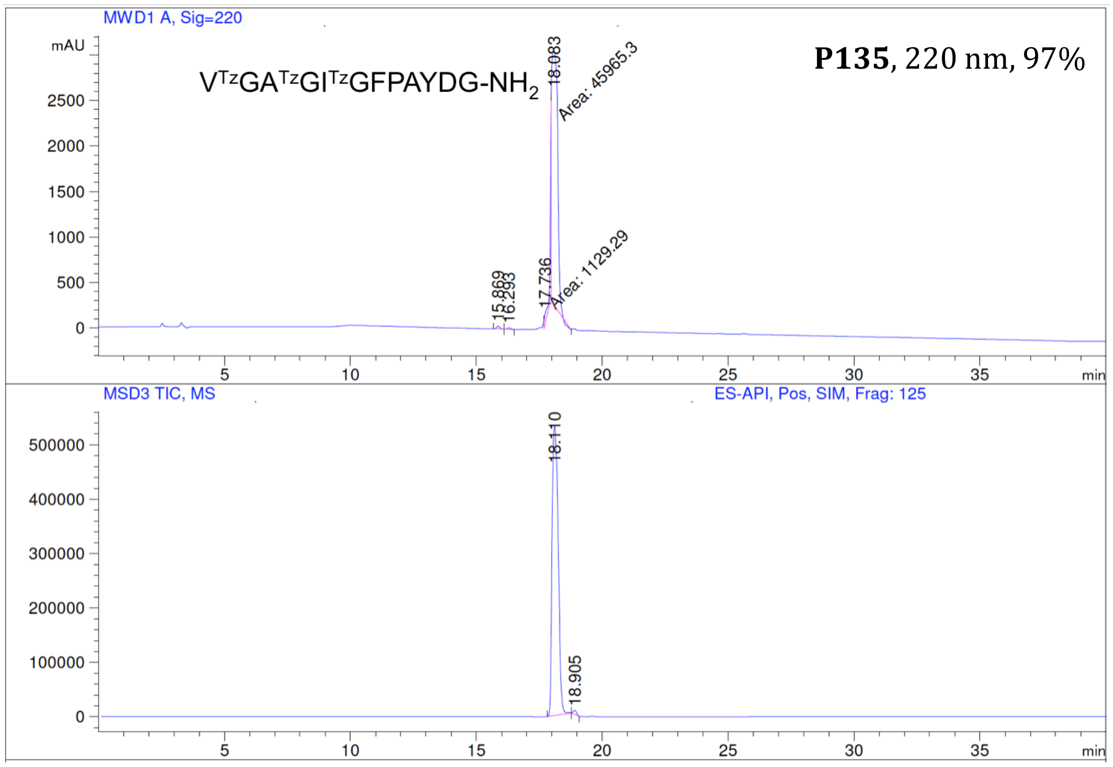
MWD1 A, Sig=220
mAU
V$^{Tz}$GA$^{Tz}$GI$^{Tz}$GFPAYDG-NH$_2$
**P135**, 220 nm, 97%
18.083
Area: 45965.3
15.869
16.293
17.736
Area: 1129.29
2500
2000
1500
1000
500
0
5
10
15
20
25
30
35
min
MSD3 TIC, MS
ES-API, Pos, SIM, Frag: 125
18.110
18.905
500000
400000
300000
200000
100000
0
5
10
15
20
25
30
35
min

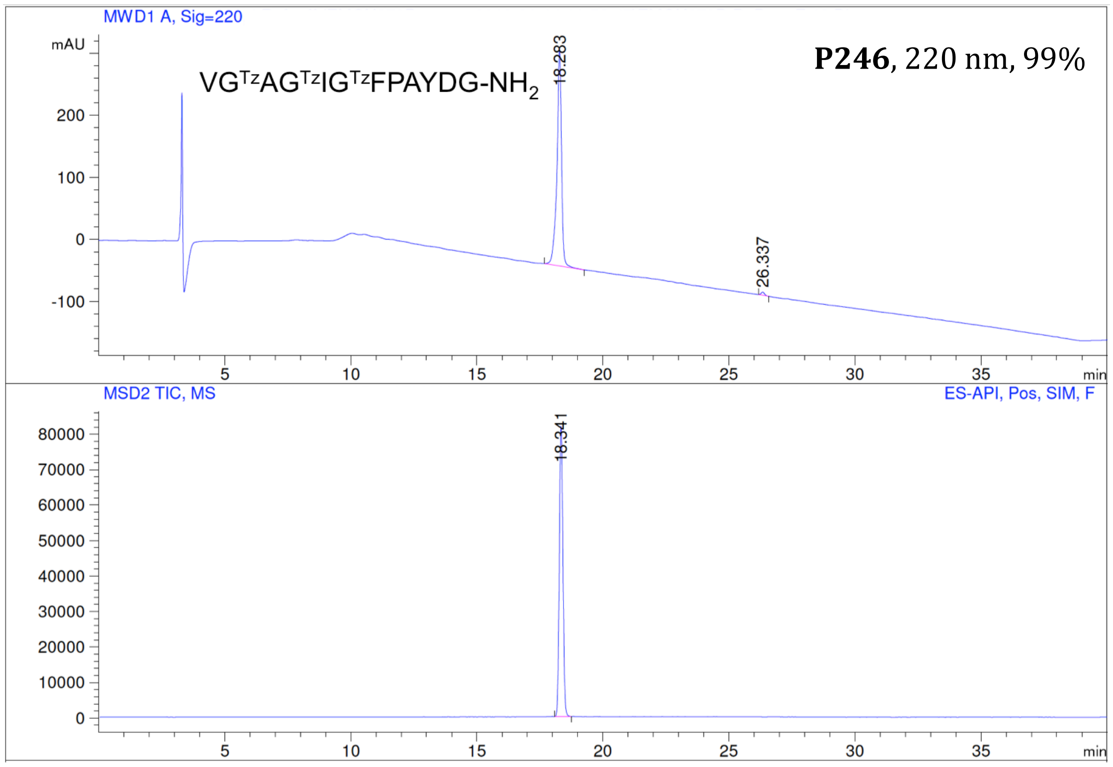
MWD1 A, Sig=220
mAU
VG$^{Tz}$AG$^{Tz}$IG$^{Tz}$FPAYDG-NH$_2$
**P246**, 220 nm, 99%
18.283
26.337
200
100
0
-100
5
10
15
20
25
30
35
min
MSD2 TIC, MS
ES-API, Pos, SIM, F
18.341
80000
70000
60000
50000
40000
30000
20000
10000
0
5
10
15
20
25
30
35
min

# XII. References


[1] G. R. Fulmer, A. J. M. Miller, N. H. Sherden, H. E. Gottlieb, A. Nudelman, B. M. Stoltz, J. E. Bercaw, K. I. Goldberg, *Organometallics* **2010**, *29*, 2176.

[2] J. Koehnke, A. Bent, W. E. Houssen, D. Zollman, F. Morawitz, S. Shirran, J. Vendome, A. F. Nneoyiegbe, L. Trembleau, C. H. Botting, M. C. M. Smith, M. Jaspars, J. H. Naismith, *Nat Struct Mol Biol* **2012**, *19*, 767.

[3] E. D. Goddard-Borger, R. V. Stick, *Organic Letters* **2007**, *9*, 3797.

[4] N. Fischer, E. D. Goddard-Borger, R. Greiner, T. M. Klapötke, B. W. Skelton, J. Stierstorfer, *The Journal of Organic Chemistry* **2012**, *77*, 1760-1764.

[5] Lundquist, J. C. Pelletier, *Organic Letters* **2001**, *3*, 781.

[6] H. D. Dickson, S. C. Smith, K. W. Hinkle, *Tetrahedron Letters* **2004**, *45*, 5597-5599.

[7] J.-A. Fehrentz, B. Castro, *Synthesis* **1983**, *1983*, 676.

[8] G. Reginato, A. Mordini, F. Messina, A. Degl'Innocenti, G. Poli, *Tetrahedron* **1996**, *52*, 10985.

[9] I. E. Valverde, A. Bauman, C. A. Kluba, S. Vomstein, M. A. Walter, T. L. Mindt, *Angew. Chem. Int. Ed.* **2013**, *52*, 8957.

[10] M. Tischler, D. Nasu, M. Empting, S. Schmelz, D. W. Heinz, P. Rottmann, H. Kolmar, G. Buntkowsky, D. Tietze, O. Avrutina, *Angew. Chem. Int. Ed.* **2012**, *51*, 3708.

[11] A. Tam, U. Arnold, M. B. Soellner, R. T. Raines, *J. Am. Chem. Soc.* **2007**, *129*, 12670.
